# Tubular epithelial cells promote macrophage pyroptosis through PANX1-extracellular ATP-P2X7 signaling in aldosterone-dependent renal injury

**DOI:** 10.1038/s41419-026-08951-3

**Published:** 2026-06-05

**Authors:** Chongjian Wang, Hongkun Ma, Jing Xu, Yan Tang, Jiayin Feng, Yilei Yang, Zhiyu Wang, Wen Zhang

**Affiliations:** Department of Nephrology, Ruijin Hospital, School of Medicine, Shanghai Jiao Tong University, Shanghai, PR China

**Keywords:** Chronic kidney disease, Hypertension, Chronic inflammation

## Abstract

Tubular epithelial cell (TEC)–macrophage crosstalk contributes to aldosterone (Aldo)-dependent renal fibrosis in hypertension. Extracellular ATP (eATP) can act as a mediator of TEC–macrophage crosstalk. However, whether and how eATP functions in Aldo-induced renal injury remain unknown. A deoxycorticosterone acetate (DOCA)-salt-induced hypertensive mouse model and a primary mouse renal tubular epithelial cell (mTEC)–bone marrow-derived macrophage (BMDM) coculture model were developed. To investigate the relationship between Aldo/MR and eATP signaling in TEC–macrophage crosstalk, specific inhibitors, adeno-associated virus (AAV)-shRNA and small interfering RNA (siRNA) were used. scRNA-seq analysis of kidneys from DOCA-salt mice was performed for verification. Urinary ATP levels and renal pannexin1 (PANX1, an ATP efflux channel) expression increased significantly in DOCA-salt mice, whereas the selective mineralocorticoid receptor (MR) antagonist finerenone treatment reduced urinary ATP levels, PANX1 expression, tubular injury, and macrophage-related inflammation. Inhibiting PANX1-eATP-P2X7 signaling with the PANX1 inhibitor probenecid, specifically knocking down tubular PANX1, or using the eATP ectonucleotidase apyrase or the P2X7 blocker AZ10606120 mitigated renal tubular damage and NLRP3-mediated pyroptosis in the renal cortex of DOCA-salt mice. Similarly, in mTECs, finerenone suppressed Aldo-induced ATP release and PANX1 upregulation. A luciferase assay confirmed the binding site of MR to the PANX1 transcription promoter. Coculture of BMDMs with mTECs under Aldo stimulation significantly increased NLRP3-mediated BMDM pyroptosis and migration. However, these effects were reversed by treating the mTECs with MR or PANX1 siRNA. Similar results were observed after treatment with apyrase or AZ10606120 in the coculture system. scRNA-seq analysis further identified the activation of P2X7/NLRP3-related pyroptosis pathway in macrophages from DOCA-salt mice. In conclusion, TECs play a critical role in macrophage pyroptosis in Aldo-induced renal injury. eATP is a key messenger involved in TEC–macrophage crosstalk. PANX1-eATP-P2X7 signaling mediates TEC‒macrophage interactions to exacerbate Aldo-driven renal inflammation in hypertension.

**Figure:**
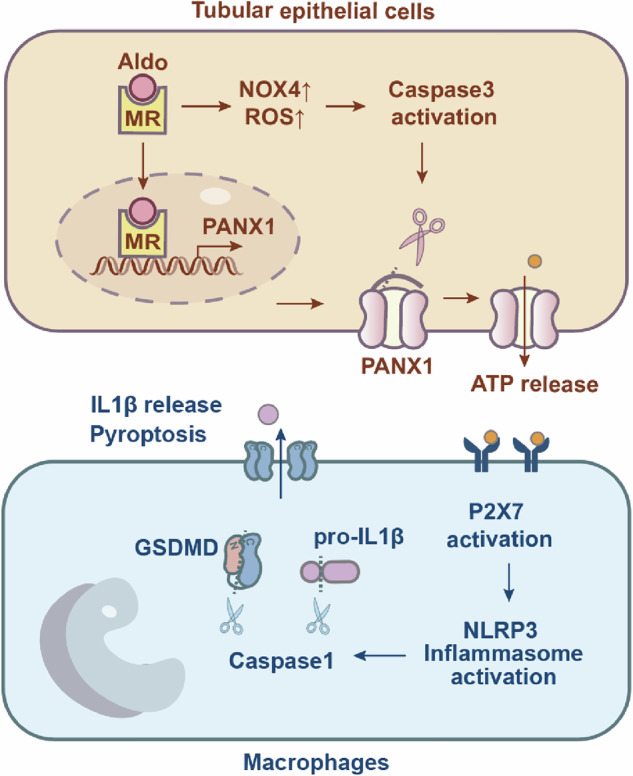
Mechanism through which Aldo/MR-induced PANX1-eATP-P2X7 signaling activation promotes macrophage pyroptosis in hypertensive renal injury.

Mechanism through which Aldo/MR-induced PANX1-eATP-P2X7 signaling activation promotes macrophage pyroptosis in hypertensive renal injury.

## Introduction

Hypertension is a global public health problem with a high prevalence [1]. Hypertension-induced renal injury is one of the most common causes of chronic kidney disease (CKD) and end-stage renal disease [2, 3]. Overactivation of the renin-angiotensin-aldosterone system (RAAS) is a central pathological feature of hypertension-induced renal injury. However, the use of angiotensin-converting enzyme inhibitors (ACEIs) and angiotensin II receptor blockers (ARBs) induces the “aldosterone breakthrough” phenomenon [4], and excessive aldosterone (Aldo) further contributes to a progressive decline in renal function [5, 6]. Mechanistically, Aldo enters cells and binds to the mineralocorticoid receptor (MR) to induce transcriptional and nongenomic signaling. Tubular epithelial cells (TECs) are the main targets of Aldo in the kidney. Aldo/MR activation can trigger sodium and water retention to exacerbate hypertension [7, 8] and simultaneously induce oxidative stress injury in TECs [9, 10].

Macrophage-mediated inflammation is among the most important characteristics of hypertension-induced CKD. Aldo/MR activation facilitates kidney fibrosis by targeting macrophages, either directly or by altering epithelial cell crosstalk [11]. Injured TECs can recruit macrophages and induce subsequent phenotypic transition to exacerbate renal injury through the secretion of chemokines, the expression of adhesion molecules [12, 13], or the release of damage-associated molecular patterns (DAMPs) [14, 15]. Adenosine triphosphate (ATP) has been reported to be the earliest increased DAMP and an important paracrine molecule in hypertension [16]. Extracellular ATP (eATP) acts as a mediator of TEC–macrophage crosstalk in ischemia/reperfusion (I/R)-induced acute kidney injury (AKI) model [17, 18] and unilateral ureteral obstruction model [19]. Yet how ATP was released to the interstitium and whether eATP mediates the crosstalk between TECs and macrophages under Aldo stress in hypertension-induced renal injury remains unknown.

In this study, we used a deoxycorticosterone acetate (DOCA)-salt hypertensive mouse model and a primary mouse renal tubular epithelial cell (mTEC)-bone marrow-derived macrophage (BMDM) coculture model. We report that Aldo/MR activation promoted the release of ATP from TECs by activating PANX1 (an ATP release membrane channel) and subsequently induced P2X7 (an eATP receptor)-mediated macrophage pyroptosis in Aldo-dependent renal injury. Our findings reveal the adverse effect of TEC–macrophage crosstalk on Aldo-dependent renal fibrosis and demonstrate the potential value of decreasing tubule-released ATP to mitigate Aldo-dependent renal injury.

## Materials and methods

### DOCA-salt-treated mouse model and drug treatment

Male C57BL/6 mice aged 8–10 weeks were purchased from Charles River Laboratories (Beijing, China) and housed at an optimal temperature of 22 ± 2 °C under a 12 h light‒dark cycle. All the mice had free access to water and food. No blinding was done for animal studies. The animal sample size was selected on the basis of previous similar studies [20]. and the animals were randomly assigned to individual groups. For the DOCA-salt model, the mice were anesthetized with isoflurane (induction: 5%, maintenance: 2%), and the left kidney was removed. One week after nephrectomy, the mice in the DOCA-salt group were implanted with 50 mg of DOCA pellets (Innovative Research of America, Sarasota, FL) subcutaneously and received drinking water containing 1% NaCl for 21 days. The mice in the sham group received a sham surgery and normal drinking water and were not implanted with the DOCA pellets. For drug treatment, one week after DOCA implantation, the DOCA-salt mice received finerenone (3 mg/kg/d and 10 mg/kg/d according to previous studies [21, 22], HY-111372; MedChemExpress; Monmouth Junction, NJ, USA) or vehicle by oral gavage for 2 weeks, and the sham group mice received vehicle simultaneously. Probenecid (PBN, 50 mg/kg/d and 100 mg/kg; HY-B0545; MedChemExpress) was administered by intraperitoneal injection for 2 weeks. Apyrase (0.2 U/g/d and 0.4 U/g/d; HY-P2764; MedChemExpress) and AZ10606120 (500 µg/kg/d and 1 mg/kg/d; HY-108669; MedChemExpress) were also administered by intraperitoneal injection for 2 weeks. The mice that completed treatment and survived were used for further analysis. After treatment was completed, the mice were sacrificed under anesthesia, and urine and kidney tissues were collected for further analysis.

### Adeno-associated virus (AAV) renal pelvis injection

AAV vectors expressing eGFP and either a PANX1-targeting shRNA (ACAAGAUGGUCACAUGUAU) or a negative control shRNA (GTTCTCCGAACGTGTCACGT) under the control of the TEC-specific promoter KSP were provided by GenePharma (Shanghai, China). The AAV concentration was 1 × 10^13^ vg/mL. For renal pelvis injection, 20 µL of the AAV solution was rapidly injected into the renal pelvis using an insulin syringe needle, and the needle was held in place for 30 s after injection. Knockdown efficiency was evaluated 2 weeks post-injection by western blotting for PANX1 expression and by immunofluorescence analysis for eGFP intensity. After successful AAV injection, the mice were assigned to either the DOCA‑salt group or the sham group to undergo the respective surgical procedures.

### Cell culture and treatments

Mouse primary tubular epithelial cells (mTECs) were obtained as previously described [23]. First- and second-generation mTECs were used. The cells were stimulated with Aldo (100 nM; HY-113313; MedChemExpress) for 3 to 12 h as indicated and treated with finerenone (0.1, 0.2, 0.5 and 1 mM), z-DEVD-fmk (100 µM; HY-12466; MedChemExpress), PBN (0.1, 0.5, 1 and 2 mM), apyrase (0.1, 0.2, 0.5 and 1 U/mL) or AZ10606120 (10, 20, 50, 100 µM) as described.

Mouse bone marrow-derived macrophages (BMDMs) were isolated and cultured as described previously [24]. For CCK-8 assay, BMDMs were treated with apyrase (0.1, 0.2, 0.5, and 1 U/mL) and AZ10606120 (10, 20, 50, and 100 µM). To coculture mTECs and BMDMs, mTECs were seeded into 0.4 μm-pore-size transwell inserts (Jet Bio-Filtration, Guangzhou, China) and placed into 6-well plates where BMDMs were initially seeded. To simulate the renal environment, the mTECs culture medium was used in the coculture system. The coculture system was stimulated with Aldo (100 nM) for 6 h. Apyrase (0.5 U/mL) was added to the coculture system simultaneously with Aldo, while AZ10606120 (50 µM) was added 1 h before Aldo stimulation.

### Blood pressure measurement

The blood pressure of the mice was measured noninvasively via the tail-cuff method using the BP-2000 Blood Pressure Analysis System (Visitech Systems, Inc., North Carolina, USA) as previously described [25].

### Urinary albumin measurement

Mouse urine samples were collected and centrifuged at 2000 rpm and 4 °C for 10 min to obtain the supernatant. The urinary albumin levels of the mice were measured using a Mouse ALB (Albumin) ELISA Kit (E-EL-M3032; Elabscience, Wuhan, China), and urinary creatinine levels were simultaneously measured (measured by a QuantiChrom Creatinine Assay Kit; DICT-500; Bioassay Systems; Hayward, CA, USA) to normalize the results.

### Histological and immunohistochemistry analysis

Mouse kidney samples were fixed in 4% paraformaldehyde for 24 h, embedded in paraffin, and cut into 4 μm thick sections. Kidney sections stained with hematoxylin‒eosin (H&E) were observed under a microscope (200BX51; Olympus Corp., Tokyo, Japan) by two pathologists.

For renal fibrosis assessment, kidney sections were analyzed with Sirius red staining, and ten images were randomly captured under a 200× microscope for collagen-positive area analysis by ImageJ (version 1.53; National Institutes of Health, Bethesda, MD, USA).

Kidney sections were stained with a F4/80 antibody (70076, 1:400; Cell Signaling Technology, CST; Danvers, MA, USA) followed by HRP-conjugated goat anti-rabbit IgG. At least ten images (200×) were used for semiquantitative analysis by ImageJ.

### DHE staining

The frozen kidney slides were incubated with dihydroethidium (DHE; D7008-10, 1:400; Sigma‒Aldrich) at room temperature for 1 h and counterstained with DAPI. Quantification of fluorescence intensity based on five sections of each sample was performed using ImageJ.

### Immunofluorescence analysis

Kidney sections were incubated with primary antibodies against P2X7 (1:2 000, ab259942, Abcam, Discovery Drive, Cambridge, UK), F4/80 (1:4 000, 70076, CST), CD86 (1:400, 19589, CST) and CD206 (1:2 000, 24595, CST), followed by the corresponding fluorescent secondary antibodies. DAPI (D9542; Sigma‒Aldrich) was used for nuclear staining. The sections were observed under a fluorescence microscope (BX53, OLYMPUS, Japan), and ten sections were captured for analysis by ImageJ.

### ATP measurement

ATP levels in mouse urine and cell culture medium were measured using a luciferase-based ATP bioluminescence assay kit (S0027; Beyotime) according to the manufacturer’s instructions with a multimode reader (Synergy Neo2; Biotek, VT, USA) and corrected for the creatinine level or BCA results of the same sample.

### TUNEL staining

A TUNEL BrightRed Apoptosis Detection Kit (A113; Vazyme, Nanjing, China) was used to stain kidney sections for TUNEL. The nuclei were then counterstained with DAPI. The sections were observed under a fluorescence microscope, and ten sections were captured for analysis by ImageJ.

### Cell viability assay

The viability of BMDMs and mTECs following drug treatment was assessed using a Cell Counting Kit-8 (CCK-8; CK04; Dojindo Laboratories, Japan). Cells seeded in 96-well plates were exposed to the indicated concentrations of drugs for 6 h. CCK-8 reagent was then added, and after 2 h of incubation, the absorbance was measured at 450 nm. The cell viability is expressed as a percentage of that of the untreated control.

### Dual-luciferase reporter assay

Immortalized human tubular epithelial cells (HK2) were cultured as previously reported [25]. The cell lines were mycoplasma free and authenticated by STR identification. Wild-type (WT) PANX1 containing the predicted binding site of MR and its mutant type with this site deleted (MUT) were designed and synthesized by GenePharma (the sequences of the plasmids are provided in the supplementary materials). The transfection of HK2 was conducted using LipoMax 3000 Transfection Reagent (T103; Witcel, Shanghai, China). A dual-luciferase reporter gene assay kit (RG088S; Beyotime) was used to determine the Luc/Ren ratio after stimulation with Aldo at 100 nM for 12 h.

### Caspase 3 activity measurement

A Caspase 3 activity kit (C1116; Beyotime) with a colorimetric substrate (Ac-DEVD-pNA) was used according to the manufacturer’s instructions. The level of Caspase 3 activity was corrected to the total protein level by the Bradford method.

### ROS measurement

Cells were collected in FACS tubes and stained by a Photo-oxidation Resistant DCFH-DA-dye (R253; Dojindo Laboratories) at 37 °C for 30 min, followed by analysis on a flow cytometer (CytoFLEX; Beckman Coulter, CA, USA), and the data were analyzed with FlowJo software (version 10.4; Tree Star, Ashland, USA).

### YO-PRO-1 uptake assay

The cells were incubated with YO-PRO-1 (C2022; Beyotime) at 37 °C for 0.5 h according to the manufacturer’s instructions. Images were captured using an Olympus optical microscope (BX53; OLYMPUS, Japan) for quantification.

### Lactate dehydrogenase (LDH) release measurement

After stimulation, the BMDM culture medium was centrifuged at 2000 rpm and 4 °C for 5 min, and the supernatant was collected for analysis. An LDH Cytotoxicity Assay Kit (C0016; Beyotime) was used according to the manufacturer’s instructions. The LDH level was normalized to the corresponding BCA results of the BMDM samples and is expressed as the fold change relative to that of the control group.

### IL-1β measurement

IL-1β levels in the BMDM culture supernatants were measured by a Mouse IL-1β High Sensitivity ELISA Kit (EK201BHS; Multi Science, Hangzhou, China) according to the manufacturer’s instructions, normalized to the corresponding BCA results of the BMDM samples, and expressed as the fold change relative to those of the control group.

### Migration assay

BMDMs were seeded into 8.0 μm-pore size transwell inserts (Jet Bio-Filtration) and cocultured with mTECs for 24 h. After stimulation, the BMDMs were fixed and stained with crystal violet staining solution (G1014; Servicebio). After the cells on the upper surface were removed, five images of each membrane (100×) were captured using an Olympus optical microscope (BX53, OLYMPUS, Japan) for quantification.

### Transfection experiment

The negative control (NC) siRNA (sense: 5′-UUCUCCGAACGUGUCACGUTT-3′, antisense: 5′-ACGUGACACGUUCGGAGAATT-3′), Nr3c2 siRNA (sense: 5′- GAGGAGUACUCCAUAAUGATT-3′, antisense: 5′-UCAUUAUGGAGUACUCCUCTT-3′) and PANX1 siRNA (sense: 5′- ACAAGAUGGUCACAUGUAUTT-3′, antisense: 5′-UACAUGUGACCAUCUUGUTT-3′) were designed and provided by GenePharma. Above siRNAs were transfected into mTECs or BMDMs via WitLNP RNAi (T107, Witcel) as the manufacturer’s protocol recommendation. Forty-eight hours after transfection with siRNA, we assessed the protein silencing effect via western blotting.

### RT‒qPCR

Total RNA from renal tissues and cells was extracted using a quick RNA extraction kit (AG21023; Accurate Biology, Hunan, China). Complementary DNA was generated using a cDNA synthesis kit (R323, Vazyme, Jiangsu, China), and qPCR was performed using SYBR Green (AG11702, Accurate Biology). The specific primers used were as follows: Mouse *Actb*, (F) GTGACGTTGACATCCGTAAAGA, (R) GTAACAGTCCGCCTAGAAGCAC; Mouse *Panx1*, (F) CCACCGAGCCCAAGTTCAA, (R) GGAGAAGCAGCTTATCTGGGT; Fn1, (F) ATGGTACAGCTGATCCTGCC, (R) GCCCTGGTTTGTACCTGCTA; Vim, (F) GGATCAGCTCACCAACGACA, (R) GGTCAAGACGTGCCAGAGAA; *Ccl2*, (F) GTAGGTTCTGATCTCATTTG, (R) CAGCAAGATGATCCCAATGA; *Il6*, (F) TAGTCCTTCCTACCCCAATTTCC, (R) TTGGTCCTTAGCCACTCCTTC; *Cd86*, (F) GATGGACCCCAGATGCACCAT, (R) ACCAGCTCACTCAGGCTTATG; and *Mrc1*, (F) TCTTTGCCTTTCCCAGTCTCC, (R) TGACACCCAGCGGAATTTC.

Relative mRNA transcript levels were based on 2^−∆Ct^ normalized to *Actb* and compared with those of the controls.

### Western blotting

The protein in frozen kidney tissues from mice, mTECs and BMDMs was extracted using RIPA buffer (20–188; Sigma–Aldrich) containing ProtLytic Protease and Phosphatase Inhibitor Cocktail (P002; New Cell & Molecular Biotech Co., Ltd., Jiangsu, China). The following primary antibodies were used: anti-β-actin (AS014, 1:2500, Sigma‒Aldrich), anti-NOX4 (14347-1-AP, 1:1000, Proteintech), anti-cleaved Caspase3 (19677-1-AP, 1:1 000, Proteintech), anti-PANX1 (91137, 1:1000, CST), anti-P2X7 (Ab259942, 1:2000, Abcam), anti-NLRP3 (15101, 1:1000, CST), anti-Caspase 1 (A25308, 1:1000, ABclonal, Wuhan, China), anti-GSDMD (ab219800, 1:2000, Abcam), and anti-IL-1β (P50520-1R1, 1:1000, Abmart, Shanghai, China). The secondary antibodies used were anti-rabbit IgG, an HRP-linked antibody (A5316; 1:4000; ABclonal), and anti-mouse IgG, an HRP-linked antibody (7076; 1:2500; CST). The intensity of the bands was determined with ImageJ. Relative protein levels were normalized to those of β-actin and are expressed as the fold change compared with those in the control group.

### Single cell suspension preparation and scRNA-seq data processing

The freshly collected kidneys from the sham mice and DOCA-salt mice were used to prepare single-cell suspensions as previously reported [26]. The FASTQ files were processed and aligned to GRCm39 mouse reference genome using Cell Ranger software (version 9.0.0) from 10x Genomics, with unique molecular identifier (UMI) counts summarized for each barcode. The UMI count matrix was then analyzed using Seurat (version 4.0.0) R package. Cells were filtered by gene numbers (gene numbers < 170), UMI (UMI < 500), percentage of mitochondrial RNA UMIs (proportion of UMIs mapped to mitochondrial genes > 50%) and percentage of hemoglobin RNA UMIs (proportion of UMIs mapped to hemoglobin genes > 5%). The DoubletFinder package (version 2.0.3) was used to identify potential doublets. To obtain the normalized gene expression data, library size normalization was processed using the NormalizeData function. Specifically, the global-scaling normalization method “LogNormalize” normalized the gene expression measurements for each cell by the total expression, multiplied by a scaling factor, and log-transformed the results. The highly variable genes were identified using the Seurat function FindVariableGenes. Principal-component analysis (PCA) was performed to reduce the dimensionality with RunPCA function. Then, the RunHarmony function in harmony (version 1.0) R package was performed to remove the batch effects. Graph-based clustering was performed to cluster cells according to their gene expression profile with the FindClusters function. Cells were visualized using a 2-dimensional Uniform Manifold Approximation and Projection (UMAP) algorithm with the RunUMAP function. The FindAllMarkers function was used to identify marker genes of each cluster and select differentially expressed genes (DEGs). KEGG pathway enrichment analysis of DEGs were respectively performed using R (version 4.0.3) based on the hypergeometric distribution. The sequencing and bioinformatics analysis were provided by OE Biotech Co., Ltd. (Shanghai, China).

### Statistical analysis

Statistical analysis was performed using GraphPad 9.0 (GraphPad Software, La Jolla, CA, USA). The distribution of the data was assessed using the Shapiro‒Wilk test, and the variances were assessed by Brown–Forsythe test. The results are presented as the means ± SDs. Two-tailed, unpaired Student’s *t*-tests were used when two groups were compared. One-way ANOVA followed by Tukey’s test for multiple comparisons was used when there were more than two groups. Two-way ANOVA followed by Tukey’s test for multiple comparisons was used to analyze the effects of two independent variables when multiple group comparisons were performed. The number of animals or measurements in each group is indicated in the figure legends. A value of *p* < 0.05 was considered to indicate statistical significance.

## Results

### The mitigation of DOCA-salt-induced renal injury and inflammation by MR antagonists is independent of hemodynamic changes

**Fig. 1:**
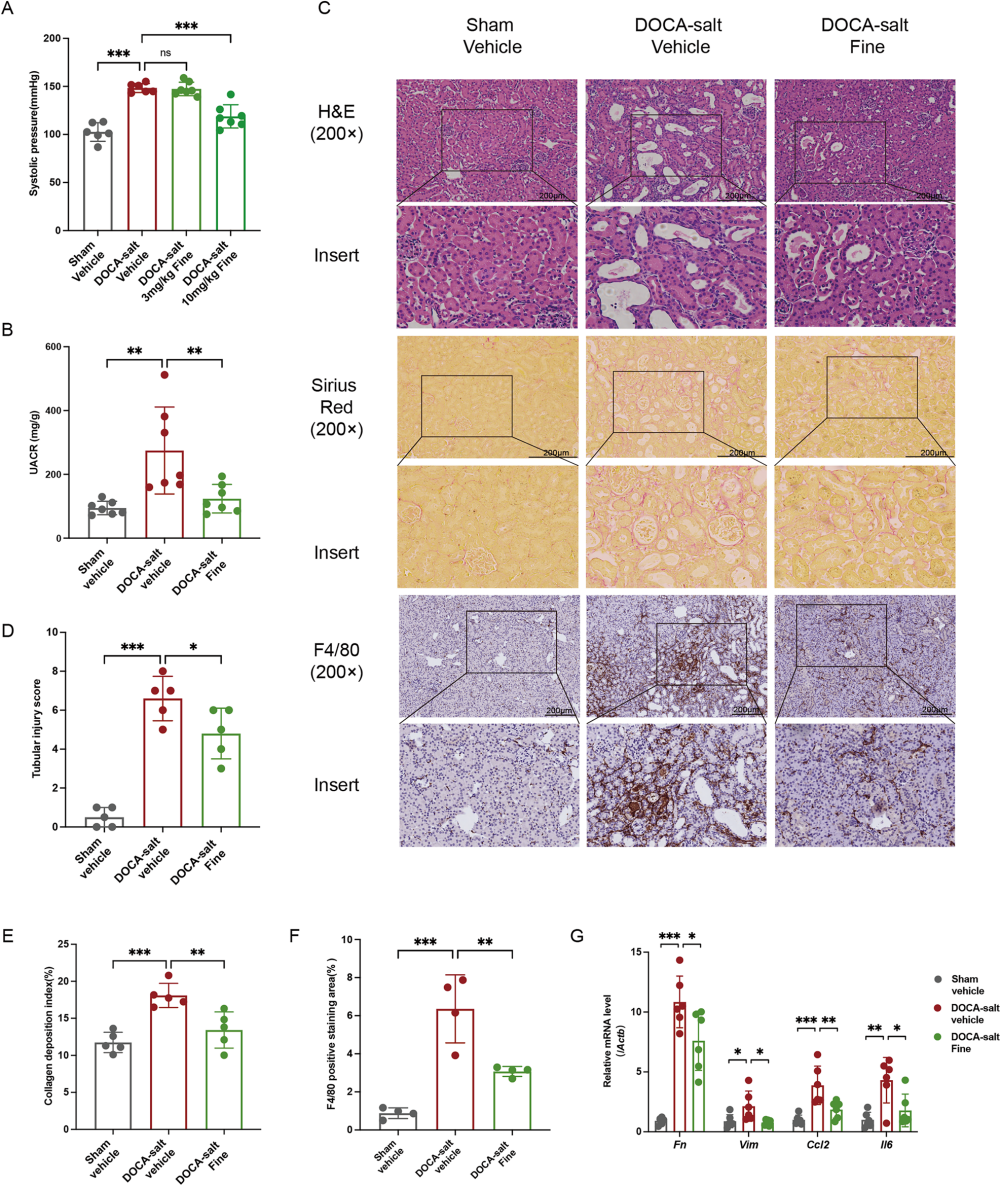
Finerenone mitigates DOCA-salt-induced renal injury and inflammation independent of blood pressure. **A** Changes in the systolic pressure of DOCA-salt mice treated with finerenone (Fine, 3 mg/kg/d and 10 mg/kg/d, by oral gavage); *n* = 6–7 per group. **B** Changes in the urinary albumin/creatinine ratio (UACR) in DOCA-salt mice after finerenone treatment (3 mg/kg/d); *n* = 7 per group. **C** Representative images and magnified views of H&E staining, Sirius red staining, and F4/80 staining images of kidney sections; magnification: 200×; scale bar: 200 µm. **D** Quantitative data of the tubular injury score; *n* = 5 per group. **E** Quantitative data of the collagen deposition index (%) in the kidneys; *n* = 5 per group. **F** Quantitative data of the F4/80-positive area (%) in the kidneys; *n* = 4 per group. **G** The mRNA levels of *Fn*, *Vim*, *Ccl2* and *Il6* in the kidney were determined by RT‒qPCR; *n* = 6 per group. ^ns^*p* > 0.05, *p < 0.05, **p < 0.01, and ***p < 0.001 by one-way ANOVA with Tukey’s test for multiple comparisons.

A DOCA-salt-induced hypertensive mouse model was chosen because of persistent MR activation. The selective MR antagonist finerenone was used to treat DOCA-salt mice. As shown in Fig. 1A, the systolic blood pressure (SBP) of the DOCA-salt mice significantly increased. High-dose finerenone treatment reduced the SBP to 29 mmHg, whereas low-dose finerenone treatment minimally affected the SBP. To exclude renoprotection due to hemodynamic changes, we used a low dose of finerenone in subsequent experiments. As shown in Fig. 1B, finerenone significantly reduced the urinary albumin-to-creatinine ratio (UACR) of the DOCA-salt mice. It also attenuated renal tubular injury and fibrosis (Fig. 1C), as manifested by a reduced tubular injury score (Fig. 1D) and collagen deposition (Fig. 1E) and decreased macrophage infiltration (Fig. 1F) in renal cortex tissue. Moreover, the mRNA levels of the fibrosis gene fibronectin (*Fn*), the epithelial–mesenchymal transition (EMT) marker vimentin (*Vim*), the chemokine *Ccl2*, and the inflammatory cytokine *Il6* were reduced in the finerenone-treated group (Fig. 1G). In addition, finerenone mitigated renal oxidase stress, as shown by decreased NADPH oxidoreductase 4 (NOX4) expression and DHE staining intensity in the kidney (sFig. 1A, B). The apoptosis was slightly increased after DOCA-salt treatment, as indicated by the increase of cleaved Caspase-3 (c-Casp-3) and TUNEL staining (sFig. 1B, C). This increase was inhibited by finerenone treatment (sFig. 1B, C). The results above reveal that MR activation resulted in macrophage infiltration and renal fibrosis.

### MR antagonist reduces extracellular ATP levels, pannexin 1 (PANX1) expression, and P2X7-mediated macrophage pyroptosis in DOCA-salt mice

**Fig. 2:**
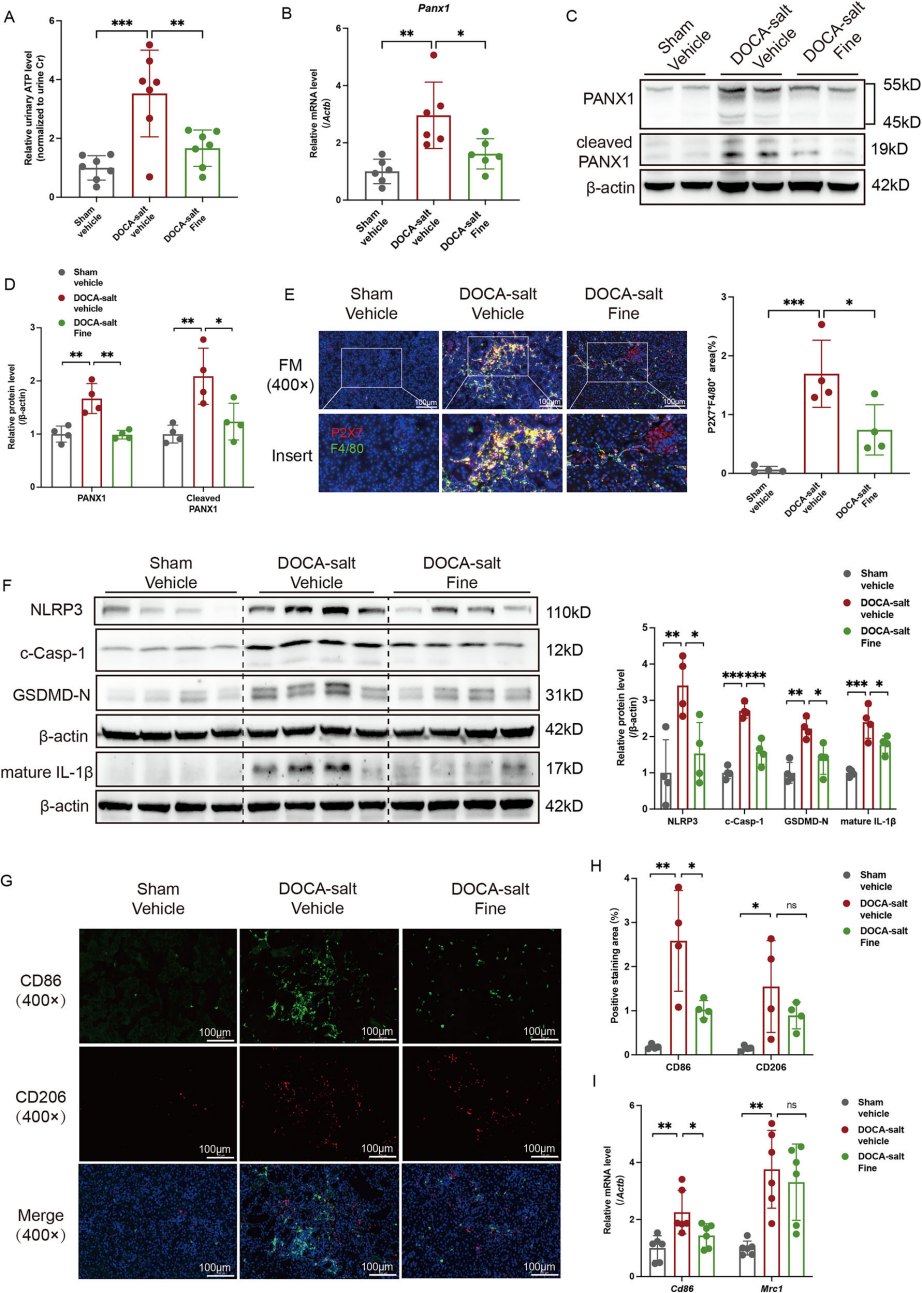
Finerenone mitigates NLRP3-related pyroptosis by reducing PANX1-mediated ATP release in DOCA-salt mice. **A** Relative urinary ATP level, corrected by the urinary creatinine level; *n* = 7 per group. **B** Changes in *Panx1* mRNA levels in the renal cortex; *n* = 6 per group. **C** Representative western blotting images and **D** summarized data showing changes in total and cleaved PANX1 expression in the renal cortex; *n* = 4 per group. **E** Representative fluorescence images, magnified views, and summarized data of the percentage area of P2X7 (red)-F4/80 (green) dual-positive cells in the kidney; magnification: 400×; scale bar: 100 µm; *n* = 4 per group and 10 independent fields per section. **F** Representative western blotting images and quantitative data for NLRP3 inflammasome-related pyroptosis proteins, including NLRP3, cleaved Caspase-1 (c-Casp-1), GSDMD N-terminal, and mature IL-1β, in the renal cortex; *n* = 4 per group. **G** Representative fluorescence images and magnified views of the changes in the M1 macrophage marker CD86 and M2 macrophage marker CD206 in the kidney and **H** summarized data of CD86- and CD206-positive staining areas (%) in the kidney; magnification: 400×; scale bar: 100 µm; *n* = 4 per group and 10 independent fields per section. **I** The mRNA levels of *Cd86* and *Mrc1* in the kidney; *n* = 6 per group. ^ns^*p* > 0.05, *p < 0.05, **p < 0.01, and ***p < 0.001 by one-way ANOVA with Tukey’s test for multiple comparisons.

A significant increase in urinary ATP was observed in the DOCA-salt mice, which was suppressed by finerenone (Fig. 2A). Connexin 43 (Cx43, *Gja1*) and PANX1 are the two main channels for ATP efflux [27, 28]. Cx43 mRNA expression did not significantly change (sFig. 2A), but the mRNA level of PANX1 significantly increased in the DOCA-salt group (Fig. 2B). Moreover, increased total or activated (shown by cleaved fragment) PANX1 protein levels were observed in the renal cortex of DOCA-salt mice (Fig. 2C, D). Notably, these changes were significantly inhibited by finerenone treatment (Fig. 2B–D). A Nephroseq dataset of the tubulointerstitium was used for further analysis of Cx43 and PANX1 mRNA expression in patients with hypertensive renal injury compared with healthy living donors. No change in the expression of the Cx43 mRNA but an increasing trend in the expression of the PANX1 mRNA was observed (sFig. 2B). Furthermore, the PANX1 mRNA level was positively correlated with the serum creatinine level (sFig. 2C).

P2X7 is the most involved in inflammation among P2 receptors, which are expressed by macrophages mainly in the kidney [29, 30]. A critical increase in P2X7-positive macrophages in the renal cortex of DOCA-salt mice was observed by immunofluorescence staining (Fig. 2E). The protein levels of NLRP3 and its downstream effectors, including cleaved Caspase-1 (c-Casp-1), Gasdermin-D N-terminal (GSDMD-N), and mature IL1β, largely increased in the renal cortex of DOCA-salt mice (Fig. 2F). Notably, these changes were significantly inhibited by finerenone treatment (Fig. 2E, F). Moreover, finerenone specifically mitigated the infiltration of M1 macrophages, as evidenced by the reduced number of CD86-positive macrophages, but no change in the number of CD206 (*Mrc1)*-positive macrophages was observed (Fig. 2G–I). These results suggest that Aldo/MR may trigger PANX1 to release intracellular ATP to the tubulointerstitium and induce macrophage pyroptosis through the eATP receptor P2X7 in DOCA-salt-induced hypertension.

### PANX1 inhibition or knockdown reduces renal ATP release and NLRP3-related pyroptosis in DOCA-salt mice

**Fig. 3:**
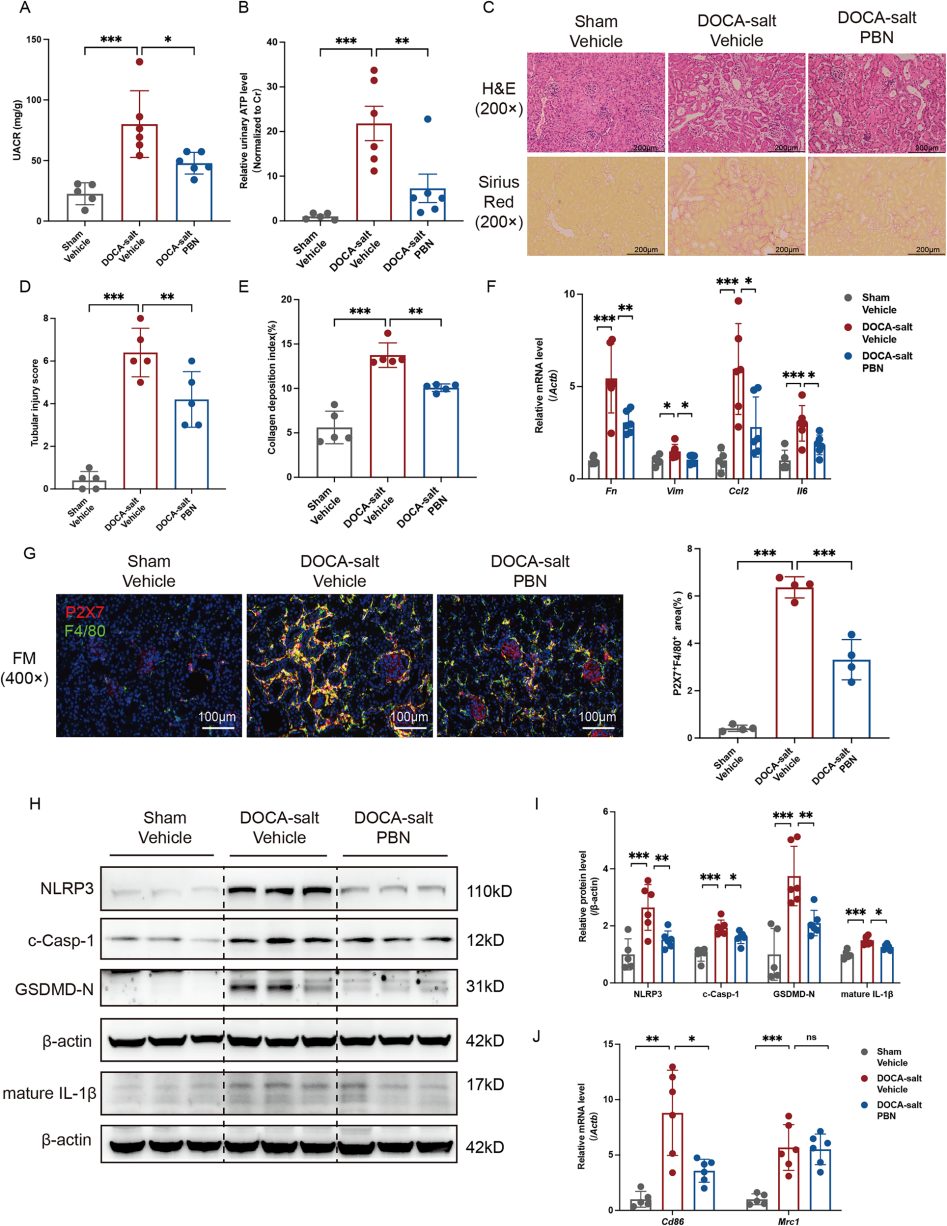
Probenecid suppresses ATP release to attenuate NLRP3-related pyroptosis and renal injury in DOCA-salt mice. **A** Changes in the urinary albumin/creatinine ratio (UACR) in DOCA-salt mice following probenecid (PBN, 50 mg/kg/d, by intraperitoneal injection) treatment; *n* = 5 in sham vehicle group and *n* = 6 in DOCA-salt vehicle and DOCA-salt PBN group. **B** Urinary ATP/creatinine levels (fold change); *n* = 5 in sham vehicle group and *n* = 6 in DOCA-salt vehicle and DOCA-salt PBN group. **C** Representative H&E and Sirius red images of kidney sections (magnification: 200×; scale bar: 200 µm). Quantitative data of **D** the tubular injury score (*n* = 5 per group) and **E** the collagen deposition index (%, *n* = 5 per group). **F** mRNA levels of *Fn*, *Vim*, *Ccl2,* and *Il6* in the renal cortex; *n* = 5 in sham vehicle group and *n* = 6 in DOCA-salt vehicle and DOCA-salt PBN group. **G** Representative images of P2X7 (red)-F4/80 (green) immunofluorescence staining and quantitative data of the percentage area of P2X7 and F4/80 dual-positive cells; magnification: 400×; scale bar: 100 µm; *n* = 4 per group; 10 independent fields per section were used for statistical analysis. **H** Representative western blotting images and **I** quantitative data for pyroptosis-related proteins, including NLRP3, cleaved Caspase-1 (c-Casp-1), GSDMD N-terminal, and mature IL-1β in the kidney; *n* = 5 in sham vehicle group and *n* = 6 in DOCA-salt vehicle and DOCA-salt PBN group. **J** mRNA levels of *Cd86* and *Mrc1* in the renal cortex; *n* = 5 in sham vehicle group and *n* = 6 in DOCA-salt vehicle and DOCA-salt PBN group. ^ns^p > 0.05, *p < 0.05, **p < 0.01, and ***p < 0.001 by one-way ANOVA with Tukey’s test for multiple comparisons.

PBN has been identified to specifically suppress PANX1-mediated tissue inflammation [31]. Given the potential blood pressure‑lowering effect of PBN, we first examined its dose‑response. As shown in sFig. 3A, B, 100 mg/kg PBN reduced systolic blood pressure, whereas 50 mg/kg PBN had a protective effect on the kidney without affecting blood pressure. Therefore, the 50 mg/kg dose was used in subsequent experiments. Our data revealed that PBN treatment in DOCA-salt mice significantly reduced the UACR (Fig. 3A) and urinary ATP levels (Fig. 3B). It also attenuated tubular injury and renal interstitial collagen deposition (Fig. 3C–E) and reduced the mRNA expression of fibrosis, EMT, and inflammation genes (*Fn*, *Vim*, *Ccl2*, and *Il6*) (Fig. 3F). Reduced renal interstitial infiltration of P2X7-positive macrophages in the PBN-treated group was observed by immunofluorescence staining (Fig. 3G). Concomitantly, PBN suppressed the protein expression of NLRP3 and its downstream effectors (c-Casp-1, GSDMD-N, and mature IL-1β) in the renal cortex (Fig. 3H, I). The mRNA expression of the M1 macrophage marker *Cd86* was downregulated, while no change in the expression of the M2 marker CD206 (*Mrc1*) was observed after PBN treatment (Fig. 3J).

**Fig. 4:**
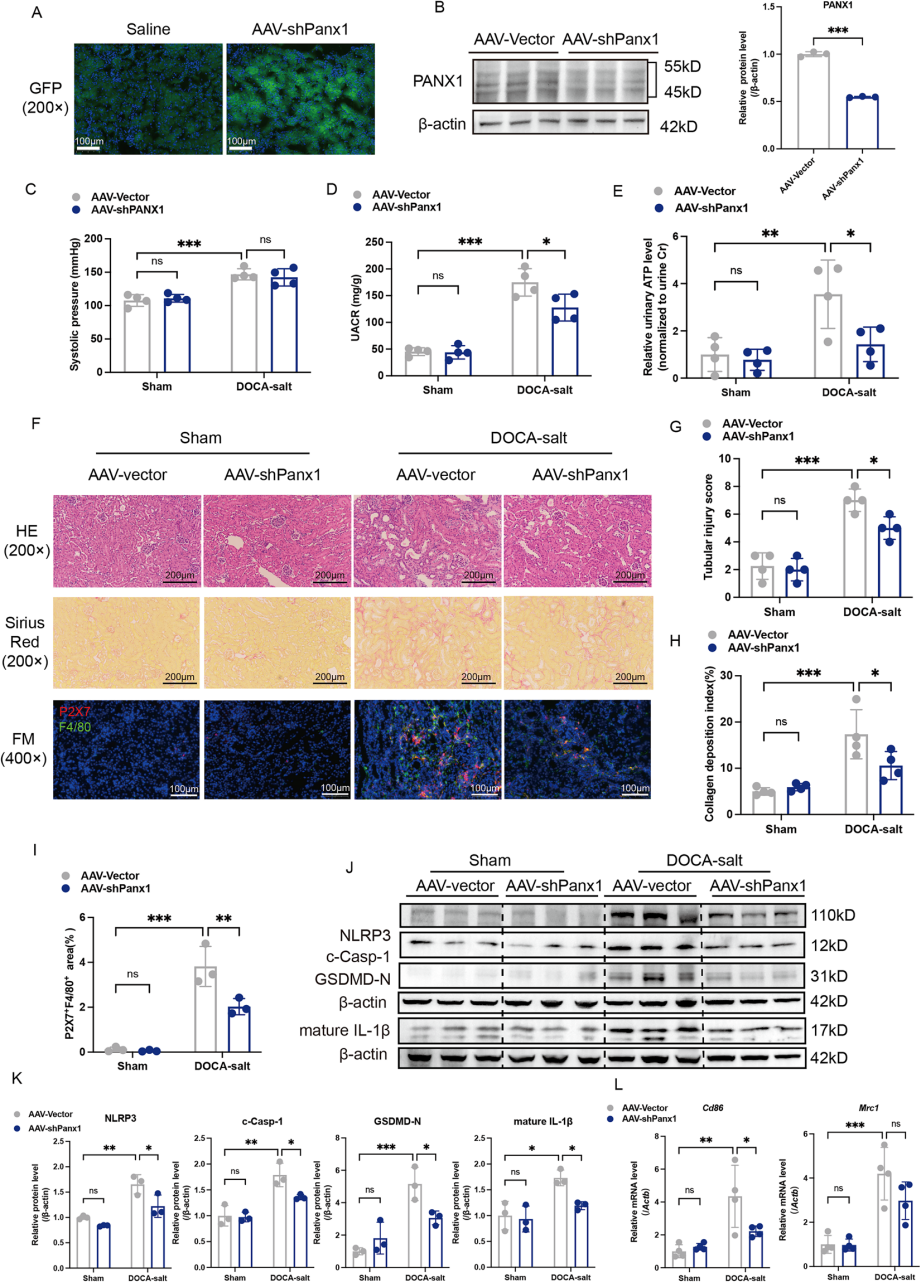
Tubular-specific PANX1 knockdown alleviated DOCA-salt-induced renal injury. **A** Representative image demonstrating the effect of KSP promoter-driven PANX1 shRNA AAV transfection, which expresses eGFP as a reporter. **B** Representative western blotting images and summary data showing PANX1 knockdown in renal cortex tissue following KSP promoter-driven shRNA AAV delivery. *n* = 3 per group; unpaired *t*-test. **C** Effect of tubular PANX1 knockdown on systolic blood pressure, **D** the urinary albumin/creatinine ratio (UACR) and **E** the urinary ATP/creatinine level in sham and DOCA-salt-treated mice; *n* = 4 per group; two-way ANOVA with Tukey’s test for multiple comparisons. **F** Representative images of H&E staining (magnification: 200×; scale bar: 200 µm), Sirius red staining (magnification: 200×; scale bar: 200 µm) and fluorescence images of P2X7 (red) and F4/80 (green) staining (magnification: 400×; scale bar: 100 µm) of kidney sections. **G** Summarized data of the tubular injury score and **H** collagen deposition index data (%); *n* = 4 per group; two-way ANOVA with Tukey’s test for multiple comparisons. **I** Summarized data of the area of P2X7-F4/80 dual-positive cells in the kidney; *n* = 3 per group; two-way ANOVA with Tukey’s test for multiple comparisons. **J** Representative western blotting images and **K** summarized data of pyroptosis-related proteins, including NLRP3, cleaved Caspase-1 (c-Casp-1), GSDMD N-terminal, and mature IL-1β, in the kidneys of the indicated groups; *n* = 3 per group; two-way ANOVA with Tukey’s test for multiple comparisons. **L** mRNA levels of *Cd86* and *Mrc1* in the kidneys of the indicated groups; *n* = 4 per group; two-way ANOVA with Tukey’s test for multiple comparisons. ^ns^*p* > 0.05, *p < 0.05, **p < 0.01, and ***p < 0.001.

To define the role of TEC PANX1, TEC-specific knockdown of PANX1 was achieved by injecting an AAV encoding a PANX1-targeting shRNA under the control of the KSP promoter via the renal pelvis (Fig. 4A, B). As shown in Fig. 4C, knockdown of PANX1 in TECs had little effect on systolic blood pressure. However, it significantly reduced renal albumin and ATP release (Fig. 4D, E) and alleviated renal injury and tubulointerstitial fibrosis (Fig. 4F–H) in DOCA-salt-treated mice. Notably, after TEC-specific PANX1 knockdown in DOCA-salt mice, the infiltration of P2X7-positive macrophages was reduced (Fig. 4F, I), and the levels of pyroptosis‑related proteins (NLRP3, c-Casp-1, GSDMD‑N, and mature IL‑1β) decreased in the renal cortex (Fig. 4J, K). RT–qPCR results revealed reduced *Cd86* mRNA levels upon TEC PANX1 knockdown, but *Mrc1* mRNA levels were not significantly affected (Fig. 4L).

### Inhibition of eATP-P2X7 signaling ameliorates NLRP3-related pyroptosis in DOCA-salt mice

We next treated DOCA-salt mice with an ATP hydrolase apyrase and a P2X7-specific inhibitor AZ10606120. Preliminary data revealed that neither apyrase nor AZ10606120 had any effect on blood pressure, yet both exerted a certain degree of renoprotective effect (sFig. 3A, B). Both treatments reduced the UACR (sFig. 4A). Histological analysis revealed attenuated tubular injury and tubulointerstitial fibrosis (sFig. 4B–D), accompanied by reduced renal infiltration of P2X7-positive macrophages (sFig. 4B, E) after apyrase or AZ10606120 treatment. Reduced expression of fibrosis and EMT-related genes (*Fn*, *Vim*) as well as inflammatory genes (*Ccl2*, *Il6*) (sFig. 4F), along with reduced levels of NLRP3-related pyroptosis proteins (NLRP3, c-Casp-1, GSDMD-N, and mature IL-1β) were observed after apyrase or AZ10606120 treatment (sFig. 4G, H). Treatment with apyrase or AZ10606120 also inhibited M1 macrophage polarization, as indicated by reduced *Cd86* mRNA levels, while no change in the M2 marker CD206 (*Mrc1*) was observed (sFig. 4I).

### Aldo/MR activation induces PANX1-dependent ATP release in TECs

**Fig. 5:**
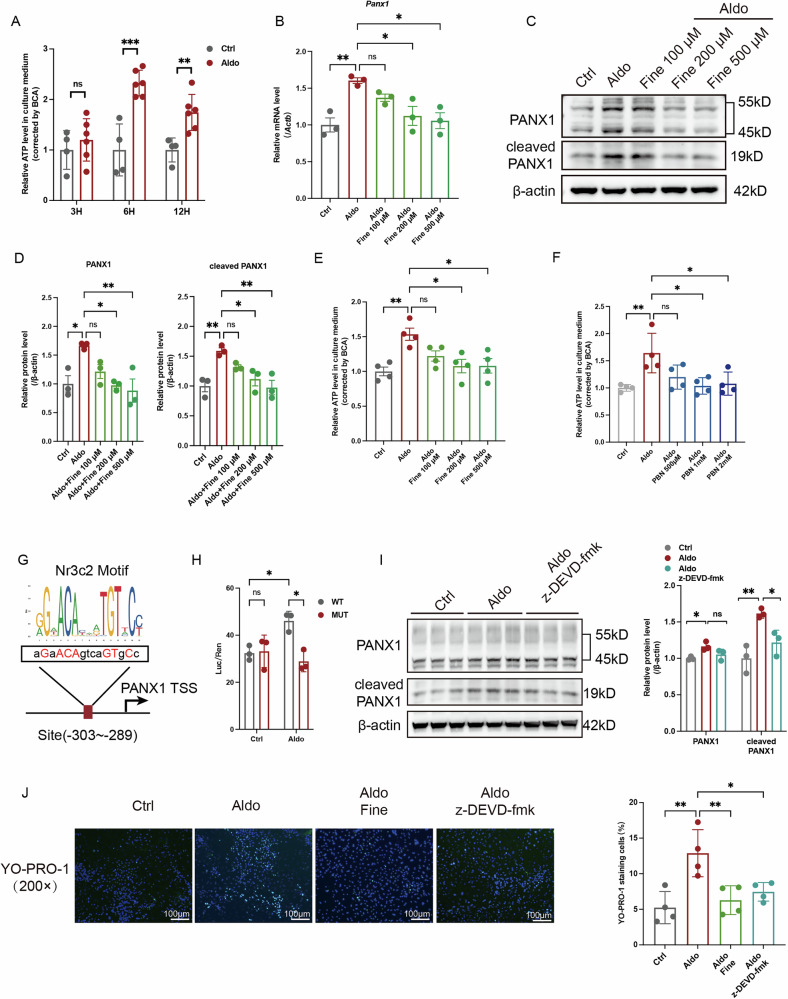
Aldo/MR activation upregulates PANX1 in mTECs following ATP release. **A** ATP levels in the culture medium of mTECs after Aldo stimulation (100 nM); *n* = 4–6 per group by an unpaired *t*-test. **B** *Panx1* mRNA levels in mTECs after stimulation with Aldo at 100 nM for 3 h with or without finerenone (Fine, 100, 200, or 500 µM); *n* = 3 per group; one-way ANOVA with Tukey’s test for multiple comparisons. **C** Representative western blotting images and **D** summary data showing the effects of Aldo (100 nM, 6 h) and Fine (100, 200, 500 µM) on the expression of total and cleaved PANX1 in mTECs for 6 h; *n* = 3 per group; one-way ANOVA with Tukey’s test for multiple comparisons. **E** Finerenone treatment (100, 200, and 500 µM) reduced the ATP concentration in the culture medium of mTECs after Aldo stimulation (100 nM, 6 h); *n* = 4 per group; one-way ANOVA with Tukey’s test for multiple comparisons. **F** Changes in ATP levels in the culture medium of mTECs after Aldo stimulation (100 nM, 6 h) and probenecid (PBN, 0.5, 1, and 2 mM) treatment; *n* = 4 per group; one-way ANOVA with Tukey’s test for multiple comparisons. **G** Bioinformatics analysis revealed the putative *Nr3c2* motif in the upstream promoter region of PANX1. **H** The Luc/Ren ratio in HK2 cells transfected with the different plasmids under Aldo stimulation at 100 nM for 12 h; WT: wild-type vector with the whole *Panx1* translational start site sequence; MUT: vector with a mutation in the binding site; *n* = 3 per group; two-way ANOVA with Tukey’s test for multiple comparisons. **I** Representative western blotting images and summary data showing the effect of z-DEVD-fmk (100 µM) on PANX1 expression under Aldo stimulation (100 nM, 6 h); *n* = 3 per group; one-way ANOVA with Tukey’s test for multiple comparisons. **J** Representative images and summary data showing the effects of finerenone (200 µM) and z-DEVD-fmk treatment (100 µM) on the percentage of YO-PRO-1-positive mTECs; magnification: 200×; scale bar: 100 µm; *n* = 4 per group; one-way ANOVA with Tukey’s test for multiple comparisons. ^ns^*p* > 0.05, *p < 0.05, **p < 0.01, and ***p < 0.001.

The ATP levels in the conditioned medium of mTECs with Aldo treatment were measured. Aldo induced time-dependent ATP release by mTECs. The ATP level in the medium peaked at 6 h (Fig. 5A). Interestingly, the PANX1 mRNA level began to increase at 3 h after Aldo treatment (Fig. 5B). At 6 h after Aldo treatment, both total and activated PANX1 protein levels increased in mTECs (Fig. 5C, D). The CCK-8 results revealed that finerenone (0.1–1 mM) had no cytotoxic effect on mTECs (sFig. 5A). Importantly, finerenone abrogated the upregulation of PANX1 expression at both the mRNA and protein levels (Fig. 5B–D) and suppressed Aldo-induced ATP release (Fig. 5E) in a dose-dependent manner. Notably, PBN, which had no cytotoxic effect on mTECs at concentrations of 0.5–2 mM (sFig. 5B), simulated the effect of finerenone on Aldo-stimulated ATP release (Fig. 5F), suggesting that PANX1 plays a role in this process.

### Aldo/MR activation promotes PANX1 transcription and activity in TECs

We next explored the underlying mechanism between Aldo/MR and PANX1 upregulation. A putative MR binding site was identified in the PANX1 promoter region via the JASPAR database (Fig. 5G). A dual-luciferase reporter assay revealed that after treatment with Aldo, the luciferase fluorescence intensity of the WT PANX1 group increased by 42.41%, while the luciferase fluorescence intensity of the MUT PANX1 group largely decreased compared with that of the WT PANX1 group (Fig. 5H).

In addition to promoting gene transcription, Aldo/MR is known to induce reactive oxygen species (ROS) -dependent Caspase-3 activation [32, 33]. This process may mainly depend on an MR-sensitive gene NOX4, in TECs [10]. Significantly, activated Caspase-3 has been reported to cleave PANX1, resulting in a constitutively open channel [34]. Therefore, we detected the levels of NOX4 and ROS (presented as DCFH-DA fluorescence) in mTECs under Aldo stress. Notably, Aldo significantly increased NOX4 protein (sFig. 6A), DCFH-DA fluorescence (sFig. 6B), c-Casp-3 protein (sFig. 6A) and Caspase-3 enzymatic activity (sFig. 6C) in mTECs. These effects were attenuated by finerenone treatment (sFig. 6A–C). The Caspase 3 inhibitor z-DEVD-fmk was further used to verify the role of c-Casp-3 in activation of PANX1. As shown in Fig. 5I, z-DEVD-fmk did not affect the total PANX1 protein level but did reduce the level of cleaved PANX1. Activation of PANX1 allowed the entry of the fluorescent dye YO-PRO-1 [28, 34]. To verify the activity of the PANX1 channel directly, we treated mTECs with the dye YO-PRO-1. As shown in sFig. 6D, Aldo increased the percentage of YO-PRO-1-positive cells, but this effect was effectively inhibited by the PANX1 inhibitor PBN. Moreover, the increasing trend was offset by both the finerenone treatment and the z-DEVD-fmk treatment (Fig. 5J).

In summary, these results reveal that Aldo/MR activated PANX1 in two ways: promoting PANX1 transcription and increasing PANX1 activity via NOX4/ROS/c-Casp-3 signaling in TECs.

### TEC‒macrophage crosstalk facilitates Aldo/MR-induced macrophage pyroptosis and migration

**Fig. 6:**
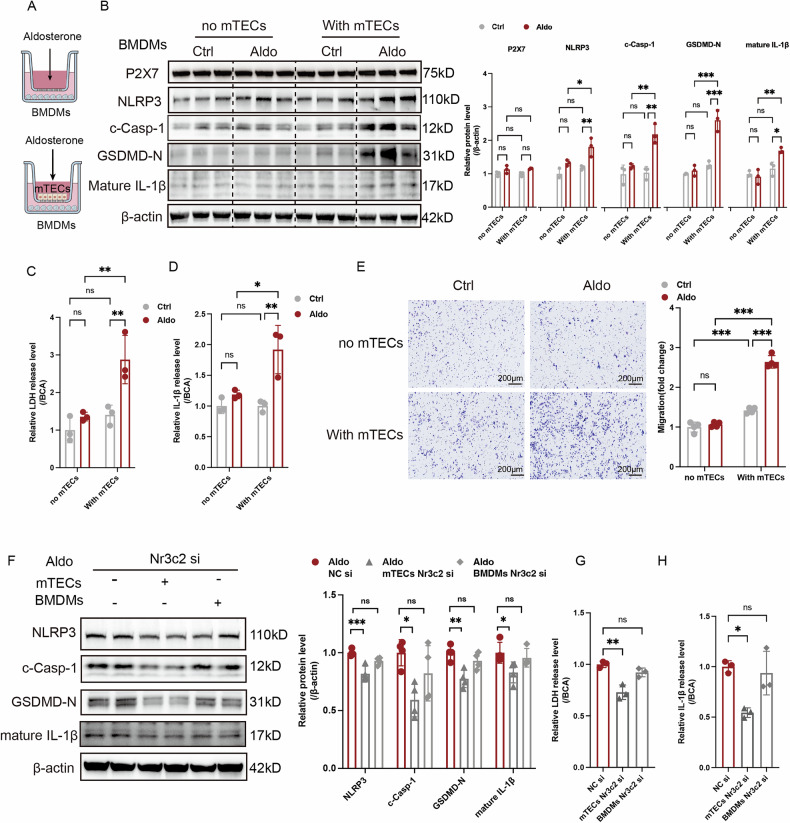
Aldo induces macrophage pyroptosis and migration in an mTEC-dependent manner. **A** Schematic diagram of the coculture experiment. **B** Representative western blotting images and summary data showing the expression levels of P2X7 and pyroptosis-related proteins, including NLRP3, cleaved Caspase-1 (c-Casp-1), GSDMD N-terminal, and mature IL-1β, in BMDMs singly cultured or cocultured with mTECs under Aldo stimulation (100 nM, 6 h); *n* = 3 per group; two-way ANOVA with Tukey’s test for multiple comparisons. **C** Relative LDH release levels and **D** IL-1β levels in the culture supernatants of BMDMs singly cultured or cocultured with mTECs under Aldo stimulation (100 nM, 6 h); *n* = 3 per group; two-way ANOVA with Tukey’s test for multiple comparisons. **E** Representative images (magnification: 100×; scale bar: 200 µm) and summarized data showing the migration of BMDM single culture or coculture with mTECs under Aldo stimulation (100 nM, 24 h); *n* = 4 per group; two-way ANOVA with Tukey’s test for multiple comparisons. **F** Representative images and summary data showing the effect of *Nr3C2* siRNA in mTECs or BMDMs on BMDM pyroptosis in the coculture system under Aldo stimulation (100 nM, 6 h); *n* = 4 per group; one-way ANOVA with Tukey’s test for multiple comparisons. **G** Relative LDH release levels and **H** IL-1β levels in BMDM culture supernatants from coculture experiments, showing the effects of MR knockdown in mTECs or BMDMs after Aldo stimulation (100 nM, 6 h); *n* = 3 per group; one-way ANOVA with Tukey’s test for multiple comparisons. ^ns^*p* > 0.05, *p < 0.05, **p < 0.01, and ***p < 0.001.

We next analyzed the effect of TEC‒macrophage crosstalk on macrophage pyroptosis under Aldo stress via coculture experiments. The coculture system is shown in Fig. 6A, and it was cocultured for 6 h. As shown in Fig. 6B, the expression of P2X7 in BMDMs was not influenced by Aldo treatment or coculture with TECs, separately or simultaneously. Aldo alone minimally affected NLRP3-induced pyroptosis signaling in BMDMs. However, when cocultured with mTECs, Aldo induced notable pyroptosis in BMDMs, as indicated by significantly increased levels of NLRP3, c-Casp-1, GSDMD-N, and mature IL-1β (Fig. 6B). Similarly, LDH and IL-1β release from BMDMs did not change upon Aldo stimulation alone or when the cells were cocultured with TECs but significantly increased when the BMDMs were cocultured with mTECs and exposed to Aldo stimulation (Fig. 6C, D). To examine changes in macrophage migration, mTEC-BMDMs were cocultured for 24 h under Aldo stress. Aldo alone did not affect BMDM migration. The migration of BMDMs slightly increased when they were cocultured with mTECs. Notably, when Aldo was added to the coculture system, BMDM migration significantly increased (Fig. 6E).

MR is shown to be expressed in mTECs and BMDMs, though its abundance in mTECs was more than twofold greater than that in BMDMs (sFig. 7A) in basal medium. To evaluate the contributions of MR of two cell types, *Nr3c2* siRNA was used to knockdown MR in mTECs or BMDMs (sFig. 7B) under Aldo stress, respectively. MR knockdown in mTECs significantly attenuated BMDM pyroptosis, LDH and IL-1β release and migration in the coculture system, whereas MR knockdown in BMDMs resulted in little change in BMDM pyroptosis and migration (Fig. 6F–H and sFig. 7C).

These results show that MR activation in TECs is more important than that in macrophages in renal environment.

### PANX1-eATP-P2X7 signaling mediates tubular macrophage crosstalk under Aldo stress

**Fig. 7:**
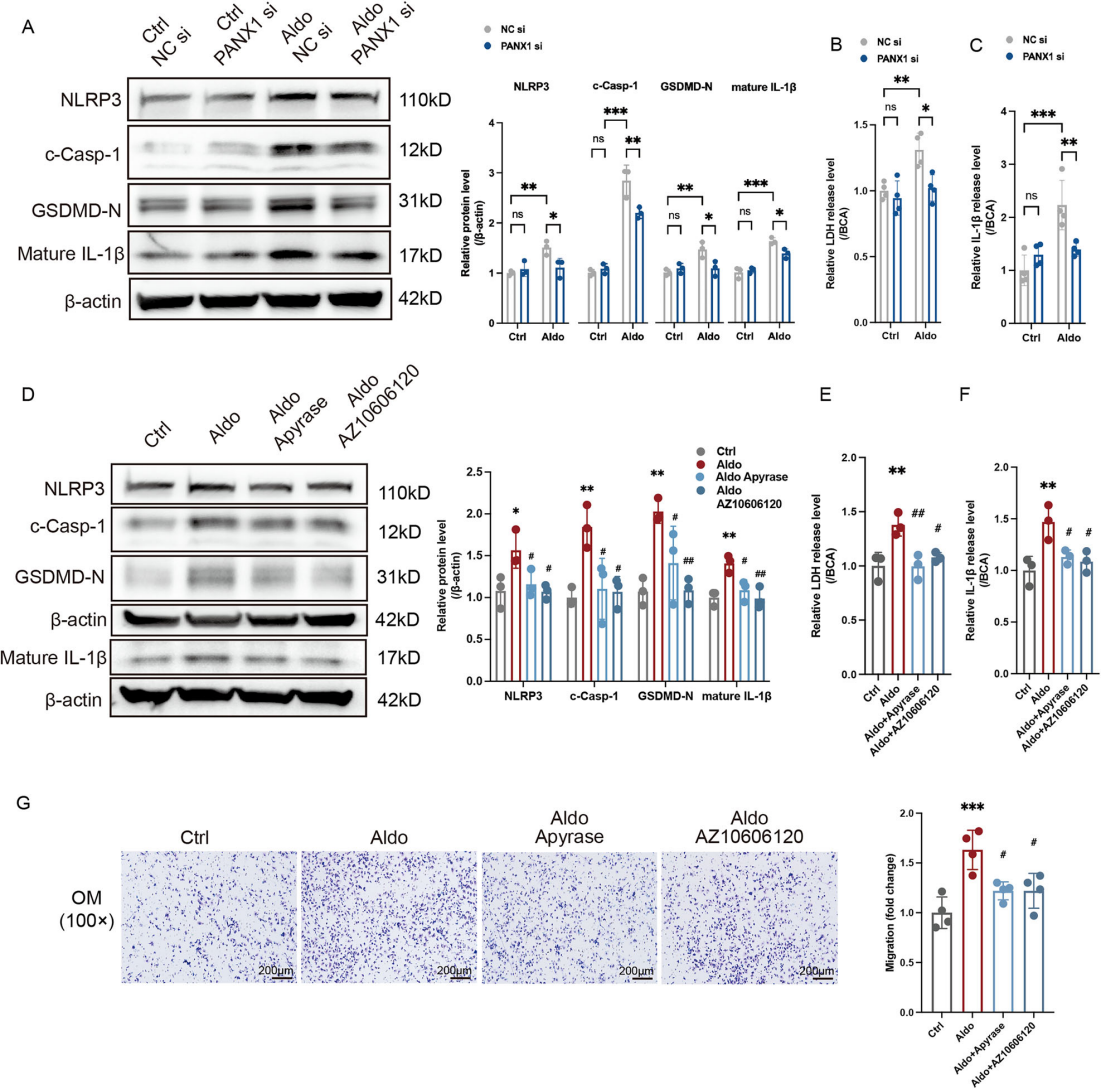
PANX1-mediated ATP release from mTECs is responsible for macrophage pyroptosis and migration under Aldo stimulation. **A** Representative western blotting images and summary data showing the expression of the pyroptosis-related proteins NLRP3, cleaved Caspase-1 (c-Casp-1), GSDMD N-terminal, and mature IL-1β in BMDMs cocultured with mTECs that had been treated with NC siRNA or *Panx1* siRNA under Aldo stimulation (100 nM, 6 h); *n* = 3 per group. ^ns^*p* > 0.05, *p < 0.05, **p < 0.01, and ***p < 0.001; two-way ANOVA with Tukey’s test for multiple comparisons. **B** Relative LDH release levels and **C** IL-1β levels in the culture supernatants of BMDMs cocultured with mTECs that had been treated with NC siRNA or *Panx1* siRNA under Aldo stimulation (100 nM, 6 h); *n* = 4 per group. ^ns^*p* > 0.05, *p < 0.05, **p < 0.01, and ***p < 0.001; two-way ANOVA with Tukey’s test for multiple comparisons. **D** Representative western blotting images and summary data showing the expression levels of pyroptosis-related proteins in BMDMs cocultured with mTECs under Aldo stimulation (100 nM, 6 h) with apyrase (0.5 U/mL) or AZ10606120 (50 µM) treatment; *n* = 3 per group; one-way ANOVA with Tukey’s test for multiple comparisons. **E** Relative LDH release levels and **F** IL-1β levels in the culture supernatants of BMDMs cocultured with mTECs under Aldo stimulation (100 nM, 6 h) with apyrase (0.5 U/mL) or AZ10606120 (50 µM) treatment; *n* = 3 per group; one-way ANOVA with Tukey’s test for multiple comparisons. **G** Representative images (magnification: 100×; scale bar: 200 µm) and summarized data showing the migration of BMDMs cocultured with mTECs under Aldo stimulation (100 nM, 24 h) and apyrase or AZ10606120 treatment; *n* = 4 per group; one-way ANOVA with Tukey’s test for multiple comparisons. ^ns^*p* > 0.05, *p < 0.05, **p < 0.01, and ***p < 0.001 vs. the Ctrl group; ^ns^*p* > 0.05, ^#^*p* < 0.05, ^##^*p* < 0.01, and ^###^*p* < 0.001 vs. the Aldo group.

*Panx1* siRNA was used to knock down PANX1 expression in mTECs for further validation (sFig. 8A). The results revealed that *Panx1* siRNA significantly reduced NLRP3-related pyroptosis (Fig. 7A), LDH and IL-1β release (Fig. 7B, C), and the migration of BMDMs (sFig. 8B) under Aldo-induced stress. To determine the contribution of eATP-P2X7 signaling to this process, apyrase and AZ10606120 were used. The results of the CCK8 assay revealed that apyrase (0.5 U/mL) was not cytotoxic to either mTECs or BMDMs and could effectively eliminate ATP released from mTECs after Aldo stimulation (sFig. 9A, B). AZ10606120 (50 μM) also had no cytotoxic effects on mTECs or BMDMs (sFig. 9A). Importantly, the addition of both apyrase and AZ10606120 to the coculture system reversed NLRP3-related pyroptosis activation in BMDMs (Fig. 7D), reduced the release of LDH and IL-1β from BMDMs (Fig. 7E, F), and inhibited BMDM migration (Fig. 7G).

In summary, our in vitro experiments revealed the detailed mechanism of PANX1-eATP-P2X7 signaling in TEC–macrophage crosstalk under Aldo/MR activation.

### scRNA-seq data reveal P2X7/NLRP3-related pyroptosis pathway activation in macrophages of DOCA-salt mice

**Fig. 8:**
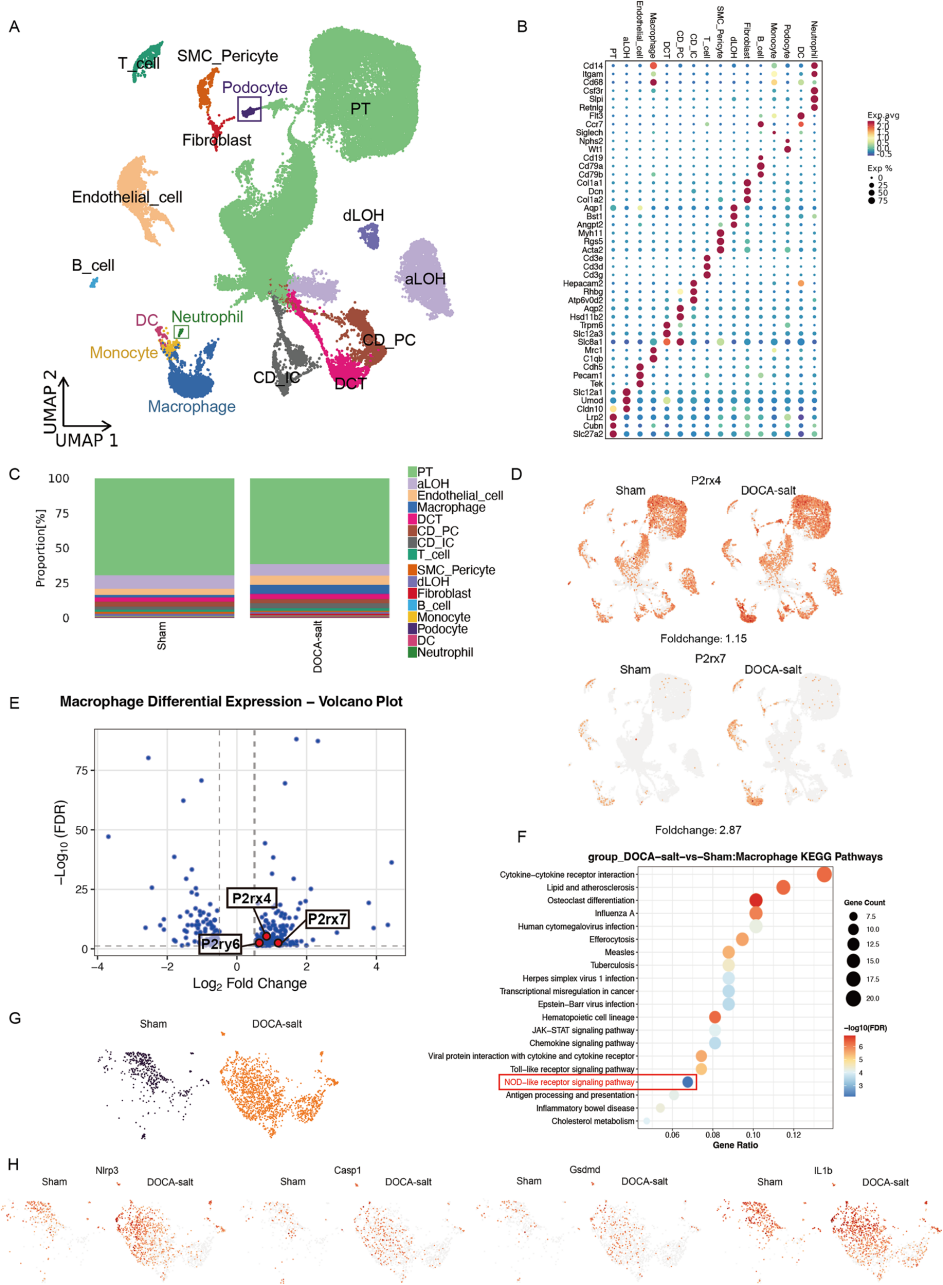
scRNA-seq analysis of kidney samples from sham mice and DOCA-salt mice. **A** UMAP plot showing 16 clusters identified in mouse samples using scRNA-seq, including proximal tubular cells (PT), distal convoluted tubular cells (DCT), collecting duct principal cells (CD_PC), collecting duct intercalated cells (CD_IC), epithelial cells in a thick ascending limb of the loop of Henle (aLOH), epithelial cells in a descending limb of the loop of Henle(dLOH), endothelial cells, podocytes, smooth muscle cells/pericytes (SMC_Pericyte), fibroblasts, monocytes, macrophages, neutrophils, B cells, T cells, and dendritic cells (DC). **B** Dot plot of cell-type marker genes. The color of dots represents average expression, and the size of dots represents the average percentage of cells expressing the selected genes. **C** Percentage of cells in each cluster. **D** UMAP plot showing the expression of *P2rx4* and *P2rx7* in sham mice and DOCA-salt mice. **E** Volcano plot of DEGs in macrophages from sham mice and DOCA-salt mice. **F** KEGG analysis of DEGs in macrophages from sham mice and DOCA-salt mice. **G** UMAP plot of macrophages in the sham and DOCA-salt mice. **H** Expression of Nlrp3, Casp1, Gsdmd, and Il1b in the above groups. *n* = 3 per group.

scRNA-seq data from kidney samples of sham mice and DOCA-salt mice were analyzed. A total of 16 cell clusters were identified (Fig. 8A) based on cell type-specific markers (Fig. 8B). Among all cell clusters, macrophage showed the most significant change in proportion after DOCA-salt treatment (Fig. 8C and sFig. 10A). The expression of two major eATP–driven signals, namely, purinergic and adenosine receptors, was analyzed. 6 purinergic genes (*P2rx5, P2rx7, P2ry6, P2ry10b, P2ry12, P2ry13*) and 2 adenosine receptors (*Adora2a and Adora2b*) were upregulated in the DOCA-salt mice (*p* < 0.1; Fig. 8D and sFig. 10B–I). Among the purinergic and adenosine receptors, only *P2rx4, P2rx7*, and *P2ry6* were specifically increased (*p* < 0.05) in macrophages (Fig. 8E). P2X4 and P2X7 are activated by ATP, while P2Y6 is activated by UTP. Notably, P2X7 was enriched in macrophages, while P2X4 was almost expressed in all clusters (Fig. 8D). Furthermore, KEGG analysis of macrophage DEGs demonstrated significant involvement of the NOD-like receptor signaling pathway (NLRP3 inflammasome axis) in DOCA-salt mice (Fig. 8F). A UMAP plot of macrophages is presented in Fig. 8G, and further analysis of changes in NLRP3 inflammasome-related gene expression is presented in Fig. 8H. The proportions of *Nlrp3, Casp1, Gsdmd, and Il1b* in macrophages increased within the kidneys of DOCA-salt mice (Fig. 8H), further supporting the specific involvement of the NLRP3 inflammasome in macrophages in Aldo-dependent renal injury.

## Discussion

In this study, we demonstrated the critical role of tubular–macrophage crosstalk in Aldo/MR-mediated hypertensive renal injury. Our results revealed that eATP played an important role in the early crosstalk between TECs and macrophages under Aldo stress. Specifically, Aldo/MR activation first promoted PANX1 transcription and increased PANX1 activity via NOX4/ROS/c-Casp-3 signaling to increase ATP release by TECs. Increased eATP in the tubulointerstitium recruited macrophages and triggered P2X7-NLRP3 signaling to facilitate macrophage pyroptosis. PANX1 knockdown or treatment with finerenone or PANX1-eATP-P2X7 signaling inhibitors protected against Aldo-induced hypertensive renal injury by mitigating macrophage migration and pyroptosis.

Aldo is a steroid hormone that affects physiopathological processes when it binds to MR. The classical mechanism of Aldo/MR activation involves the regulation of sodium channel expression in the Aldo-sensitive distal nephron (ASDN) [7, 8], leading to sodium and water retention and hypertension aggravation. Furthermore, Aldo/MR activation induces oxidative stress injury in renal intrinsic cells, including TECs, podocytes [35], and mesangial cells [36], to promote renal injury and interstitial fibrosis. Studies suggest that Aldo/MR increases NOX4-derived ROS production in TECs [9, 10], resulting in metabolic dysregulation [37], apoptosis [38], and mesenchymal transition [37, 39]. Therefore, targeting MR not only benefits blood pressure management but also provides a direct renoprotective effect. In the current study, a low dose of finerenone significantly protected against Aldo/MR-induced renal injury, which is independent of blood pressure. Notably, MR activation in TECs resulted in an adverse phenotype, whereas MR activation in macrophages had little effect. Therefore, we conclude that in addition to aggravating hypertension, TECs are the main target of Aldo/MR to induce direct renal injury. However, MR is highly expressed in the ASDN, we did not further separate ASDN since positive results have been obtained. Further experiments are needed to comprehensively elucidate the underlying mechanisms.

Extracellular ATP is recognized as an early paracrine molecule released from stressed or injured cells in inflammatory diseases [40, 41]. Plasma ATP levels increase as early as approximately one week after onset in both angiotensin II-induced and L-NAME-induced hypertension models [16]. In mice with I/R-induced AKI, plasma ATP levels peak at 4 h after reperfusion [17]. Our results further confirm that urinary ATP is an early indicator of Aldo-induced hypertensive renal tubular injury. eATP has been reported to act as an intermediate factor in TEC‒macrophage crosstalk to promote renal injury in AKI and CKD [19]. Our results provide novel evidence to support this role in Aldo-induced hypertension.

The pannexin family is a cluster of membrane channels that release intracellular ATP. Among these, PANX1 is the most abundant subtype. In the kidney, PANX1 is expressed in the apical and basolateral membranes of the proximal tubules and the ASDN [42, 43]. Growing evidence indicates that PANX1-mediated ATP release contributes to the progression of kidney diseases via cell-to-cell communication [44–46]. In angiotensin II-induced hypertension, PANX1-induced ATP autocrine results in mesangial dysfunction [46]. A previous study reported that the MR antagonist spironolactone inhibits PANX1 activity, but is independent of MR blockade [47]. The current study further provides critical data indicating that PANX1 is responsible for Aldo/MR-induced tubular ATP release. Moreover, our study is the first to report that Aldo/MR promotes PANX1 transcription in addition to activating NOX4/ROS/c-Casp-3 signaling. Once ATP efflux begins, eATP can induce additional ATP release via P2 receptors [48, 49]. In addition, macrophage pyroptosis leads to massive ATP leakage. Overall, Aldo/MR initiates eATP efflux, and a high eATP microenvironment is generated via the amplification processes above.

In the tubulointerstitium, eATP functions through a family of P2 receptors at early stage [50, 51]. Among these, P2X7 and P2X4 are two major types of ATP-specific P2 receptors [16, 27]. The present study systematically investigates the distribution and expression of different P2 receptor subtypes via scRNA-seq analysis. The results verified that P2X7 and P2X4 are significantly upregulated in macrophages from DOCA-salt mice. In a high-eATP microenvironment, both P2X4 and P2X7 can be activated. Compared with P2X4, P2X7 has a stronger effect on inflammation because of its greater resistance to desensitization [52]. Exposure to eATP rapidly induces the internalization of P2X4, while P2X7 remains active in the cytomembrane. The binding of P2X7 with eATP results in K^+^ efflux, leading to NLRP3 inflammasome activation and subsequent pyroptosis [53, 54]. Interestingly, Aldo/MR has been reported to promote NLRP3 transcription by facilitating nuclear translocation of NK-κB in macrophages [55]. Our results provide further evidence that Aldo/MR triggers macrophage pyroptosis via eATP-mediated crosstalk between TECs and macrophages. Given that purinergic signaling may shift towards adenosine-mediated pathways over time, we also analyzed the expression of P1 adenosine receptors. Among P1 receptors, Adora2a and Adora2b were increased in the kidneys of DOCA-salt mice, albeit at a modest level, indicating that adenosine‑mediated pathways were also slightly activated. Notably, the expression of none of these P1 receptors was increased in macrophages, and increased expression of Adora2a was detected mainly in endothelial cells, whereas Adora2b expression was detected mainly in tubular cells. At present, no data are available concerning the effect of adenosine-mediated signaling in Aldo-dependent hypertension. Targeting Adora2a and Adora2b appears to have opposite effects on different hypertensive backgrounds [56–58]. Therefore, we speculate that P2X7-driven macrophage pyroptosis is the dominant eATP-driven mechanism in DOCA-salt-treated mice, and future work is needed to elucidate the roles of adenosine-mediated signaling in Aldo-dependent hypertension.

Both the pyroptosis and the polarization of macrophages are critical for renal fibrosis in DOCA-salt-induced renal injury. In the current study, we revealed that macrophage pyroptosis, as well as the infiltration of both M1-like and M2-like macrophages, increased in the kidneys under Aldo-dependent hypertensive injury; however, targeting Aldo/MR-PANX1-P2X7 signaling significantly mitigated macrophage pyroptosis and the infiltration of M1-like but not M2-like macrophages, suggesting that the inflammatory response triggered by macrophage pyroptosis and M1-like macrophages contributes more than M2-like macrophages to promoting renal fibrosis in Aldo-dependent hypertensive renal injury. Similar roles of macrophage pyroptosis and M1-like macrophages in promoting renal fibrosis have been reported previously. For example, the MR antagonist spironolactone preferentially inhibited M1-like macrophages to attenuate renal fibrosis in DOCA-salt-treated mice [59], whereas genetic deletion of one of the NLRP3 inflammasome components mitigated DOCA-salt-induced renal injury [60]. Our results provide further evidence that macrophage pyroptosis participates in the infiltration of M1-like macrophages to contribute to renal fibrosis in Aldo-dependent hypertensive renal injury.

In summary, the results of the current study revealed that Aldo/MR drives PANX1-eATP-P2X7 signaling to induce macrophage pyroptosis via TEC–macrophage crosstalk in DOCA-salt-induced hypertensive renal injury. Our study provides a new perspective on the pathophysiology of hypertensive renal injury and new targets, including PANX1, eATP, and P2X7, for the treatment of hypertensive individuals with high levels of Aldo. Finerenone, PBN, apyrase, and AZ10606120 are prominent drugs used for blocking the early progression of hypertensive renal injury.

There are several limitations in the current study. First, we only conducted experiments in a DOCA-salt mouse model. DOCA is a precursor of Aldo. Although the effect of DOCA is similar to that of Aldo, the DOCA-salt mouse model may not fully simulate the pathological mechanisms of high Aldo-dependent renal injury in vivo. Future confirmation is suggested in Aldo synthase transgenic mice with high levels of endogenous Aldo. Additionally, we did not explore the effect of high Aldo in other hypertension models, such as angiotensin II-infusion mice or spontaneously hypertensive rats, considering that Aldo/MR is indirectly activated by angiotensin II or genetic background and may be unstable. Further studies are needed to elucidate the universal role of PANX1-eATP-P2X7 signaling in these models. Finally, although we found Caspase-3 served as an upstream driver of PANX1 cleavage and P2X7-driven pyroptosis in macrophages, a limited increase in TEC apoptosis was observed in DOCA-salt-treated mice. It is difficult to totally preclude the potential contributions of Caspase-3 triggered TEC apoptosis in Aldo-dependent renal injury by existing methods, on account that no methods can inhibit apoptosis without affecting Caspase-3 activity. In the future, novel apoptosis inhibitors targeting downstream Caspase-3 may help solve this issue.

## Supplementary information

Supplementary Figure Legends

sFigure 1

sFigure 2

sFigure 3

sFigure 4

sFigure 5

sFigure 6

sFigure 7

sFigure 8

sFigure 9

sFigure 10

Supplementary materials

Original western blots

## Data Availability

The single-cell RNA sequencing data have been deposited in the NCBI Gene Expression Omnibus (GEO) database under accession number GSE328129. Other datasets used and/or analyzed in the current study are available from the corresponding author on reasonable request.
