# Supplementary material for: Tubular epithelial cells promote macrophage pyroptosis through PANX1-extracellular ATP-P2X7 signaling in aldosterone-dependent renal injury
Source: Cell Death Dis. 2026 Jun 5;17(1):682. doi: 10.1038/s41419-026-08951-3 (PMC13438567; doi:10.1038/s41419-026-08951-3)
Supplement: Supplementary file 1 [file 41419_2026_8951_MOESM12_ESM.pdf]

Supplemental materials

Sequences of the Panx1 promoter luciferase reporter plasmids

Wild-type (the predicted site has been highlighted with an underline):

CAAAGGGACCTAATCTAGTCTGTCTACTTAGGGAAGGCTTTCCCAGGGAA
GTGACATCAAGCTGAGGCCAGAAGGGTGAATAAAGGTGGGCCAGGCAAA
GAGGCCACTGTGGGGAGTGTTTTGGGTGGGAAGGCCTGGAGGAGGCCAA
GGGCTTAGCTAACTTGAAGAACTGAAAGGAAAGCAGTGAAGCTGGTGGT
GAGGTAGGCAGAGGCCATGCTAAGAAATTTGTATTTTATGCTAAAAACAA
TGTACACTTTAAGTAGAAAAGATGATCCGACTGGAGTTTTGAAAAGCTCA
TCCTGGCAGCTGTAGAGGAGAATGGAGGGGGACCCTGTCTCACATATTTC
CTATATGTGGTTAGGAGACAGGATGGTGAGTTGAATTGGGTATTGGCAGT
GCAGGTGGAGGGGTGTCAGATTTGCTATATTTAGGAGAGAGAAGCAAGA
GAATTTGAAAATTGGTTATGGCAGCTGTGAGAAATGGAGTGTTGAAGATG
GTGCTCCAGGATTCCGACTTGGGTGCACAGGTGGCTGGTGGTACATTTAG
TAAGATGGGGAAGATGGAGTTCAGGTTTAAGGGGAAGATGAAGACTGAA
GTCCTGGCCGTTATGAGTTTGAAGCCCTATGAGCCATCTACGCGGAGAGA
GCTGTATACGCTCTTGGATGTGTGTTTAGCGGCCAGGCTGGGGTGGAGAG
GTACATGTGGGAGTTGAGGCTTGTATCTGTGGACCCACAGAGAAGGGAGC
CAAGCCCTGCCCCAAGGAGCCCTCATGTTTGCAGGTCCTGCCTGCTTCAAA
TGTTACTGGTGATAACCTACATGTCACATTTGACAGGGCTTCCAAACTAAG
CCATCTAAGAAAGGTCTTATGATTCATGGCTTAGATCCTGTCCCTGAGTAA
AAAATCTTACTGTGAGTCCTTCAAATCCTTTTGTCAGTTCCTCAAACTGCT

GATGTACTGATTAGTATGTAAAATACTGACATTGAAAAGAACACTGACTTGTTTCTGAATCATATAAACTTTGATTTTCTTGTGCACAAAACATTTTAGCCTGTATGTTGTCATCTGCAGCCAGTGACTGTAACCTGTGTACCATACCCTCCGATGAAAAAAATCCCACCTCTGATATAAGGGGTCCTCCTCCCTTTTCCTAAACTTTCTTCTAAAACCCTTCCAACGTGTAACAAATTTTGAAACATGCCCAACTTTGCTGGTAAGTCTTCTTGGGTGGATCCTCACATTTAGCTTCCAATAAATCAAATTATTTCTACTTCAACAGCCTTAATTTCCGTGGACACTGGTCAGAGTATTTCCTCCTGTCTTTCCATCATCCACTTCCTATGAGCTGTTCAGAAGCAGCTCTCTGCAGCCCCAACACCCACCTACCTGCAGGCCCCCCTCTCTCTGCTGGCTCCCGGAACAAGGCTCTGATTGGGATGGCAGAGGAAAGAGAAACGGGACCAGCAGCGCTACTCAGGCCTCGAAACTCCACACTCACTACCGTTTCCGCGCCACCCTCTCACGCGGAGCTCCTGGAAGGGTCCAGCGCTCCCTCTGCAAGGCACCGTCGGTCTACACCGCTCGATTGGCCTTTTATGGCACTGACTGTTCTTTGAAGCTTTTGCAGCTCCTTCTAACCCGATTTCACCGTGTTGGAAATGTTGGGGGTGGGGCGTAAAGGGGGCCATCCCATCCACAGAAAGAGTCGCTTGCAGAAATGATTTTAAAACTCCCGGGTGCCAGGACGTGAGGAGAATCCGGCAGGGAGGGACGGGAGAATTTCCAAAGGTGCCGCTCAGTGGGAAAATCCCTTTGTCAGAGAGTGGCCCGAGGGGCGCGGAATGCAGCCGCGCCCACCCCGCCCCGCGTCACCGCGTCTTCCGGAAGCTCCACGCCCCTGGGTACTTGGTTTCCCCGCATGGTTCCGGAAGAGCGCGGCGCAGCTGGCTGTGAGCGCAGGGCTATCCCGGCGGCCGCTTCGGCAGCCAGGGCGGCGCGGAGG

NR3C2-binding-site mutant:

CAAAGGGACCTAATCTAGTCTGTCTACTTAGGGAAGGCTTTCCCAGGGAA
GTGACATCAAGCTGAGGCCAGAAGGGTGAATAAAGGTGGGCCAGGCAAA
GAGGCCACTGTGGGGAGTGTTTTGGGTGGGAAGGCCTGGAGGAGGCCAA
GGGCTTAGCTAACTTGAAGAACTGAAAGGAAAGCAGTGAAGCTGGTGGT
GAGGTAGGCAGAGGCCATGCTAAGAAATTTGTATTTTATGCTAAAAACAA
TGTACACTTTAAGTAGAAAAGATGATCCGACTGGAGTTTTGAAAAGCTCA
TCCTGGCAGCTGTAGAGGAGAATGGAGGGGGACCCTGTCTCACATATTTC
CTATATGTGGTTAGGAGACAGGATGGTGAGTTGAATTGGGTATTGGCAGT
GCAGGTGGAGGGGTGTCAGATTTGCTATATTTAGGAGAGAGAAGCAAGA
GAATTTGAAAATTGGTTATGGCAGCTGTGAGAAATGGAGTGTTGAAGATG
GTGCTCCAGGATTCCGACTTGGGTGCACAGGTGGCTGGTGGTACATTTAG
TAAGATGGGGAAGATGGAGTTCAGGTTTAAGGGGAAGATGAAGACTGAA
GTCCTGGCCGTTATGAGTTTGAAGCCCTATGAGCCATCTACGCGGAGAGA
GCTGTATACGCTCTTGGATGTGTGTTTAGCGGCCAGGCTGGGGTGGAGAG
GTACATGTGGGAGTTGAGGCTTGTATCTGTGGACCCACAGAGAAGGGAGC
CAAGCCCTGCCCCAAGGAGCCCTCATGTTTGCAGGTCCTGCCTGCTTCAAA
TGTTACTGGTGATAACCTACATGTCACATTTGACAGGGCTTCCAAACTAAG
CCATCTAAGAAAGGTCTTATGATTCATGGCTTAGATCCTGTCCCTGAGTAA
AAAATCTTACTGTGAGTCCTTCAAATCCTTTTGTCAGTTCCTCAAACTGCT
GATGTACTGATTAGTATGTAAAATACTGACATTGAAAAGAACACTGACTT
GTTTCTGAATCATATAAACTTTGATTTTCTTGTGCACAAAACATTTTAGCCT
GTATGTTGTCATCTGCAGCCAGTGACTGTAACCTGTGTACCATACCCTCCG

ATGAAAAAAATCCCACCTCTGATATAAGGGGTCCTCCTCCCTTTTCCTAAACTTTCTTCTAAAACCCTTCCAACGTGTAACAAATTTTGAAACATGCCCAACTTTGCTGGTAAGTCTTCTTGGGTGGATCCTCACATTTAGCTTCCAATAAATCAAATTATTTCTACTTCAACAGCCTTAATTTCCGTGGACACTGGTCAGAGTATTTCCTCCTGTCTTTCCATCATCCACTTCCTATGAGCTGTTCAGAAGCAGCTCTCTGCAGCCCCAACACCCACCTACCTGCAGGCCCCCCTCTCTCTGCTGGCTCCCGGAACAAGGCTCTGATTGGGATGGCAGAGGAAAGAGAAACGGGACCAGCAGCGCTACTCAGGCCTCGAAACTCCACACTCACTACCGTTTCCGCGCCACCCTCTCACGCGGAGCTCCTGGAAGGGTCCAGCGCTCCCTCTGCAAGGCACCGTCGGTCTACACCGCTCGATTGGCCTTTTATGAAGCTTTTGCAGCTCCTTCTAACCCGATTTCACCGTGTTGGAAATGTTGGGGGTGGGGCGTAAAGGGGGCCATCCCATCCACAGAAAGAGTCGCTTGCAGAAATGATTTTAAAACTCCCGGGTGCCAGGACGTGAGGAGAATCCGGCAGGGAGGGACGGGAGAATTTCCAAAGGTGCCGCTCAGTGGGAAAATCCCTTTGTCAGAGAGTGGCCCGAGGGGCGCGGAATGCAGCCGCGCCCACCCCGCCCCGCGTCACCGCGTCTTCCGGAAGCTCCACGCCCCTGGGTACTTGGTTTCCCCGCATGGTTCCGGAAGAGCGCGGCGCAGCTGGCTGTGAGCGCAGGGCTATCCCGGCGGCCGCTTCGGCAGCCAGGGCGGCGCGGAGG
