# Supplementary material for: Tubular epithelial cells promote macrophage pyroptosis through PANX1-extracellular ATP-P2X7 signaling in aldosterone-dependent renal injury
Source: Cell Death Dis. 2026 Jun 5;17(1):682. doi: 10.1038/s41419-026-08951-3 (PMC13438567; doi:10.1038/s41419-026-08951-3)
Supplement: Supplementary file 2 [file 41419_2026_8951_MOESM13_ESM.pdf]

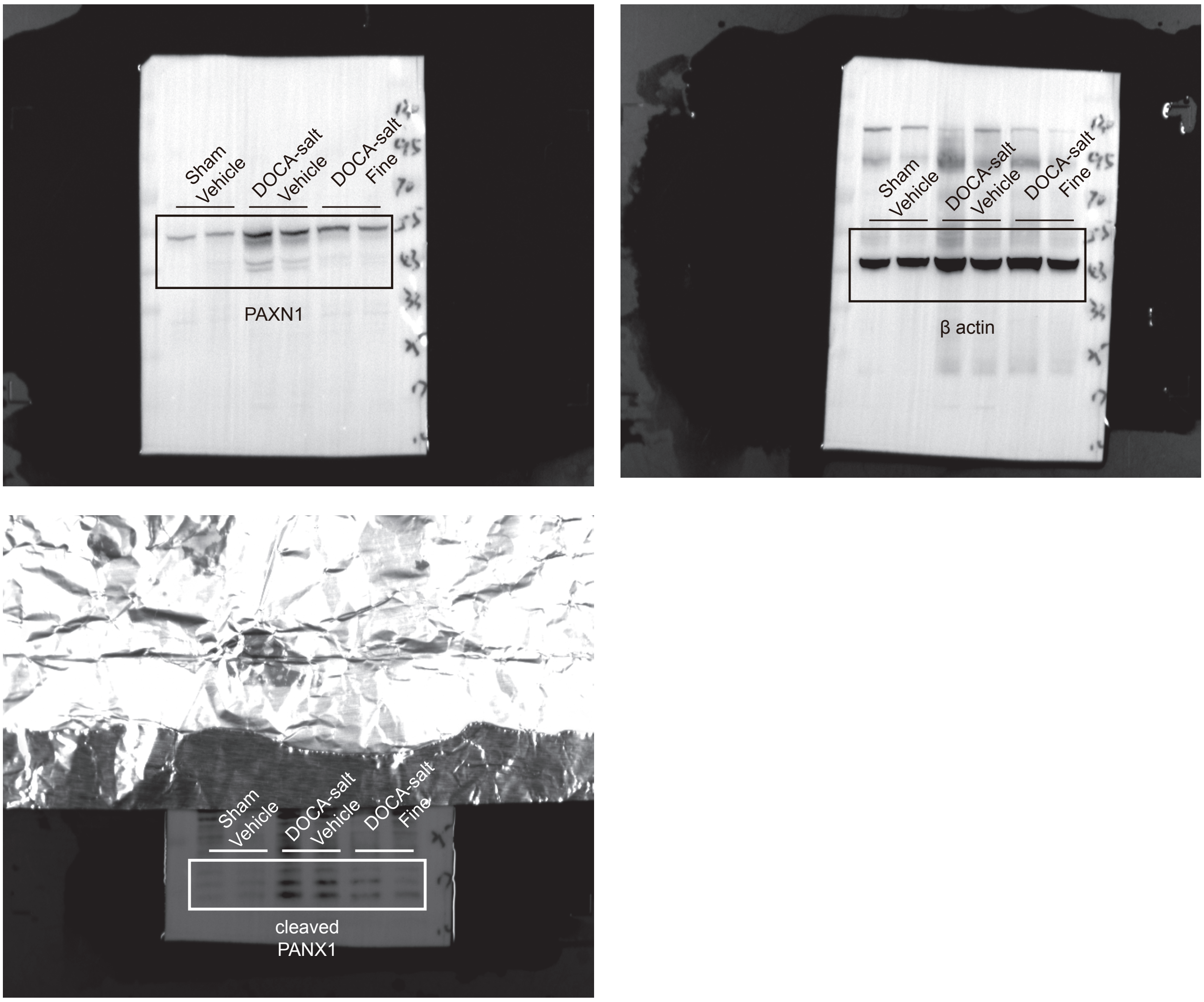
Sham Vehicle
DOCA-salt Vehicle
DOCA-salt Fine
PAXN1
Sham Vehicle
DOCA-salt Vehicle
DOCA-salt Fine
β actin
Sham Vehicle
DOCA-salt Vehicle
DOCA-salt Fine
cleaved PANX1

Figure 2C

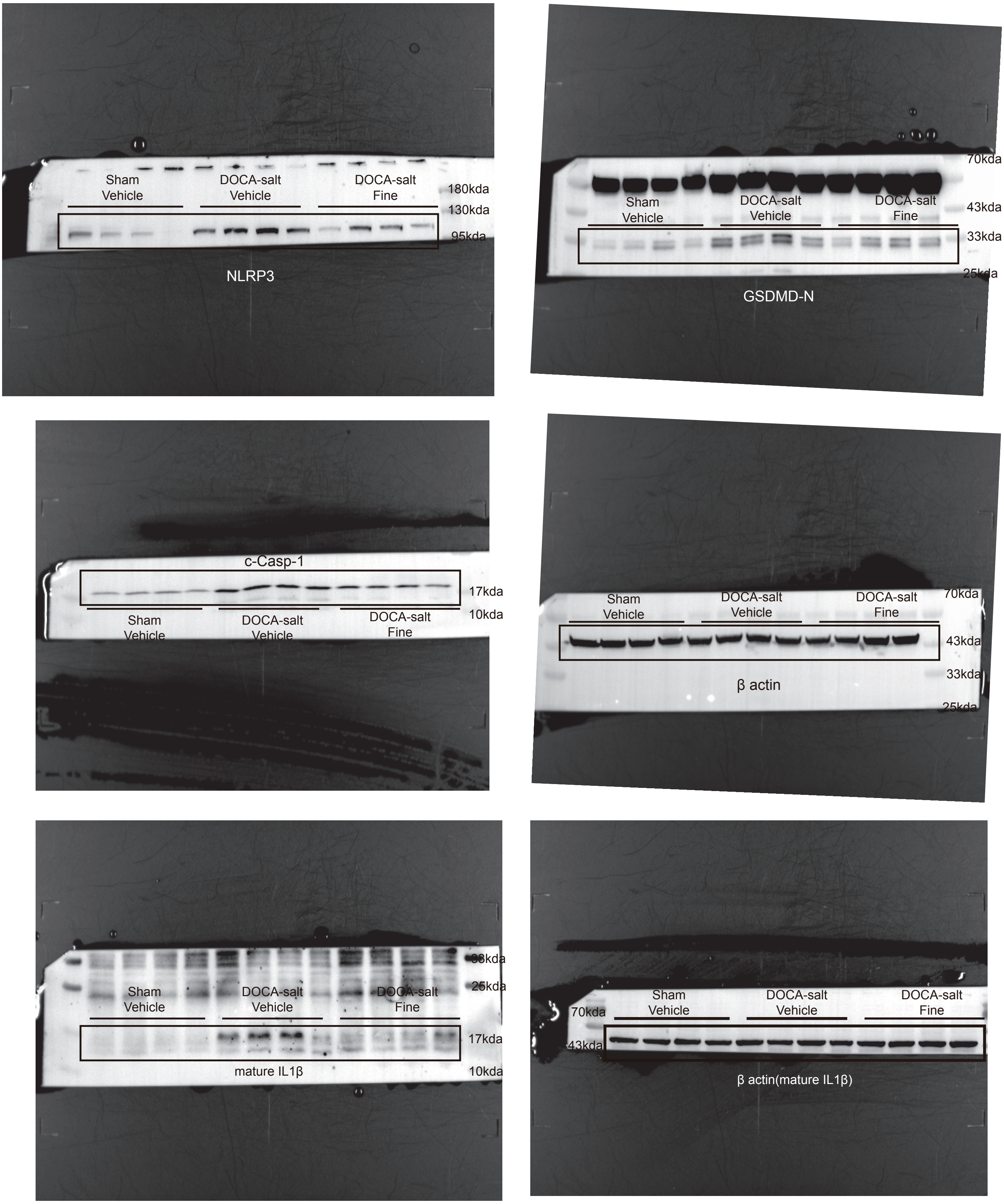

Sham
Vehicle
DOCA-salt
Vehicle
DOCA-salt
Fine
180kda
130kda
95kda
NLRP3
70kda
43kda
33kda
25kda
GSDMD-N
c-Casp-1
17kda
10kda
β actin
38kda
25kda
17kda
10kda
mature IL1β
70kda
43kda
β actin(mature IL1β)

Figure 2F

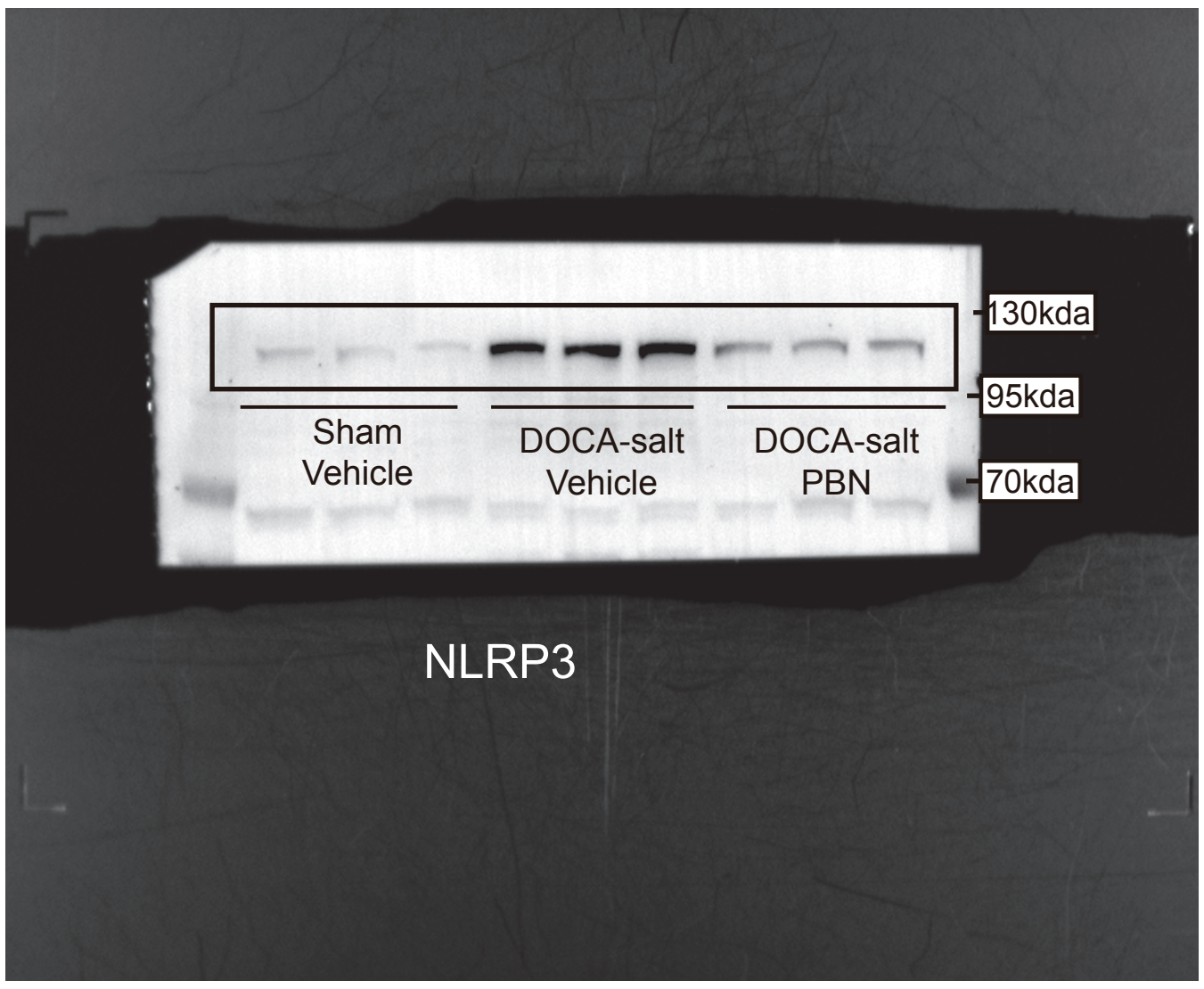
130kda
95kda
70kda
Sham Vehicle
DOCA-salt Vehicle
DOCA-salt PBN
NLRP3

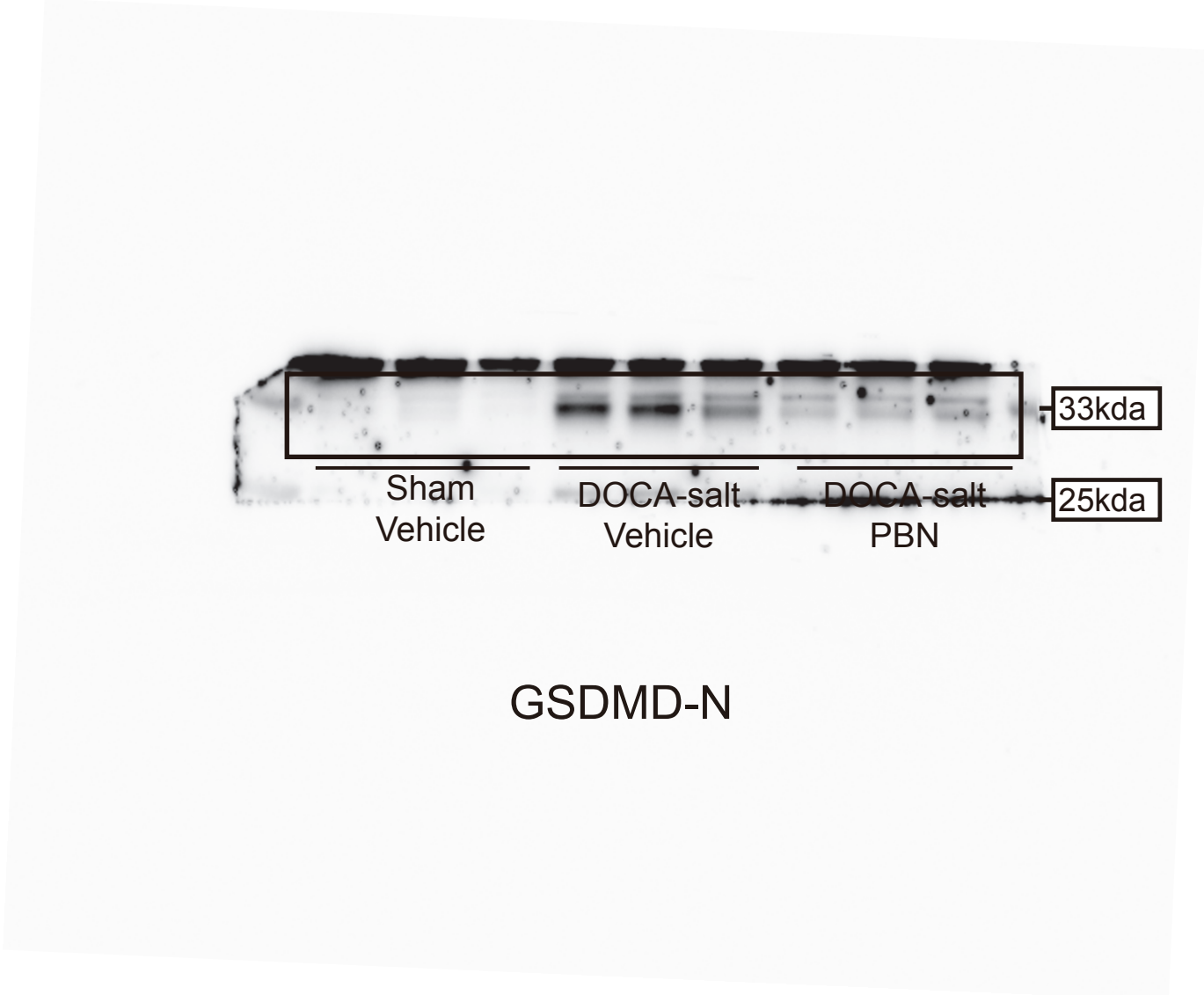
33kda
25kda
Sham Vehicle
DOCA-salt Vehicle
DOCA-salt PBN
GSDMD-N

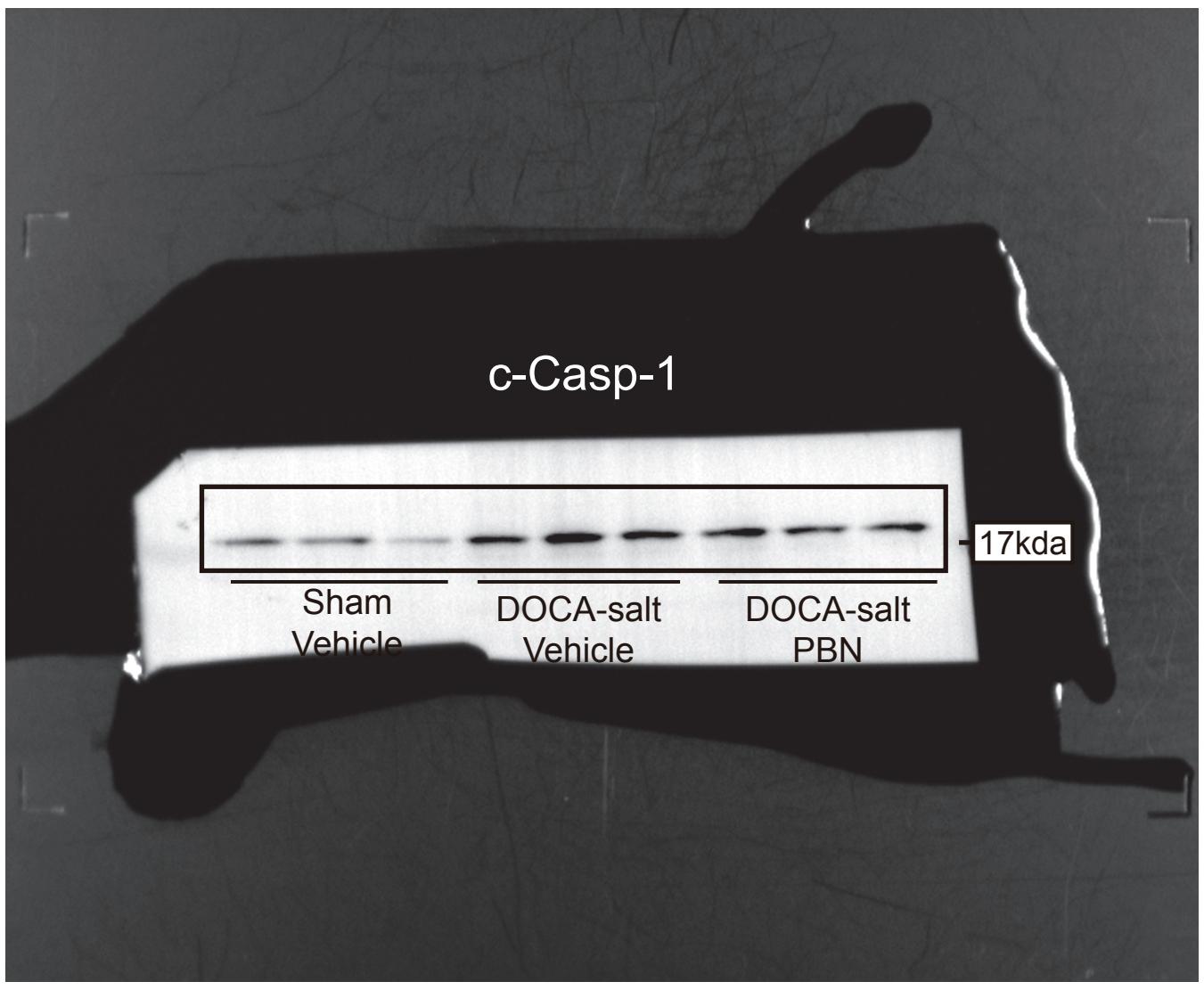
c-Casp-1
17kda
Sham Vehicle
DOCA-salt Vehicle
DOCA-salt PBN

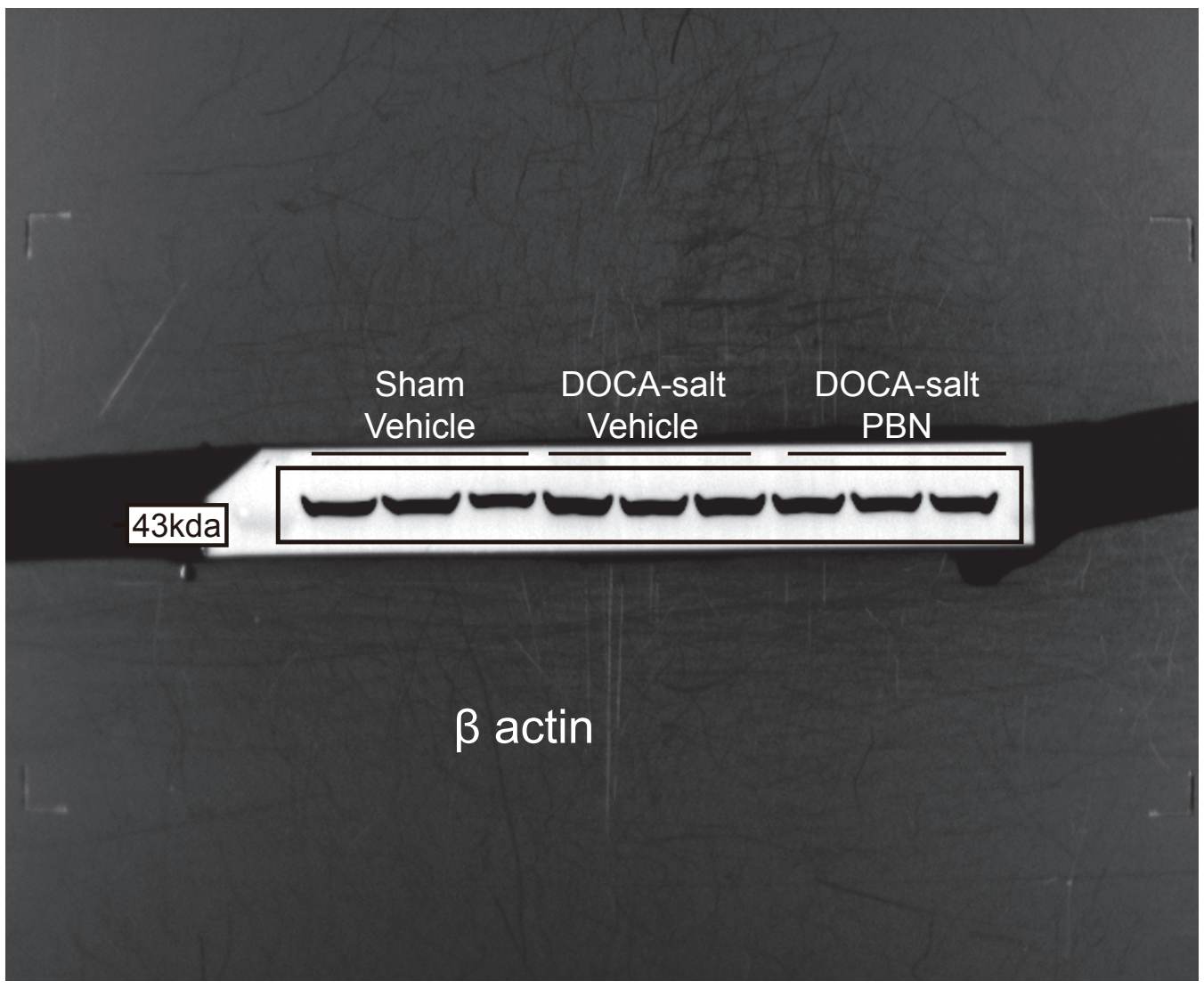
Sham Vehicle
DOCA-salt Vehicle
DOCA-salt PBN
43kda
β actin

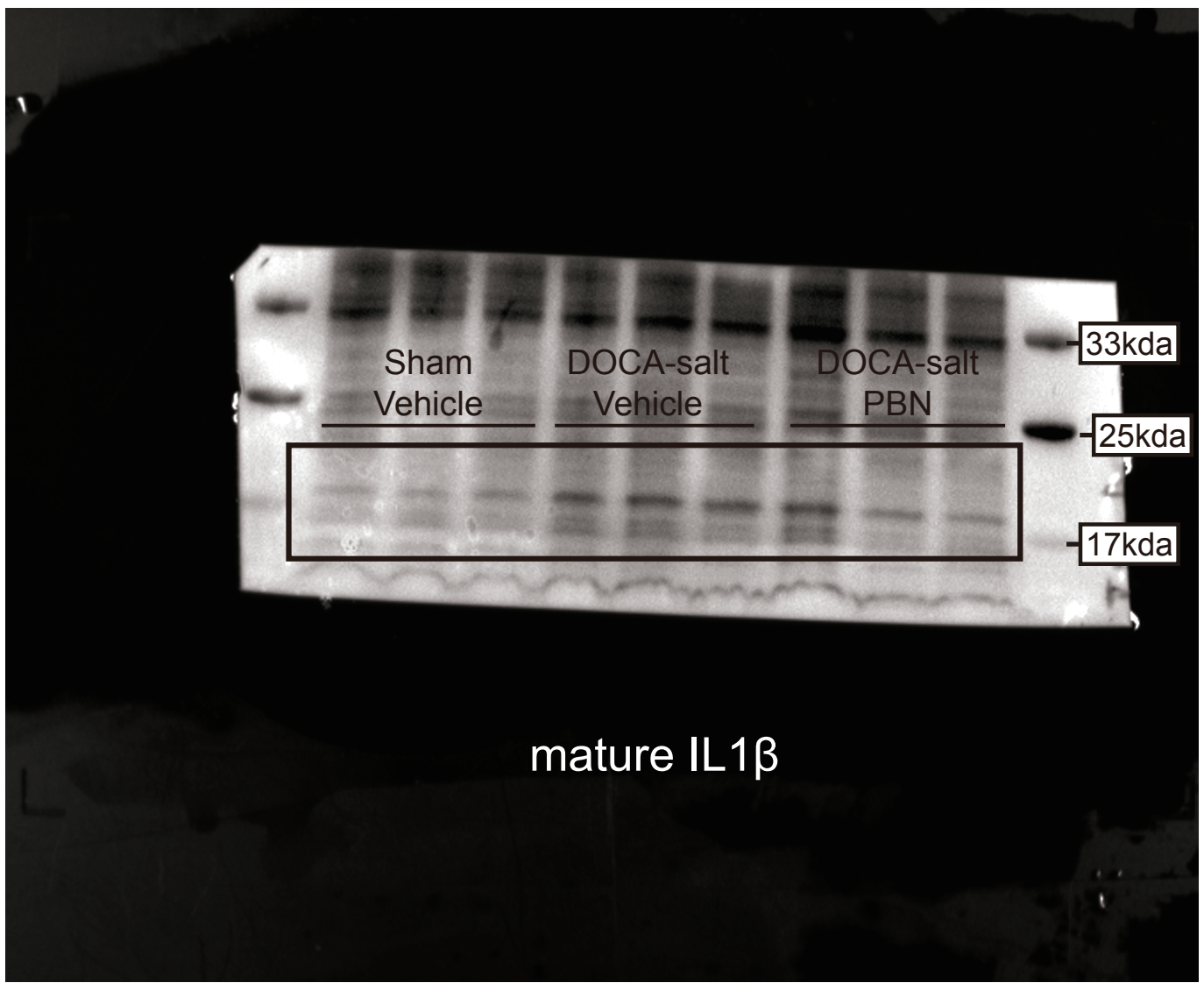
Sham Vehicle
DOCA-salt Vehicle
DOCA-salt PBN
33kda
25kda
17kda
mature IL1β

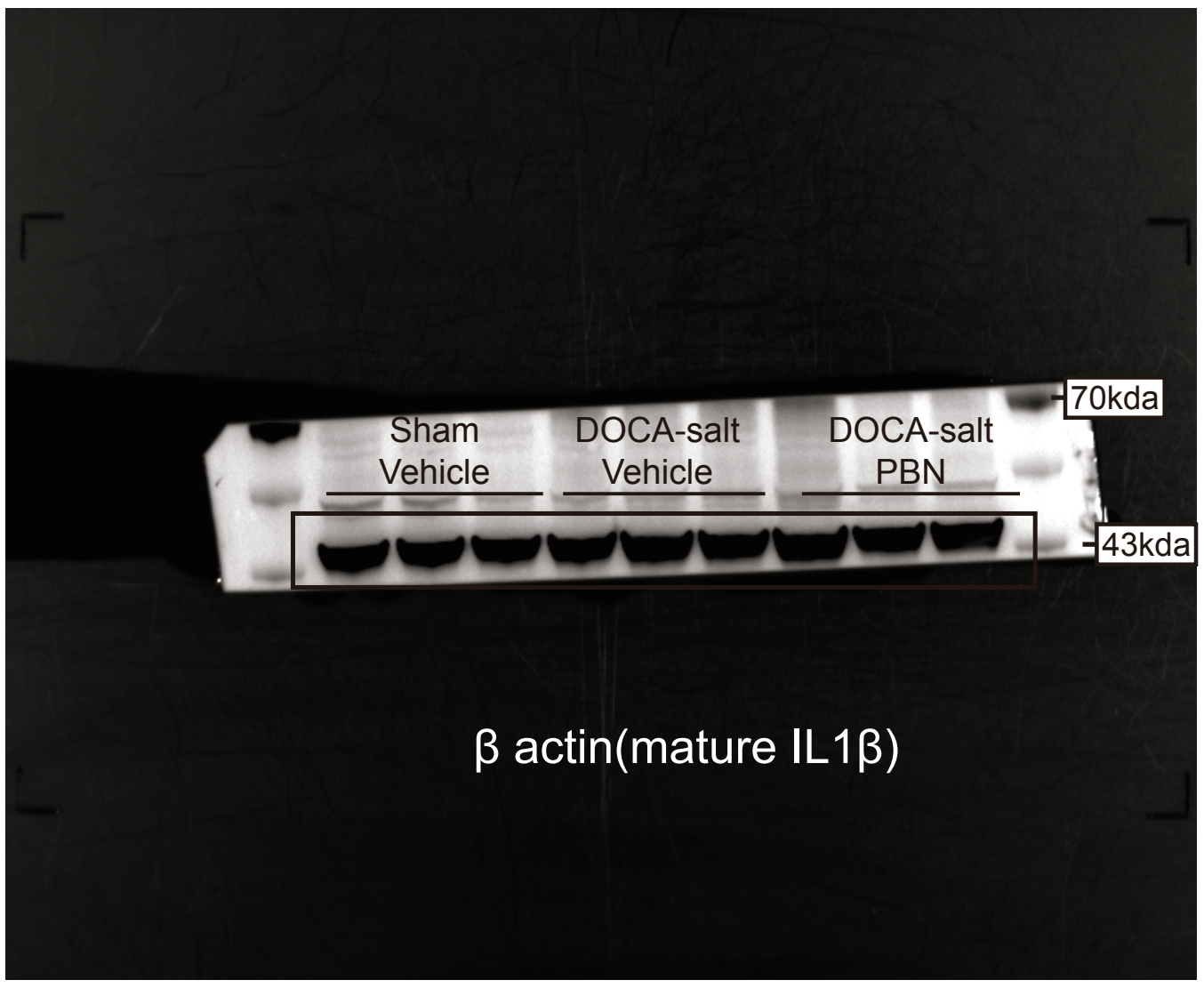
70kda
Sham Vehicle
DOCA-salt Vehicle
DOCA-salt PBN
43kda
β actin(mature IL1β)

Figure 3H

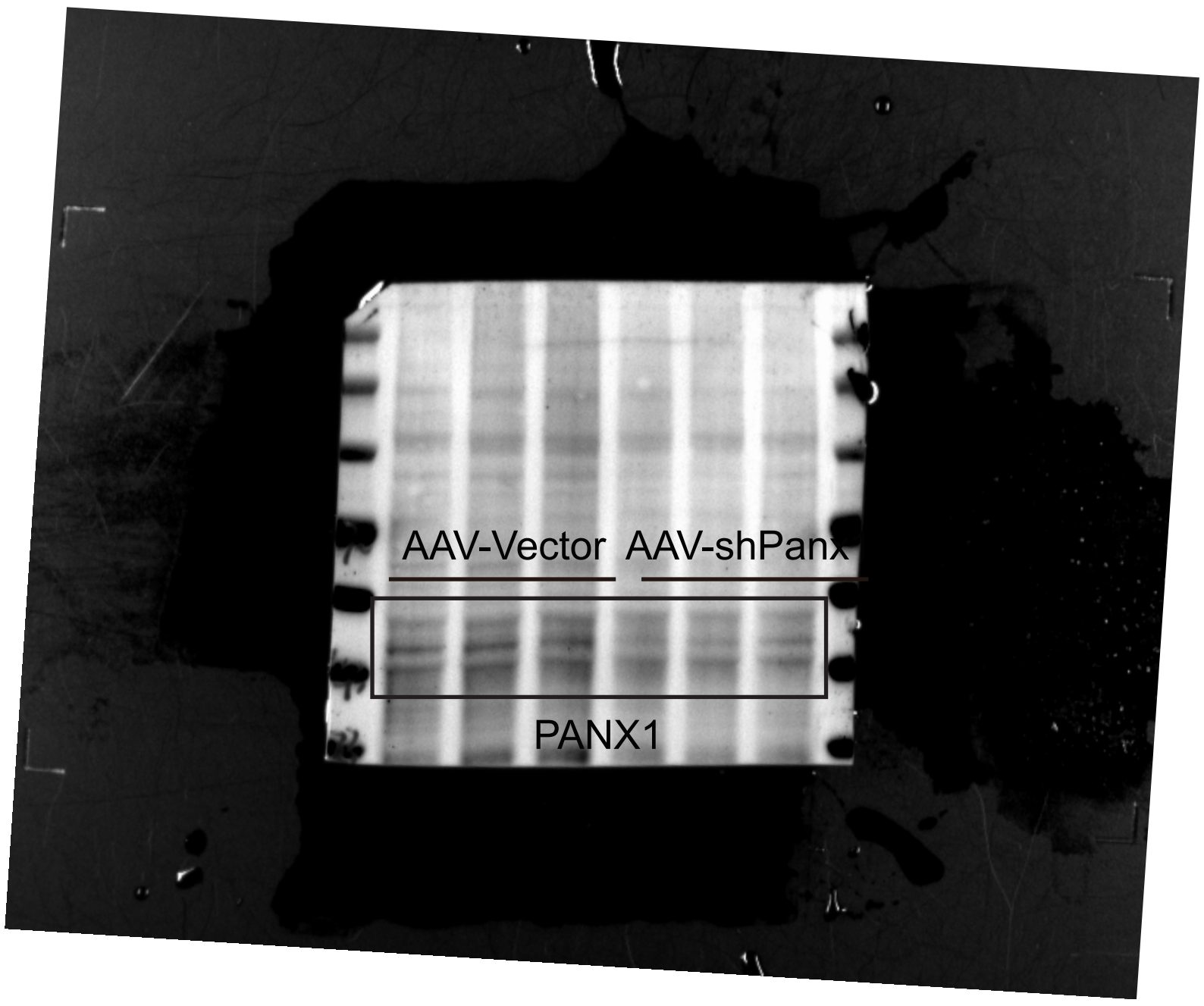

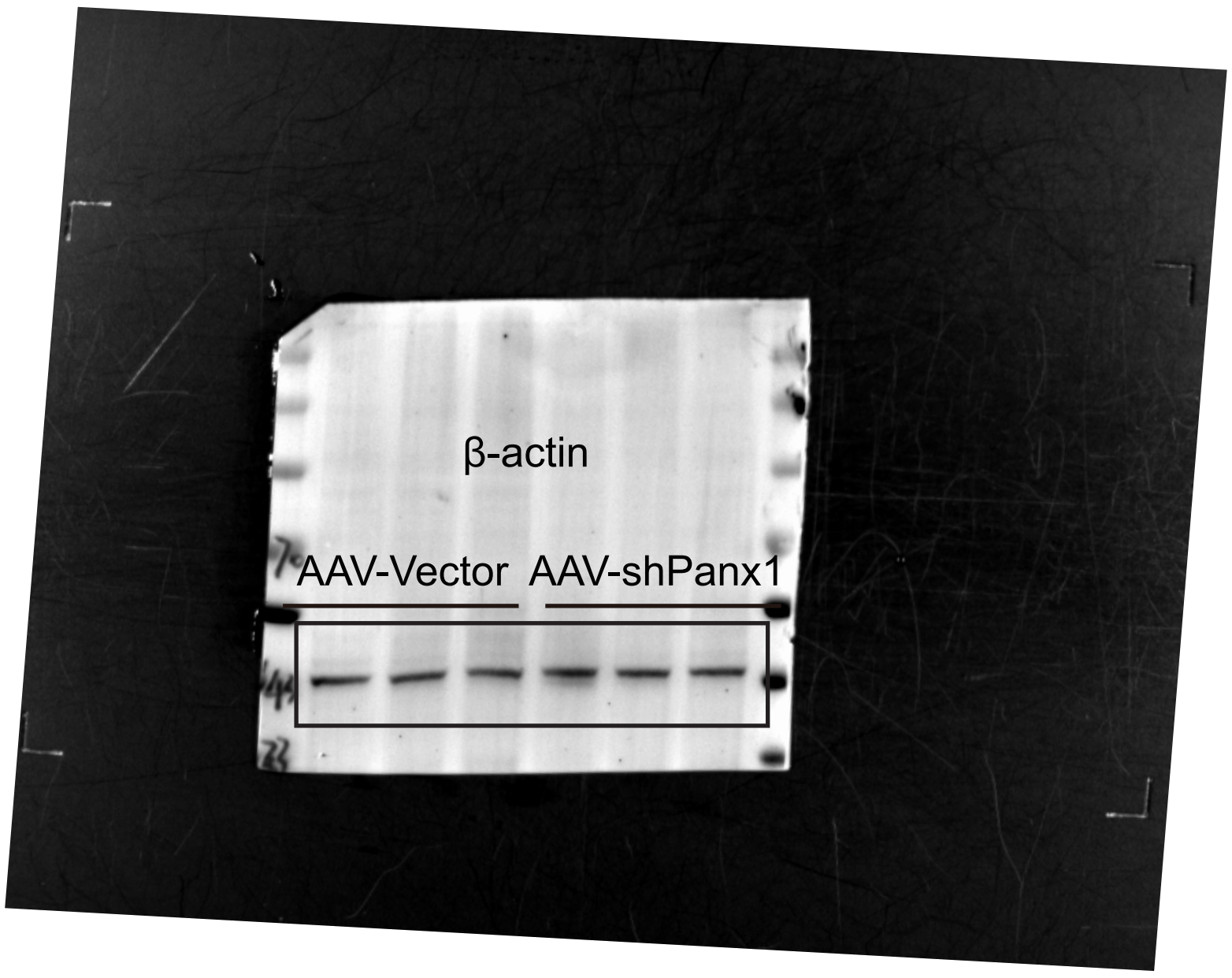

Figure 4B

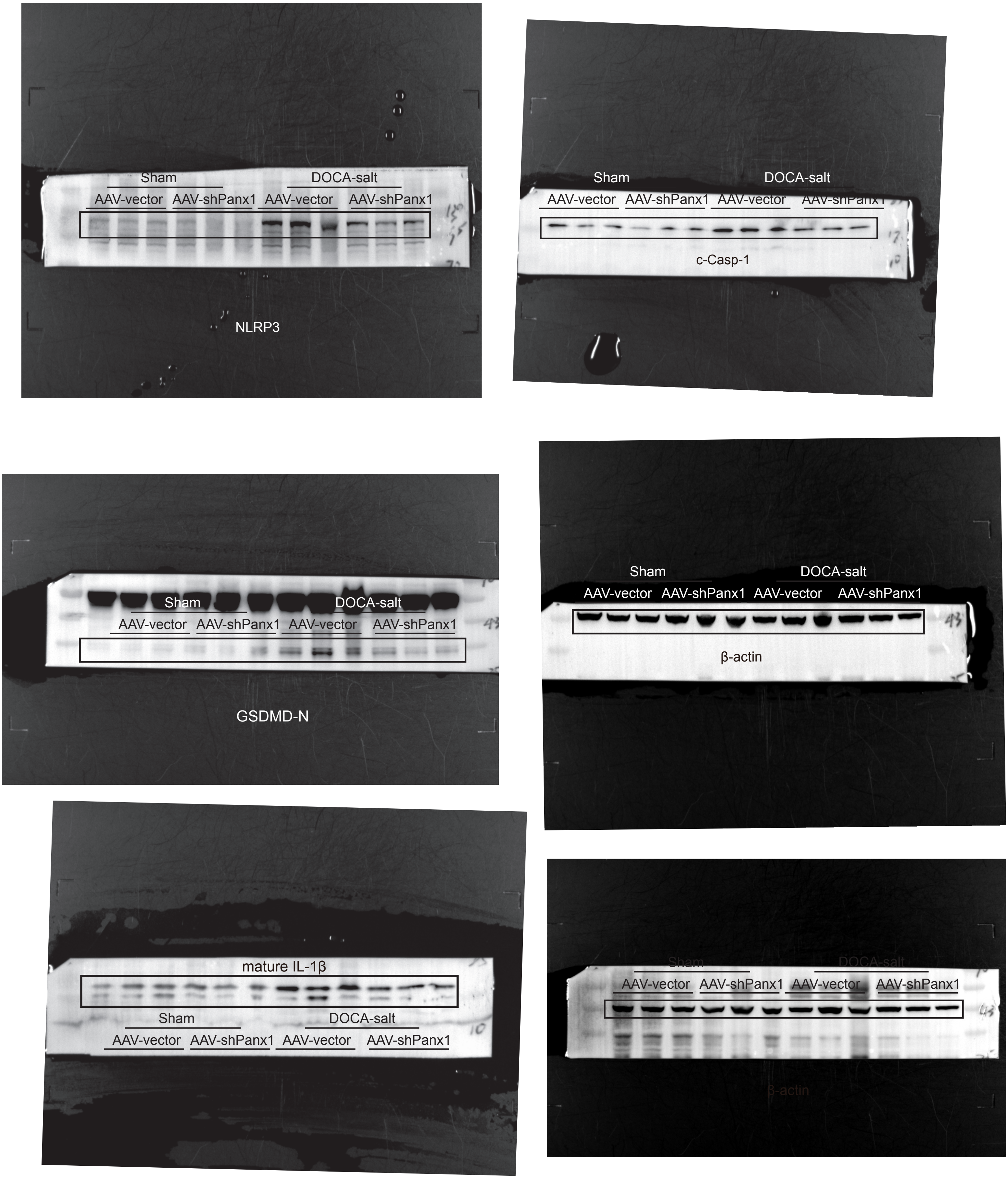
Sham
DOCA-salt
AAV-vector
AAV-shPanx1
AAV-vector
AAV-shPanx1
NLRP3
Sham
DOCA-salt
AAV-vector
AAV-shPanx1
AAV-vector
AAV-shPanx1
c-Casp-1
Sham
DOCA-salt
AAV-vector
AAV-shPanx1
AAV-vector
AAV-shPanx1
GSDMD-N
Sham
DOCA-salt
AAV-vector
AAV-shPanx1
AAV-vector
AAV-shPanx1
β-actin
mature IL-1β
Sham
DOCA-salt
AAV-vector
AAV-shPanx1
AAV-vector
AAV-shPanx1
Sham
DOCA-salt
AAV-vector
AAV-shPanx1
AAV-vector
AAV-shPanx1
β-actin

Figure 4J

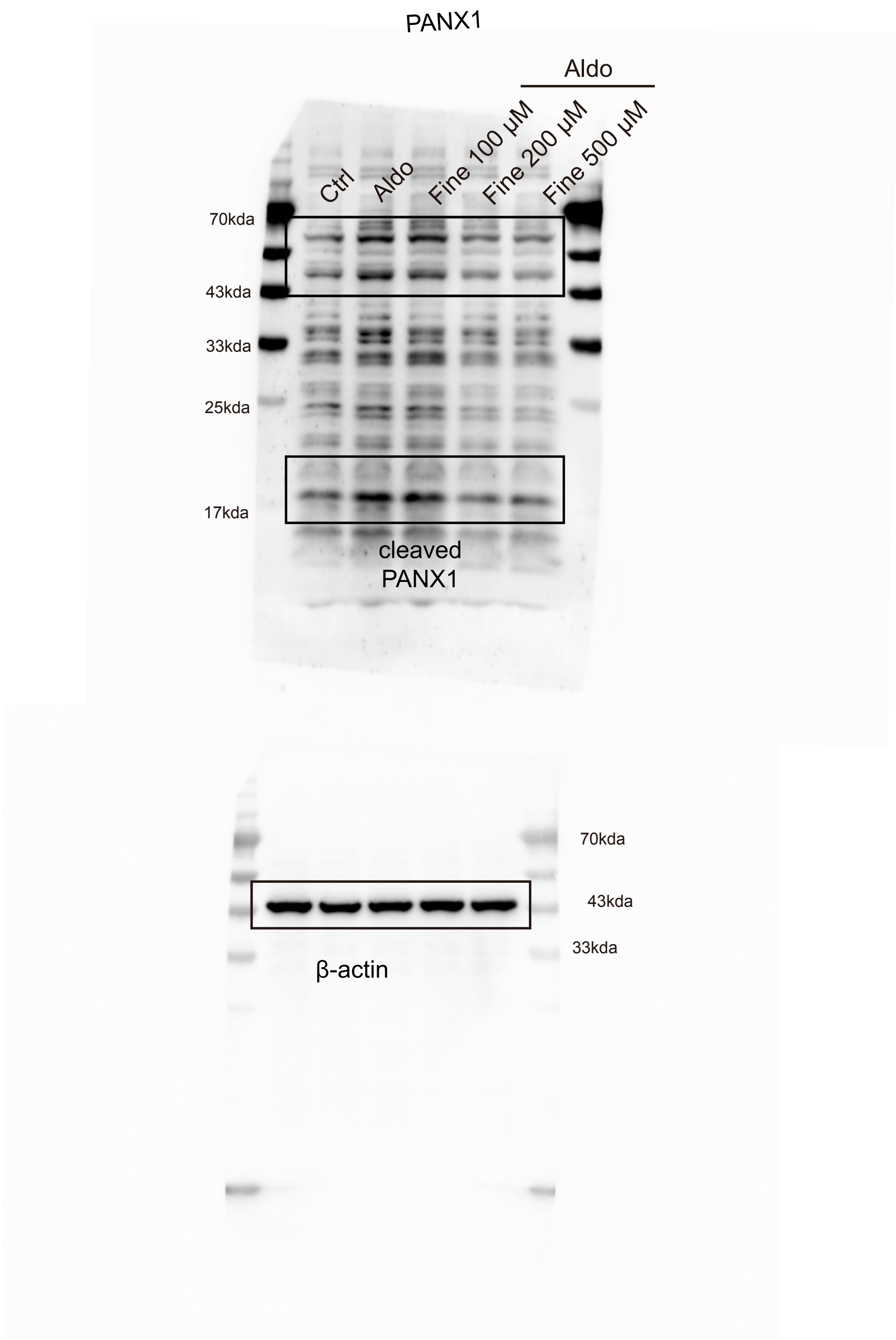
PANX1
Aldo
Ctrl
Aldo
Fine 100 μM
Fine 200 μM
Fine 500 μM
70kda
43kda
33kda
25kda
17kda
cleaved PANX1
70kda
43kda
33kda
β-actin

Figure 5C

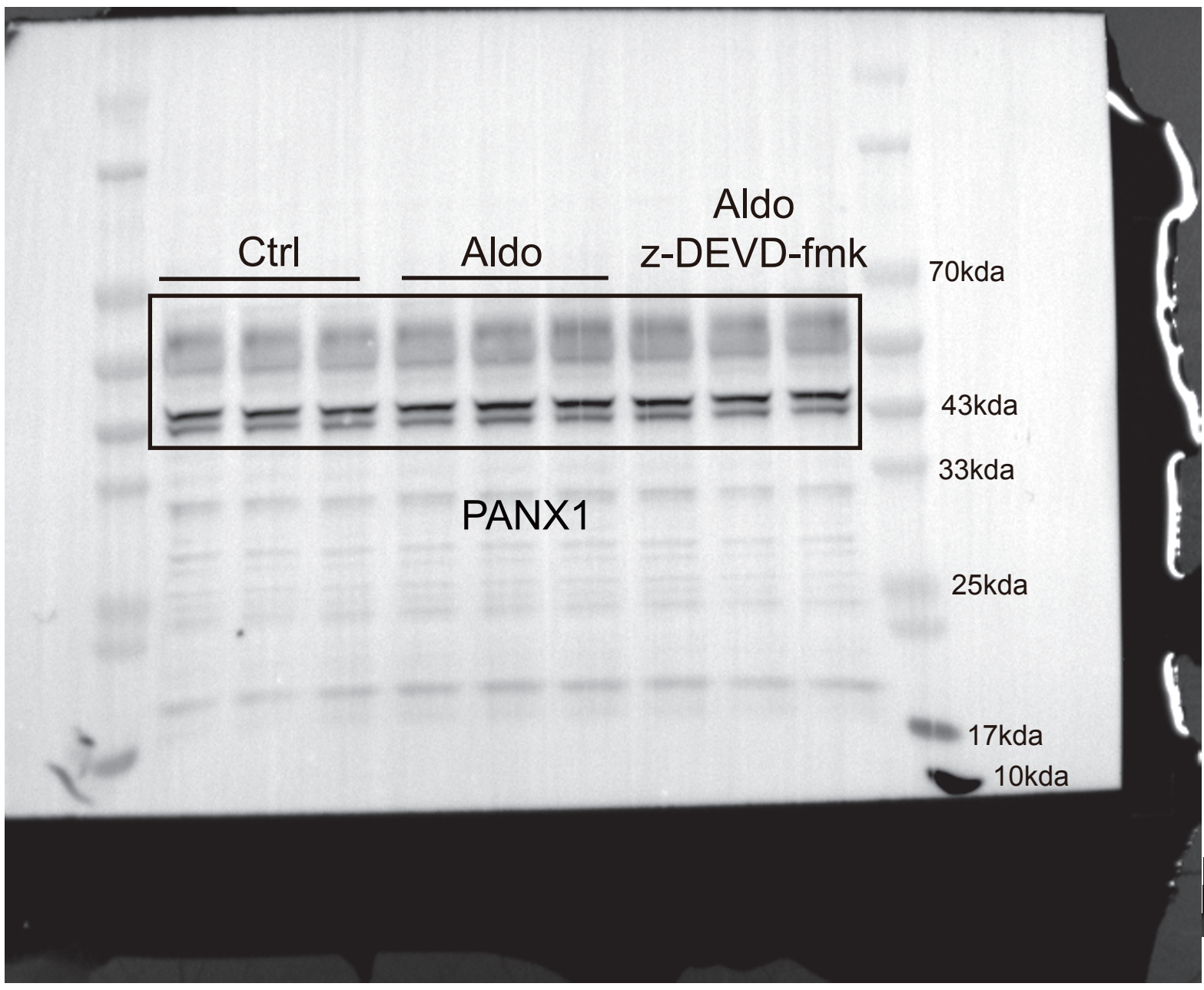

Aldo
Ctrl
Aldo
z-DEVD-fmk
70kda
43kda
33kda
PANX1
25kda
17kda
10kda

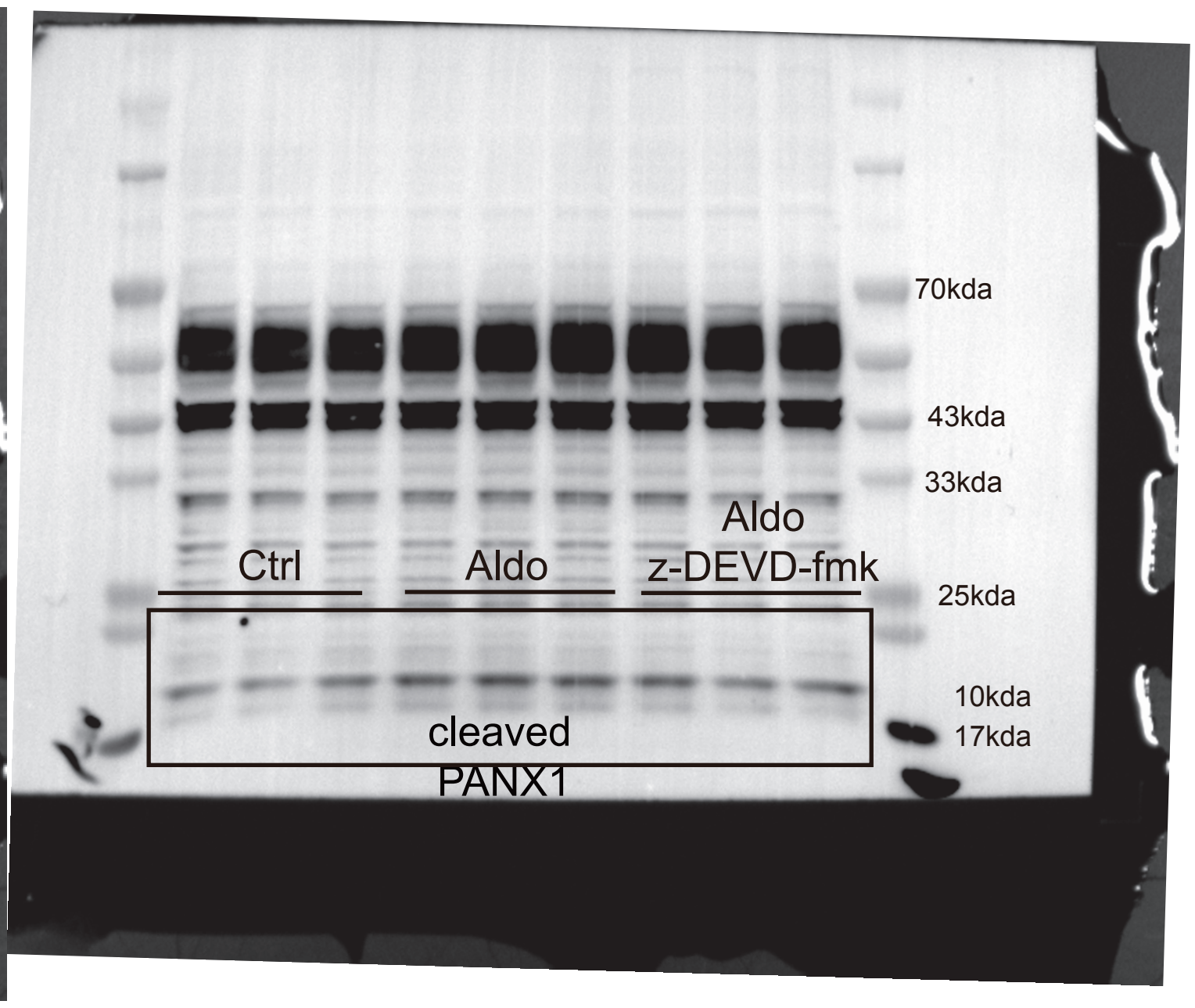

70kda
43kda
33kda
Aldo
Ctrl
Aldo
z-DEVD-fmk
25kda
10kda
cleaved
PANX1
17kda

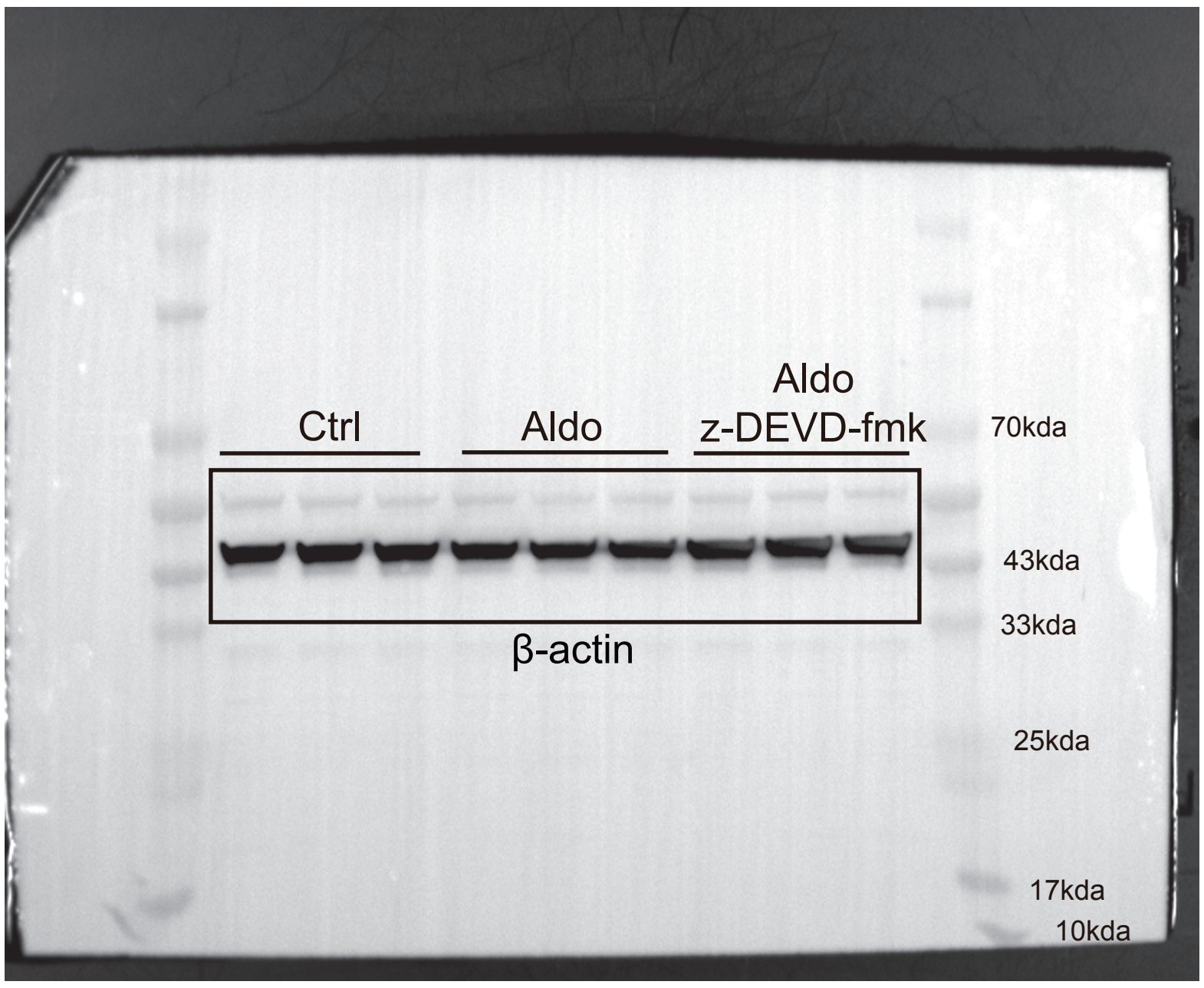

Aldo
Ctrl
Aldo
z-DEVD-fmk
70kda
43kda
33kda
β-actin
25kda
17kda
10kda

Figure 5I

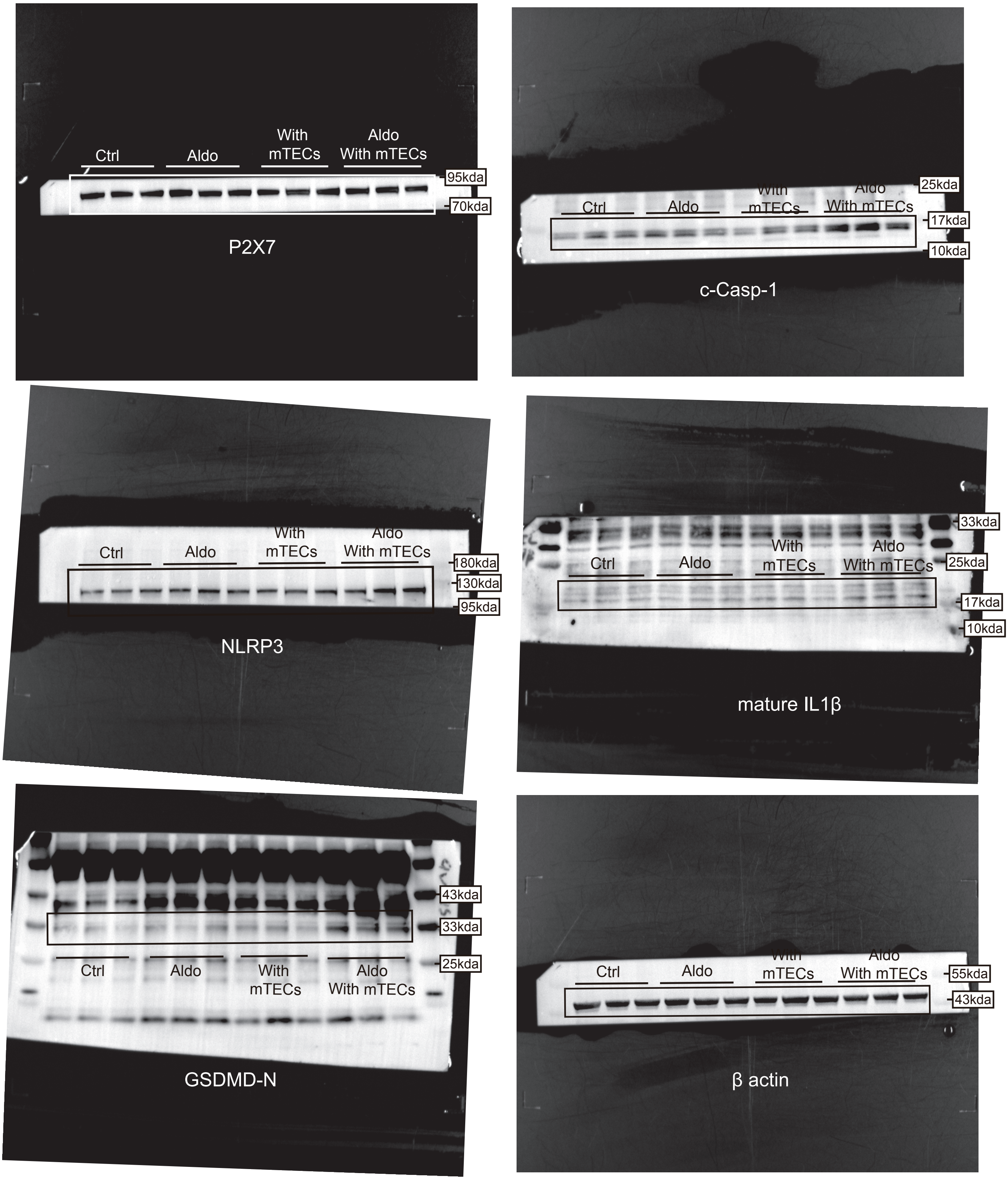
Ctrl
Aldo
With mTECs
Aldo With mTECs
95kda
70kda
P2X7
Ctrl
Aldo
With mTECs
Aldo With mTECs
25kda
17kda
10kda
c-Casp-1
Ctrl
Aldo
With mTECs
Aldo With mTECs
180kda
130kda
95kda
NLRP3
33kda
With mTECs
Aldo With mTECs
Ctrl
Aldo
25kda
17kda
10kda
mature IL1β
43kda
33kda
25kda
Ctrl
Aldo
With mTECs
Aldo With mTECs
GSDMD-N
Ctrl
Aldo
With mTECs
Aldo With mTECs
55kda
43kda
β actin

Figure 6B

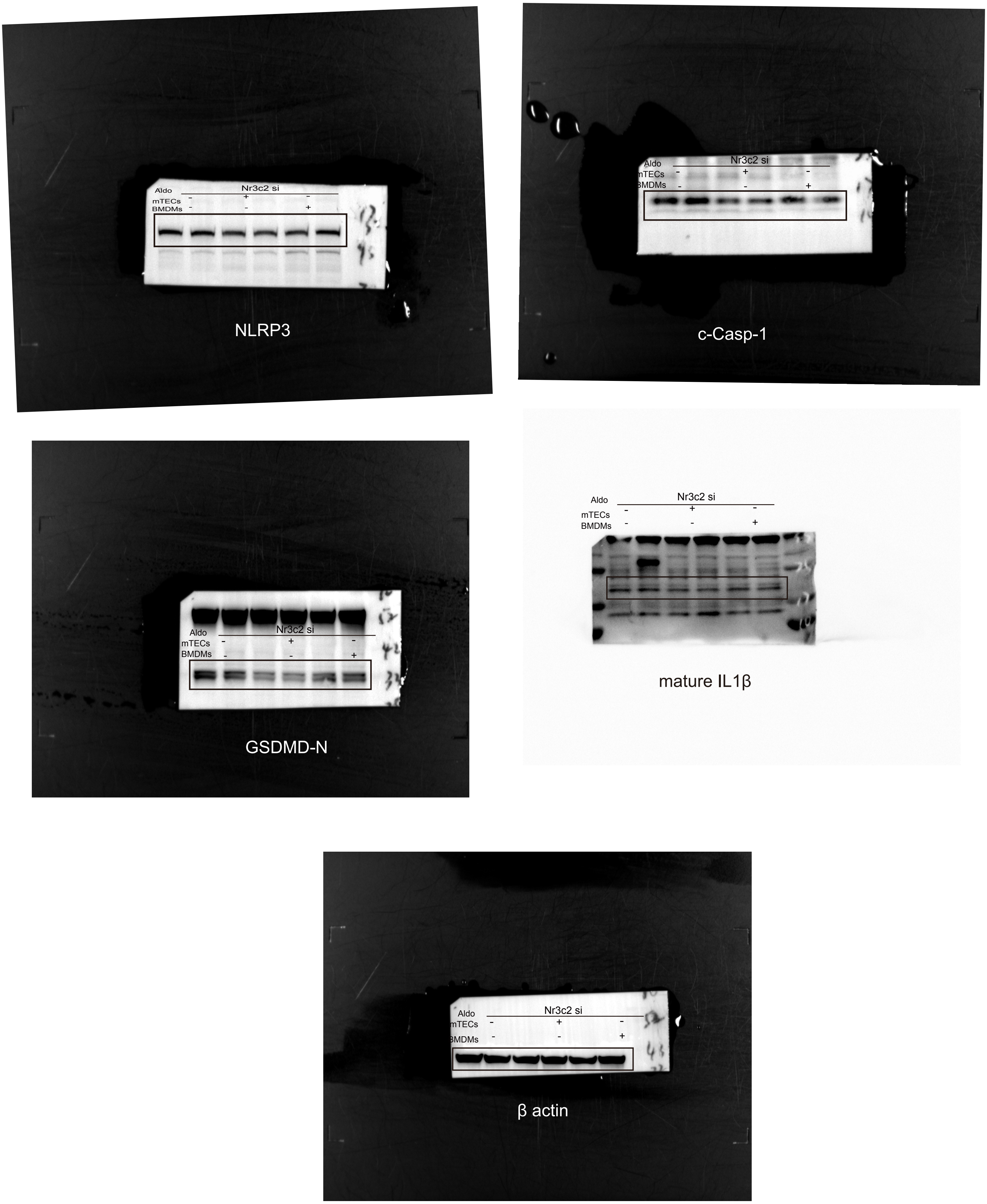
Aldo
mTECs
BMDMs
Nr3c2 si
NLRP3
c-Casp-1
GSDMD-N
mature IL1β
β actin

Figure 6F

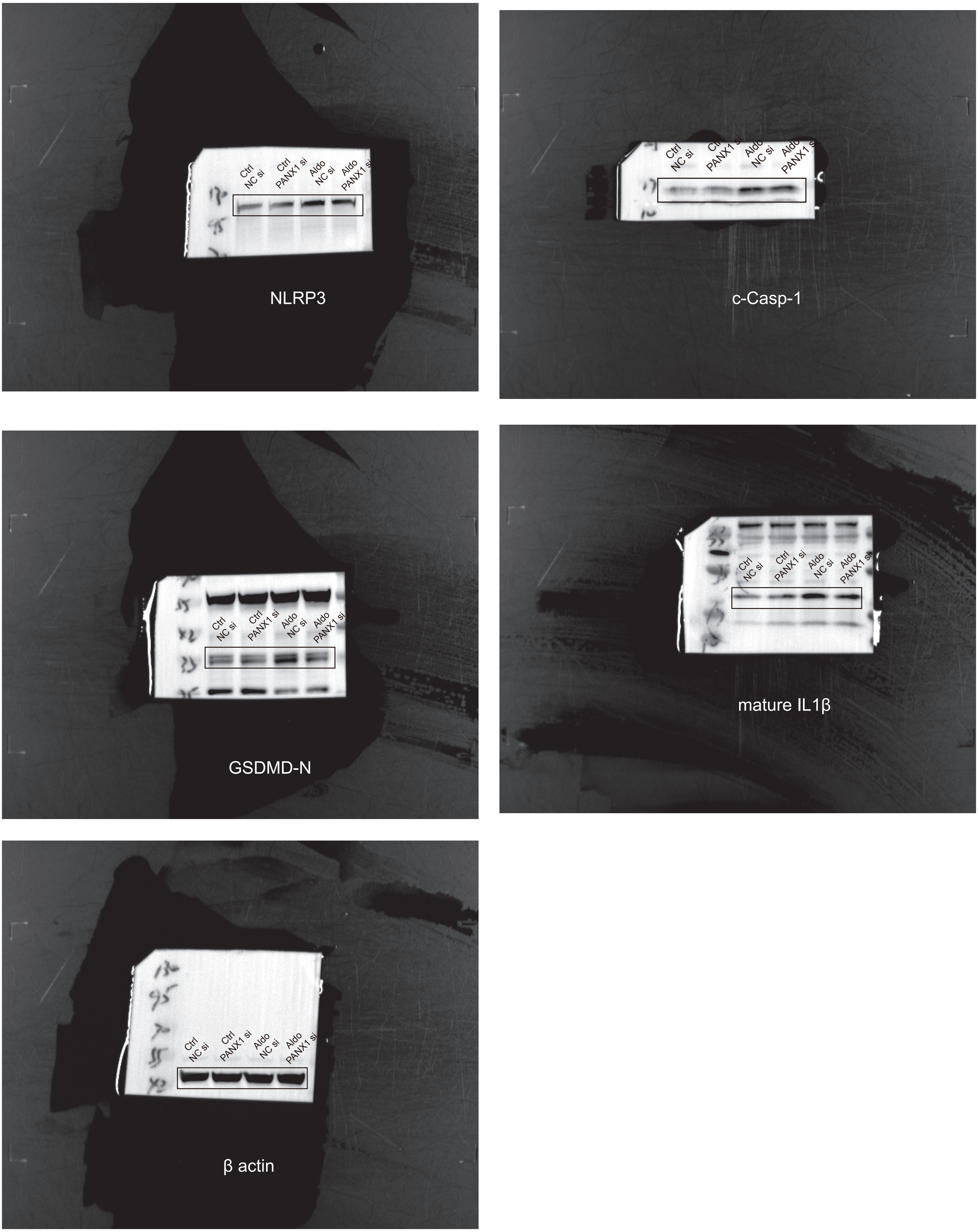
Ctrl NC si
Ctrl PANX1 si
Aldo NC si
Aldo PANX1 si
NLRP3
c-Casp-1
GSDMD-N
mature IL1β
β actin

Figure 7A

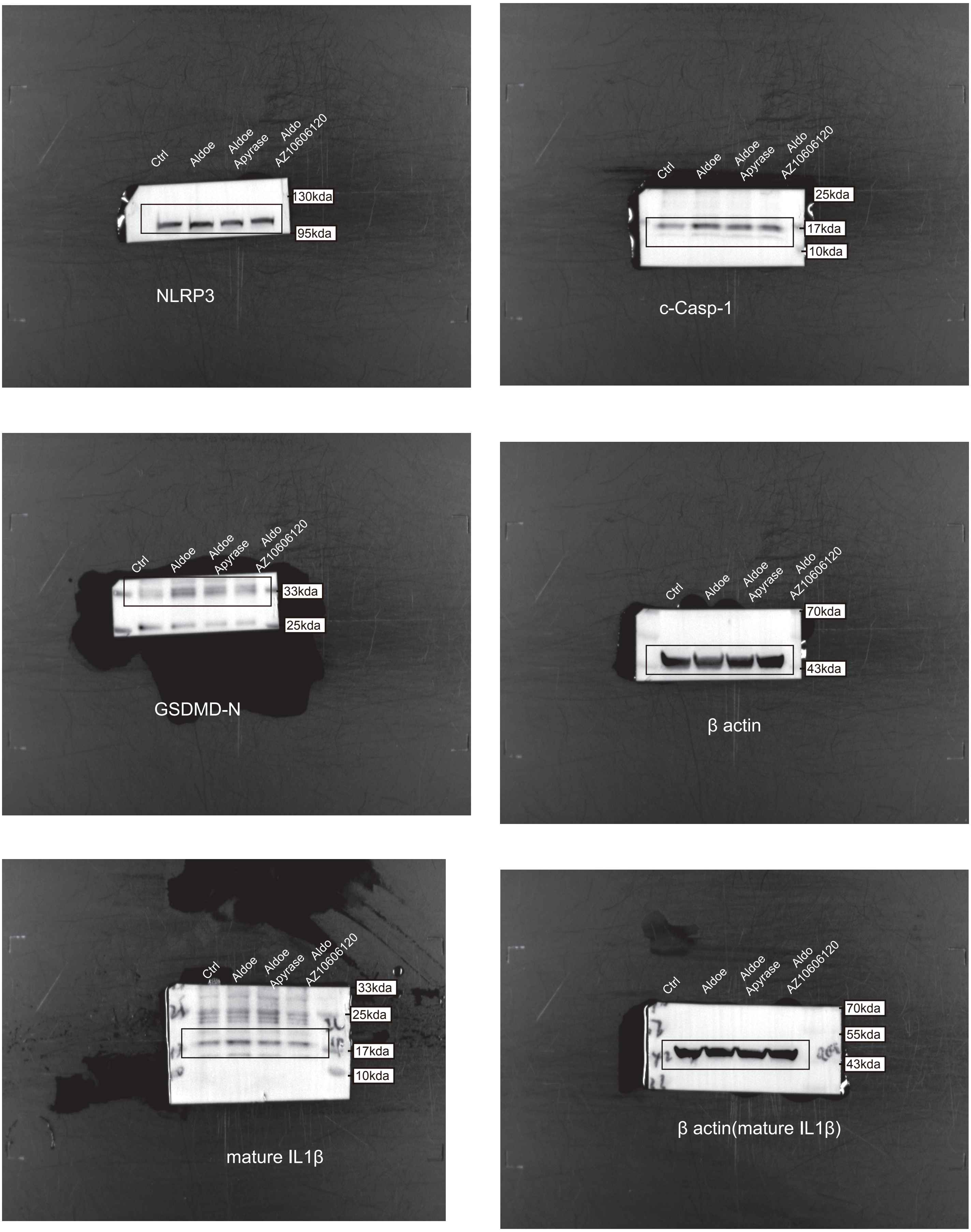
Ctrl
Aldoe
Aldoe
Apyrase
Aldo
AZ10606120
130kda
95kda
NLRP3
Ctrl
Aldoe
Aldoe
Apyrase
Aldo
AZ10606120
25kda
17kda
10kda
c-Casp-1
Ctrl
Aldoe
Aldoe
Apyrase
Aldo
AZ10606120
33kda
25kda
GSDMD-N
Ctrl
Aldoe
Aldoe
Apyrase
Aldo
AZ10606120
70kda
43kda
β actin
Ctrl
Aldoe
Aldoe
Apyrase
Aldo
AZ10606120
33kda
25kda
17kda
10kda
mature IL1β
Ctrl
Aldoe
Aldoe
Apyrase
Aldo
AZ10606120
70kda
55kda
43kda
β actin(mature IL1β)

Figure 7D

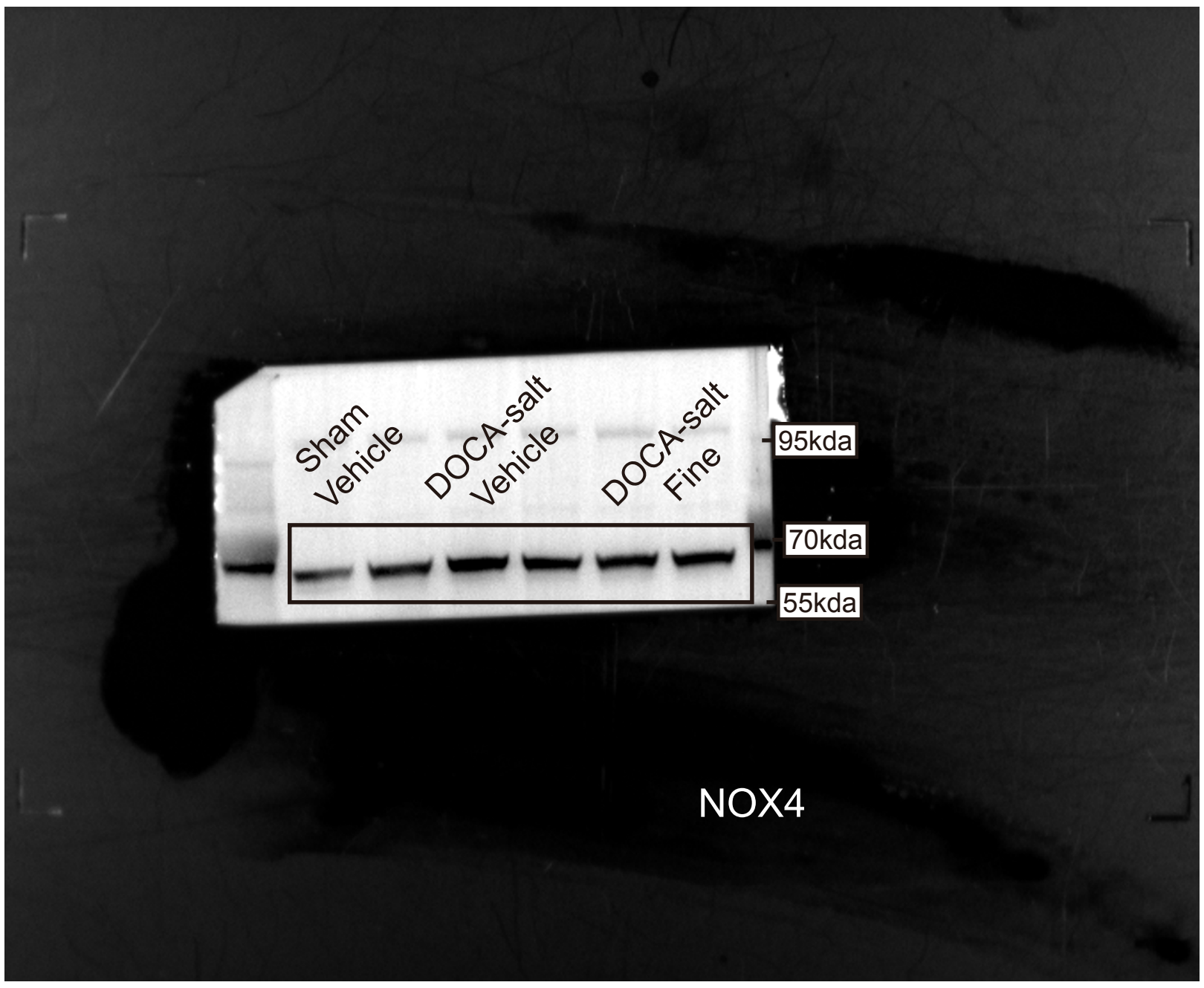
Sham
Vehicle
DOCA-salt
Vehicle
DOCA-salt
Fine
95kda
70kda
55kda
NOX4

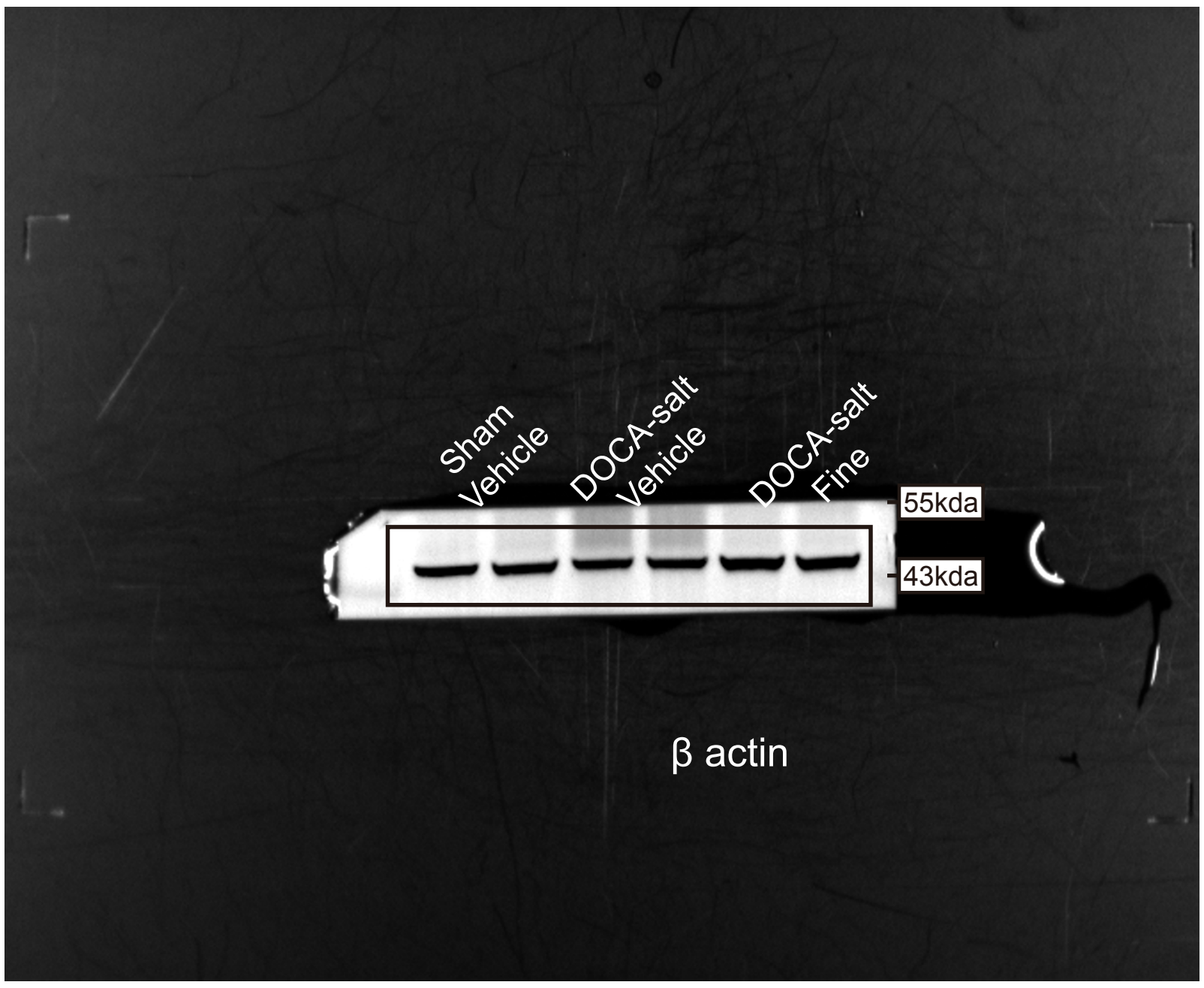
Sham
Vehicle
DOCA-salt
Vehicle
DOCA-salt
Fine
55kda
43kda
β actin

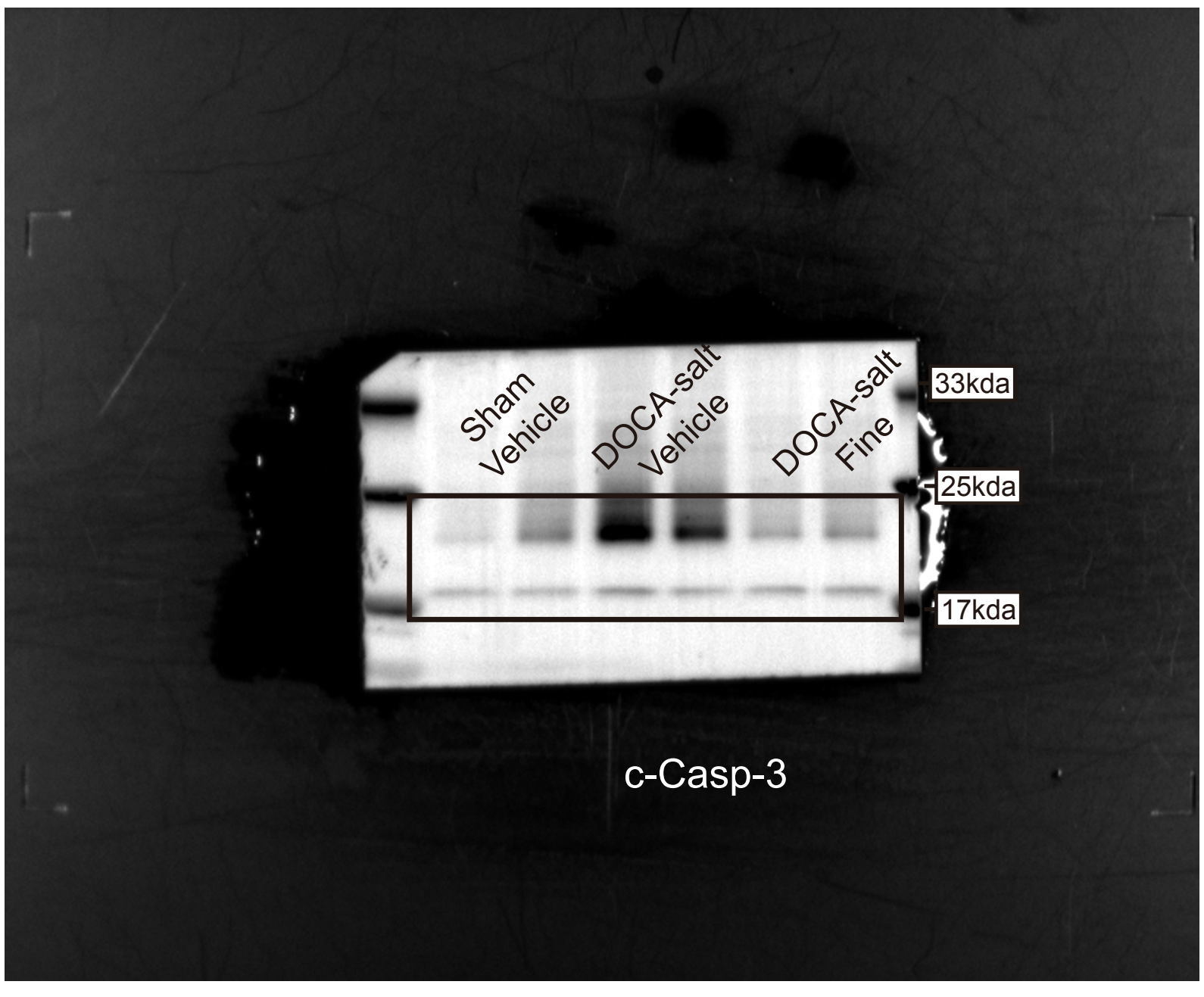
Sham
Vehicle
DOCA-salt
Vehicle
DOCA-salt
Fine
33kda
25kda
17kda
c-Casp-3

sFigure 1B

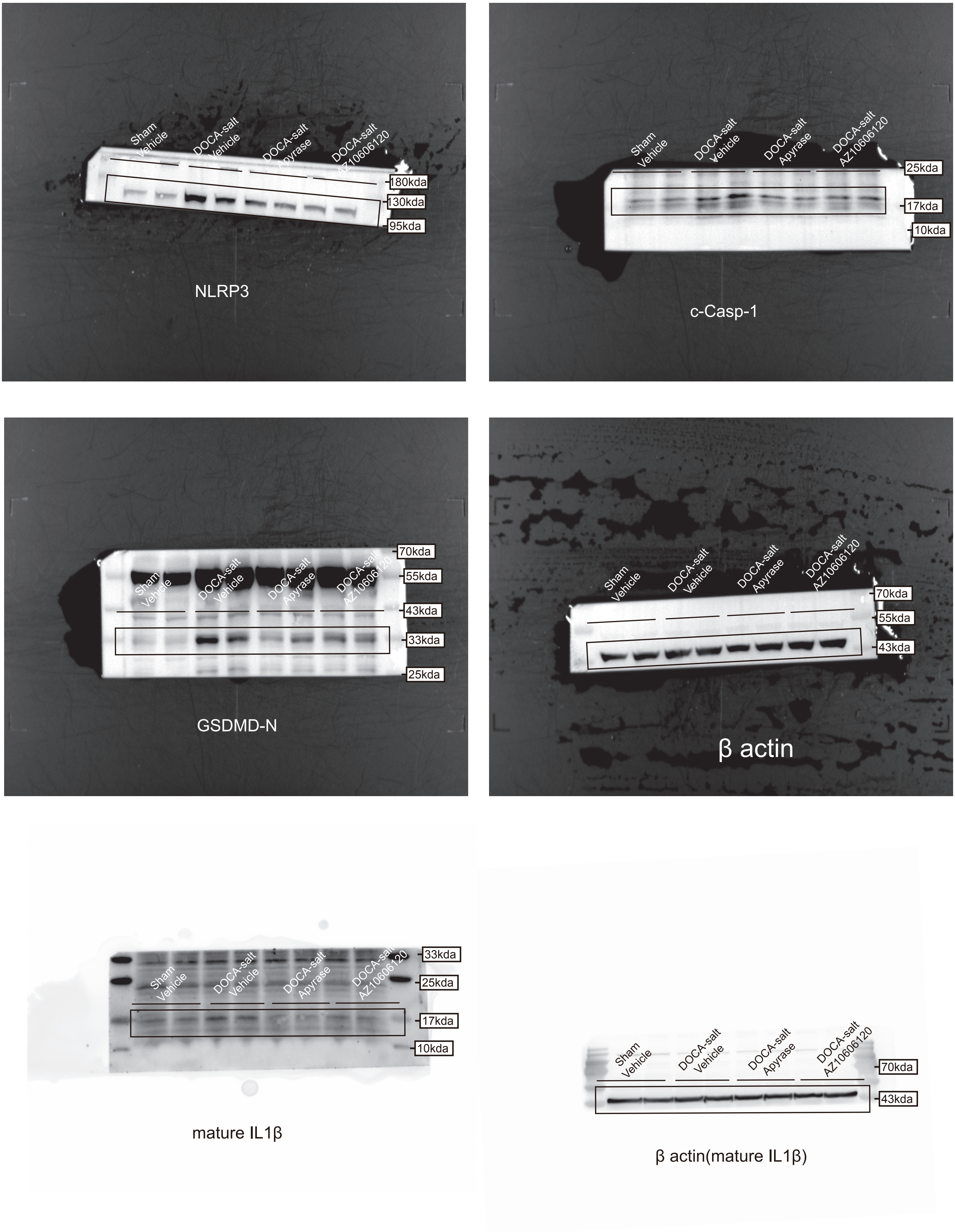
Sham Vehicle
DOCA-salt Vehicle
DOCA-salt Apyrase
DOCA-salt AZ10606120
180kda
130kda
95kda
NLRP3
25kda
17kda
10kda
c-Casp-1
70kda
55kda
43kda
33kda
25kda
GSDMD-N
70kda
55kda
43kda
β actin
33kda
25kda
17kda
10kda
mature IL1β
70kda
43kda
β actin(mature IL1β)

sFigure 4G

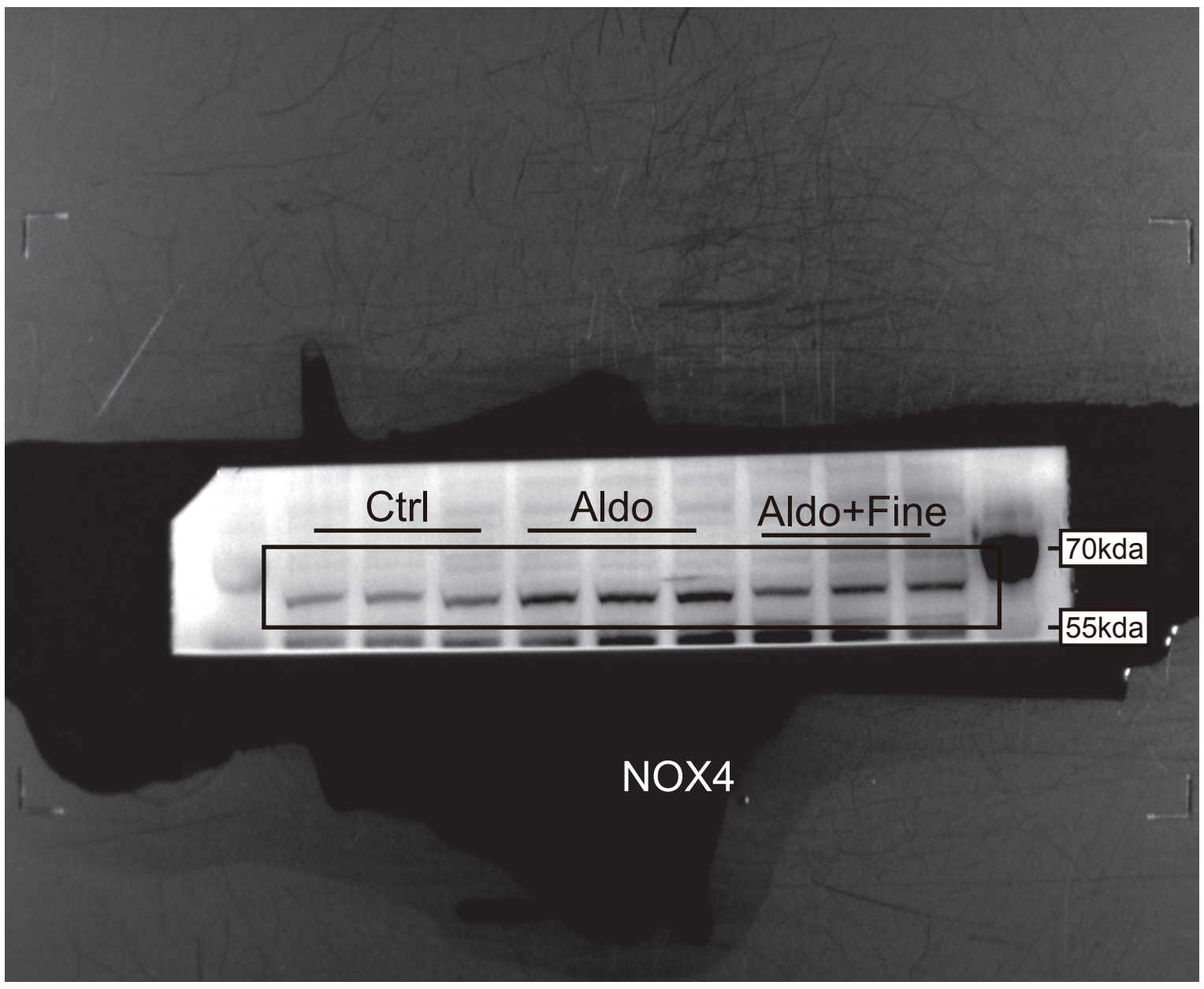
Ctrl
Aldo
Aldo+Fine
70kda
55kda
NOX4

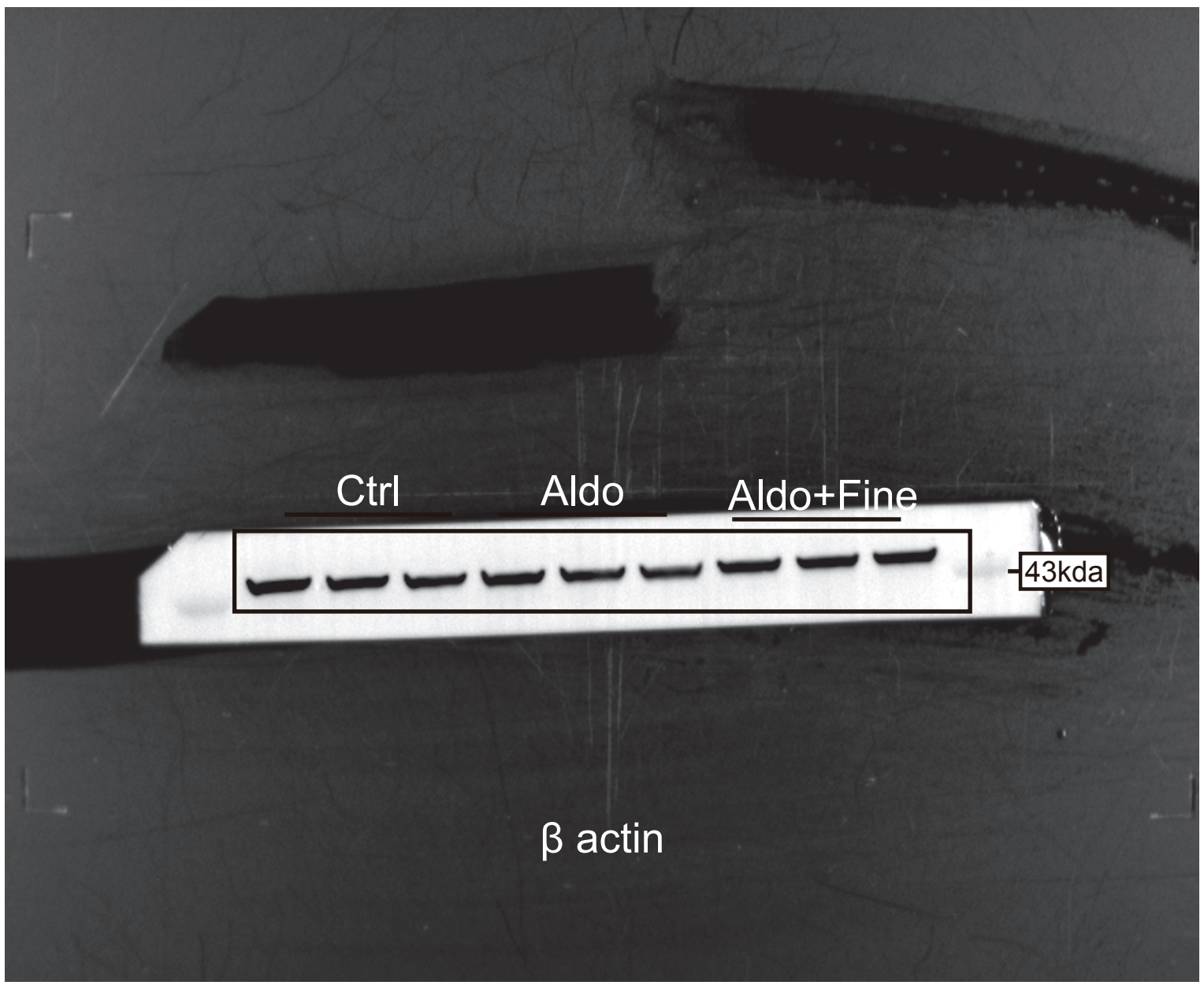
Ctrl
Aldo
Aldo+Fine
43kda
β actin

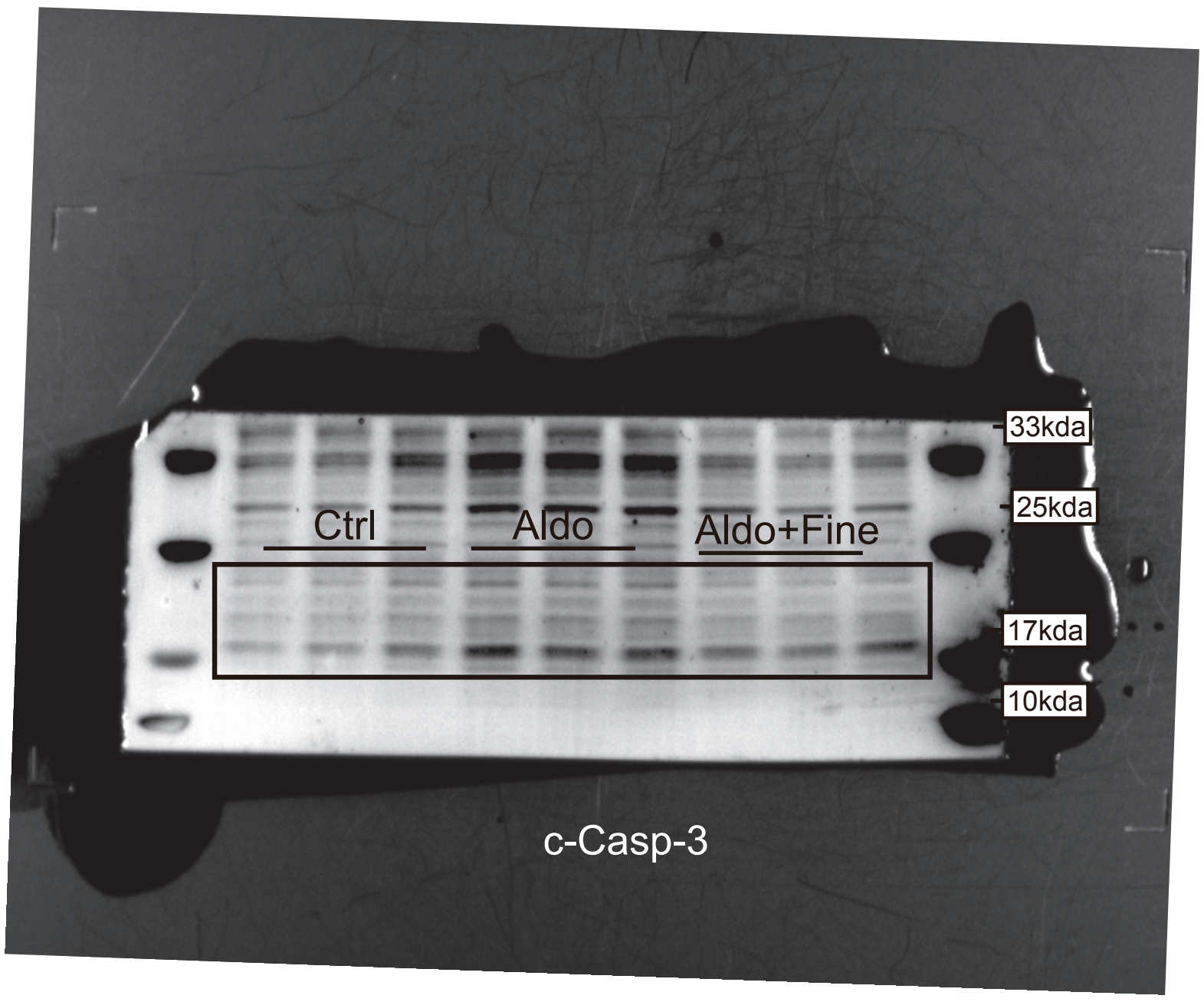
33kda
25kda
Ctrl
Aldo
Aldo+Fine
17kda
10kda
c-Casp-3

sFigure 6A

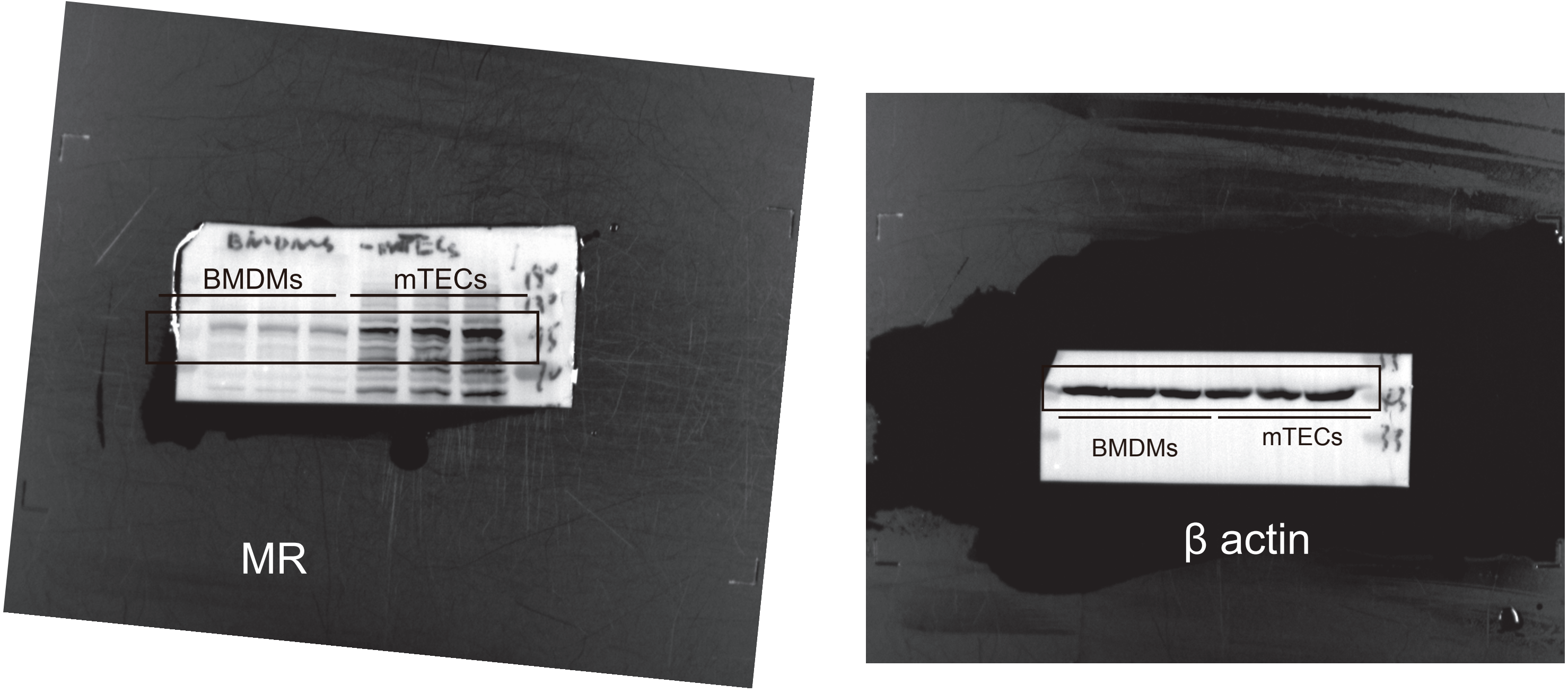

sFigure 7A

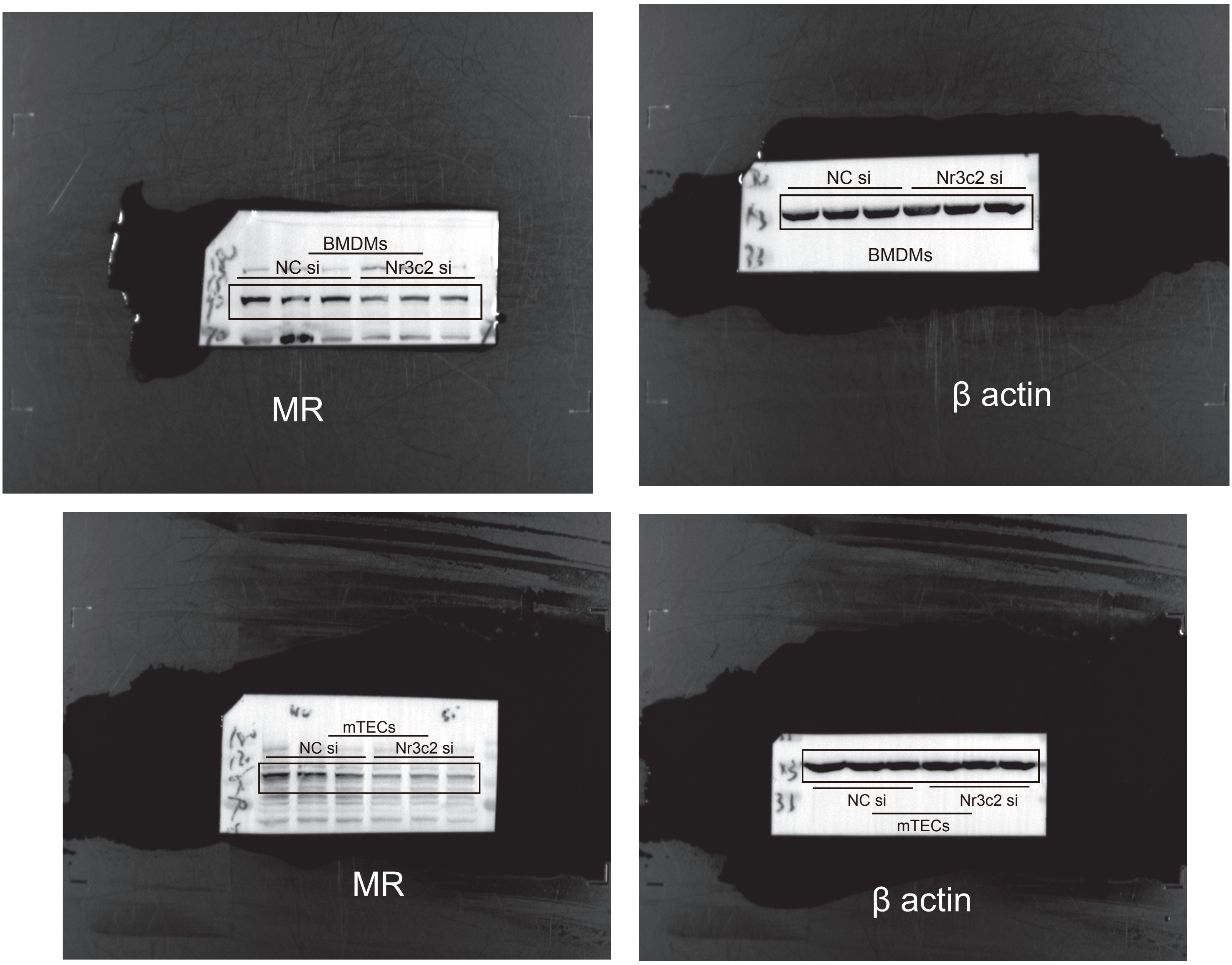

sFigure 7B

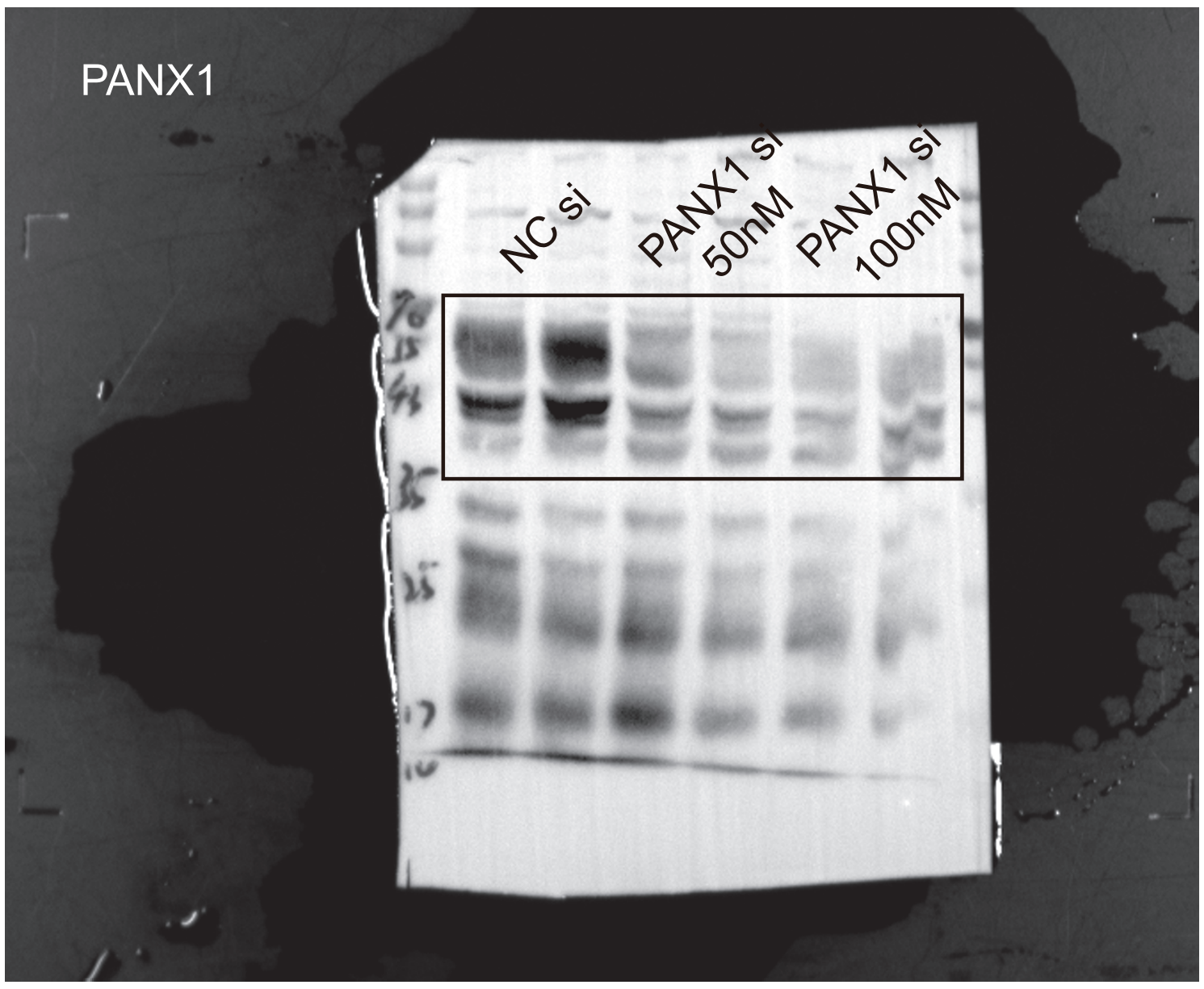
PANX1
NC si
PANX1 si
50nM
PANX1 si
100nM

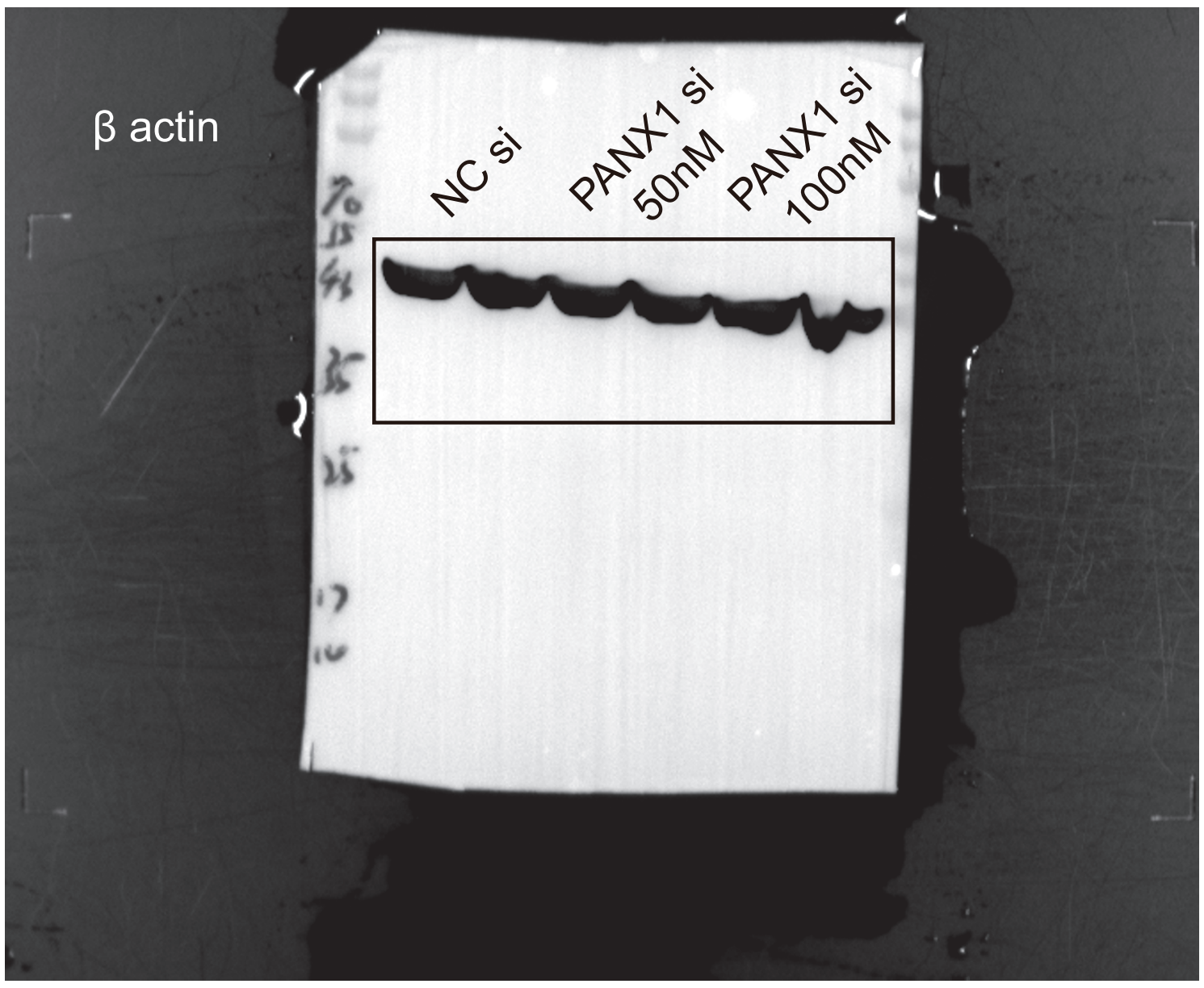
β actin
NC si
PANX1 si
50nM
PANX1 si
100nM

sFigure 8A
