# Supplementary material for: Tubular epithelial cells promote macrophage pyroptosis through PANX1-extracellular ATP-P2X7 signaling in aldosterone-dependent renal injury
Source: Cell Death Dis. 2026 Jun 5;17(1):682. doi: 10.1038/s41419-026-08951-3 (PMC13438567; doi:10.1038/s41419-026-08951-3)
Supplement: Supplementary file 3 [file 41419_2026_8951_MOESM1_ESM.docx]

**Supplemental Figure Legends**

**sFigure 1. Finerenone relieves oxidative stress in DOCA-salt mice.** (A) Representative dihydroergotamine (DHE) images, magnified views of the kidney and summarized data of DHE intensity (fold change) for different groups of mice; magnification: 200×; scale bar: 200 µm; n = 4 per group with 5 independent fields per section. (B) Representative images and summarized data showing the changes in NOX4 and cleaved Caspase-3 (c-Casp-3) expression; n = 4 per group. (C) Representative images of TUNEL staining in mouse kidney sections from indicated groups. Magnification: 400×; scale bar: 100 µm; white arrow: positive staining；n = 3 per group. ^ns^p > 0.05, * p < 0.05, **p < 0.01, and ***p < 0.001; one-way ANOVA with Tukey’s test for multiple comparisons.

**sFigure 2. PANX1 but not Cx43 is responsible for hypertensive renal injury.** (A) Changes in the level of the Cx43 (*Gja1*) mRNA in DOCA-salt mice; n = 6 per group according to an unpaired t test. (B) Data from Nephroseq (https://www.nephroseq.org) revealed elevated PANX1 and no change in Cx43 in the tubulointerstitium of arterial hypertension patients (n = 20) compared with healthy living donors (n = 31) according to an unpaired t test. (C) Positive correlation between serum creatinine levels (mg/dl) and renal tubulointerstitial *Panx1* mRNA expression in the above patients. By correlation analysis.

**sFigure 3. Dose-dependent effects of PBN, apyrase and AZ10606120 in DOCA-salt mice.** (A) Changes in the systolic pressure of DOCA-salt mice treated with PBN (50 mg/kg or 100 mg/kg), apyrase (0.2 U/g or 0.4 U/g) and AZ10606120 (0.5 mg/kg or 1 mg/kg); n = 3 per group; ^ns^p > 0.05, * p < 0.05, **p < 0.01, and ***p < 0.001; one-way ANOVA with Tukey’s test for multiple comparisons. (B) Representative H&E staining images of mice from the indicated groups; magnification: 200×; scale bar: 200 µm.

**sFigure 4. Pharmacological inhibition of eATP-P2X7 signaling attenuates pyroptosis and renal inflammation in DOCA-salt mice.** (A) Changes in the urinary albumin/creatinine ratio (UACR) in DOCA-salt mice following treatment with apyrase (0.2 U/g/d, by intraperitoneal injection) or AZ10606120 (0.5 mg/kg/d, by intraperitoneal injection); n = 4 in sham vehicle group and n = 6 in other experimental groups. (B) Representative images of H&E staining (magnification: 200×; scale bar: 200 µm), Sirius red staining (magnification: 200×; scale bar: 200 µm) and fluorescence images of P2X7 (red) and F4/80 (green) staining (magnification: 400×; scale bar: 100 µm) of kidney sections. (C) Summarized data of the tubular injury score; n = 4–5 per group. (D) Summarized collagen deposition index data (%); n = 4 per group and 10 independent fields per section. (E) Summarized data of the area of P2X7-F4/80 dual-positive cells in the kidney; n = 3 per group and 10 independent fields per section. (F) mRNA levels of *Fn*, *Vim*, *Ccl2* and *Il6* in the renal cortex; n = 4 in sham vehicle group and n = 5 in other experimental groups. (G) Representative western blotting images and (H) summarized data of pyroptosis-related proteins including NLRP3, cleaved Caspase-1 (c-Casp-1), GSDMD N-terminal, and mature IL-1β, in the kidney; n = 4 per group. (I) mRNA levels of *Cd86* and *Mrc1* in the renal cortex; n = 4 in sham vehicle group and n = 5 in other experimental groups. ^ns^p > 0.05, * p < 0.05, **p < 0.01, and ***p < 0.001 vs. the sham vehicle group; ^ns^p > 0.05, ^#^p < 0.05, ^##^p < 0.01, and ^###^p < 0.001 vs. the DOCA-salt vehicle group; one-way ANOVA with Tukey’s test for multiple comparisons.

**sFigure 5.** Summarized data from CCK-8 assays showing no cytotoxic effect of (A) finerenone (0.1–1 mM) and (B) PBN (0.1–2 mM) on mTECs; n = 3 per group; ^ns^p > 0.05, one-way ANOVA with Tukey’s test for multiple comparisons.

**sFigure 6. Activation of NOX4/ROS/cleaved Caspase-3/PANX1 signaling after Aldo stimulation.** (A) Representative images and summarized data of NOX4 and cleaved Caspase-3 (c-Casp-3) expression after Aldo stimulation (100 nM, 6 h) and finerenone (Fine) treatment (200 µM); n = 3 per group. (B) Representative images and summary data of the DCFH-DA intensity in mTECs after Aldo stimulation (100 nM, 6 h) and finerenone treatment (200 µM); n = 4–5 per group. (C) Relative Caspase-3 activity corrected by the Bradford method; n = 4 per group. (D) Representative images and summary data showing the effects of Aldo (100 nM, 6 h) and PBN (1 mM) treatment on the percentage of YO-PRO-1-positive mTECs; magnification: 200×; scale bar: 100 µm; n = 3 per group. One-way ANOVA with Tukey’s test for multiple comparisons was used. ^ns^p > 0.05, * p < 0.05, ** p < 0.01, and ***p < 0.001.

**sFigure 7. MR expression and its effect on mTECs and BMDMs.** (A) Representative images and summary data comparing MR expression in BMDMs and mTECs; n = 3 per group and assessed by an unpaired t test. (B) Representative images and summary data showing the effects of siRNA-mediated knockdown of *Nr3c2* in mTECs and BMDMs; n = 3 per group and assessed by an unpaired t test. (C) Representative images (magnification: 100×; scale bar: 200 µm) and summary data showing the change in BMDM migration following MR knockdown in mTECs or BMDMs after Aldo stimulation (100 nM, 24 h) in coculture experiments; n = 3 per group and assessed by one-way ANOVA with Tukey’s test for multiple comparisons. ^ns^p > 0.05, * p < 0.05, ** p < 0.01, and ***p < 0.001.

**sFigure 8. PANX1 knockdown in mTECs reduces BMDM migration** (A) Representative images and summary data showing the effects of PANX1 knockdown on mTECs; n = 4 per group; one-way ANOVA with Tukey’s test for multiple comparisons. (B) Representative images (magnification: 100×; scale bar: 200 µm) and summary data showing the effects of PANX1 knockdown in mTECs on BMDM migration in coculture experiments; n = 3 per group; two-way ANOVA with Tukey’s test for multiple comparisons. ^ns^p > 0.05, *p < 0.05, **p < 0.01, and ***p < 0.001.

**sFigure 9. Dose-dependent effects of apyrase and AZ10606120 on mTECs and BMDMs.** (A) CCK-8 results showing the effects of apyrase (0.1–1 U/mL) and AZ10606120 (10–100 µM) on mTEC and BMDM viability. n = 3 per group. (B) Dose-dependent effect of apyrase (0.1, 0.2, and 0.5 U/mL) on the ATP level in mTEC culture medium after Aldo stimulation (100 nM, 6 h). n = 3 per group. One-way ANOVA with Tukey’s test for multiple comparisons. ^ns^p > 0.05, *p < 0.05, **p < 0.01, and ***p < 0.001.

**sFigure 10. scRNA-seq analysis showing changes in the proportion of cell clusters and the expression of P1 and P2 receptors in DOCA-salt mice.** (A) The proportion of cell clusters in sham mice and DOCA-salt mice. (B) The expression of P1 and P2 receptors in sham mice and DOCA-salt mice; color density represents the relative expression level; relatively high-expressed genes are highlighted in red. UMAP plot of (C) *P2rx5*, (D) *P2ry6*, (E) *P2ry10b*, (F) *P2ry12*, (G) *P2ry13*, (H) *Adora2a*, and (I) *Adora2b* in sham mice and DOCA-salt mice. n = 3 per group.
